# CircMTO1 suppresses hepatocellular carcinoma progression via the miR-541-5p/ZIC1 axis by regulating Wnt/β-catenin signaling pathway and epithelial-to-mesenchymal transition

**DOI:** 10.1038/s41419-021-04464-3

**Published:** 2021-12-20

**Authors:** Dandan Li, Jiawei Zhang, Jing Yang, Jie Wang, Runling Zhang, Jinming Li, Rui Zhang

**Affiliations:** 1 National Center for Clinical Laboratories, Institute of Geriatric Medicine, Chinese Academy of Medical Sciences, Beijing Hospital / National Center of Gerontology, Beijing, P. R. China; 2 Graduate School, Peking Union Medical College, Chinese Academy of Medical Sciences, Beijing, 100730 P. R. China; 3 Beijing Engineering Research Center of Laboratory Medicine, Beijing, P. R. China

**Keywords:** Cancer therapy, Liver cancer, Tumour-suppressor proteins

## Abstract

CircRNA mitochondrial tRNA translation optimization 1 (circMTO1) functions as a tumor suppressor usually and is related to the progression of many tumors, including hepatocellular carcinoma (HCC). CircMTO1 is downregulated in HCC as compared to adjacent nontumor tissue, which may suppress the HCC progression by certain signal pathways. However, the underlying signal pathway remains largely unknown. The interactions between circMTO1 and miR-541-5p were predicted through bioinformatics analysis and verified using pull-down and dual-luciferase reporter assays. CCK-8, transwell, and apoptosis assays were performed to determine the effect of miR-541-5p on HCC progression. Using bioinformatic analysis, dual-luciferase reporter assay, RT-qPCR, and western blot, ZIC1 was found to be the downstream target gene of miR-541-5p. The regulatory mechanisms of circMTO1, miR-541-5p, and ZIC1 were investigated using in vitro and in vivo rescue experiments. The results depicted that silencing circMTO1 or upregulating miR-541-5p expression facilitated HCC cell proliferation, migration, and invasion and inhibited apoptosis. CircMTO1 silencing upregulated the expression of downstream ZIC1 regulators of the Wnt/β-catenin pathway markers, β-catenin, cyclin D1, c-myc, and the mesenchymal markers N-cadherin, Vimentin, and MMP2, while the epithelial marker E-cadherin was downregulated. MiR-541-5p knockdown had the opposite effect and reversed the effect of circMTO1 silencing on the regulation of downstream ZIC1 regulators. Intratumoral injection of miR-541-5p inhibitor suppressed tumor growth and reversed the effect of circMTO1 silencing on the promotion of tumor growth in HCC. These findings indicated that circMTO1 suppressed HCC progression via the circMTO1/ miR-541-5p/ZIC1 axis by regulating Wnt/β-catenin signaling and epithelial-to-mesenchymal transition, making it a novel therapeutic target.

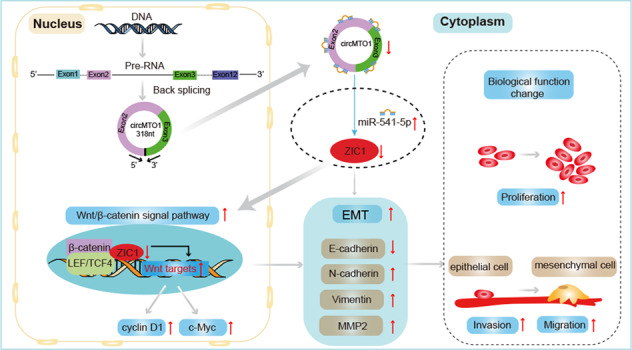

## Introduction

Hepatocellular carcinoma (HCC) is one of the most common diagnosed human malignancies worldwide, and its incidence is increasing each year [1]. China alone accounts for approximately 50% of the global liver cancer burden. An estimated 466,100 patients were diagnosed with HCC in China in 2015, of which 422,100 died [2, 3]. Resection, transplantation, and ablation are effective for early HCC treatment; however, most patients are diagnosed at advanced stages [4]. Although significant progress has been made in the treatment of liver cancer, HCC incidence and mortality remain high due to tumor recurrence, metastasis, drug resistance, and high adverse side effects, necessitating the identification of new targets for designing more powerful therapeutic approaches.

Recently, circular RNAs (circRNAs), a novel class of non-coding RNAs, have attracted attention [5]. Unlike linear RNAs, circRNAs form covalently closed-loop structures, characterized by stable, abundant, and specific expression in different tissues and cells during development [6]. CircRNAs act as key regulators in a wide range of biological processes, including microRNAs (miRNAs) sponge, RNA binding protein (RBP) sponges, protein/peptide translation templates, gene transcription, and RNA splicing regulators [7]. Mounting evidence shows that circRNAs play vital roles in cancer progression [8–10].

MiRNAs are another type of non-coding RNAs that act mainly via negative regulation of target genes at the post-transcription level by inhibiting protein translation or degrading target mRNAs [11]. MiRNAs can serve as either oncogene or tumor suppressors during the occurrence and development of various cancers [12, 13]. In recent years, a novel regulatory mechanism, called the competing endogenous RNA (ceRNA) hypothesis, has been proposed. CircRNAs have miRNA response elements (MREs) [14] and are known to regulate gene expression by acting as ceRNAs and subsequently counteract miRNA-induced effects on downstream mRNAs by sponging miRNAs, leading to tumorigenesis and development of cancer [15–17].

CircRNA mitochondrial tRNA translation optimization 1 (circMTO1, hsa_circRNA_0007874/hsa_circRNA_104135) originates from exons 2 and 3 of the MTO1 gene with a 318 bp splice length [18]. CircMTO1 usually functions as a tumor suppressor and is associated with the progression of multiple tumors [19], including glioblastoma [20], gallbladder cancer [21], lung adenocarcinoma [22], and HCC [23, 24]. CircMTO1 inhibits liver fibrosis in chronic hepatitis B patients via the regulation of miR-17-5p and Smad7 [25]. Silencing circMTO1 facilitates HCC cell proliferation, migration, and invasion and inhibits apoptosis in HCC. Han et al. found that circMTO1 suppressed HCC progression by acting as a sponge of oncogenic miR-9 to promote p21 expression [23]. Similarly, Wang et al. found that circMTO1 was downregulated in HCC tissues and cell lines compared with normal controls. The study also revealed that circMTO1 was a molecular sponge of miR-9-5p, and NOX4 was the target gene of miR-9-5p [24]. These two HCC studies revealed that circMTO1 acted as a miR-9 sponge and can regulate target mRNAs p21 and NOX4, respectively.

However, the circMTO1 signaling pathways involved in HCC progression have yet to be further explored. Interestingly, a study found that circMTO1 inhibited cell proliferation and invasion by regulating the Wnt/β-catenin signaling pathway in colorectal cancer [26]. In addition, circMTO1 suppressed bladder cancer metastasis by sponging miR-221 and, inhibited the epithelial-mesenchymal transition (EMT) process. It also inhibited EMT by sponging miR-3200-5p in gastric carcinoma (GC) [27]. Studies have also found that many circRNAs might function as ceRNAs to promote HCC progression by activating the Wnt/β-catenin pathway [28, 29] and EMT [30, 31]. However, whether circMTO1 affects Wnt/β-catenin signaling and EMT in the progression of HCC is not clear.

Moreover, a single circRNA usually contains multiple MREs, so the same circRNA can play a role in HCC by acting as a sponge of multiple miRNAs [32, 33]. For example, cSMARCA5 inhibited the growth and metastasis of HCC mainly by protecting TIMP3 from downregulation by miR-17-3p and miR-181b-5p [33]. We predicted that miR-541-5p may bind to circMTO1 using data from four databases. However, the role of miR-541-5p in HCC development has not yet been reported. The main target mRNA of miR-541-5p involved in liver cancer cell dysfunction requires further investigation. Through database prediction and experimental verification, ZIC1 was shown to be the target of miR-541-5p.

The aims of this study were to explore the function of miR-541-5p in HCC progression and to study whether circMTO1 functions as a suppressor of HCC progression via the miR-541-5p/ZIC1 axis by regulating Wnt/β-catenin signaling and the EMT process, which may provide a new research direction for targeted HCC therapy.

## Results

### The expression of has_circ_0007874 (circMTO1) was downregulated in HCC tissue

**Fig. 1:**
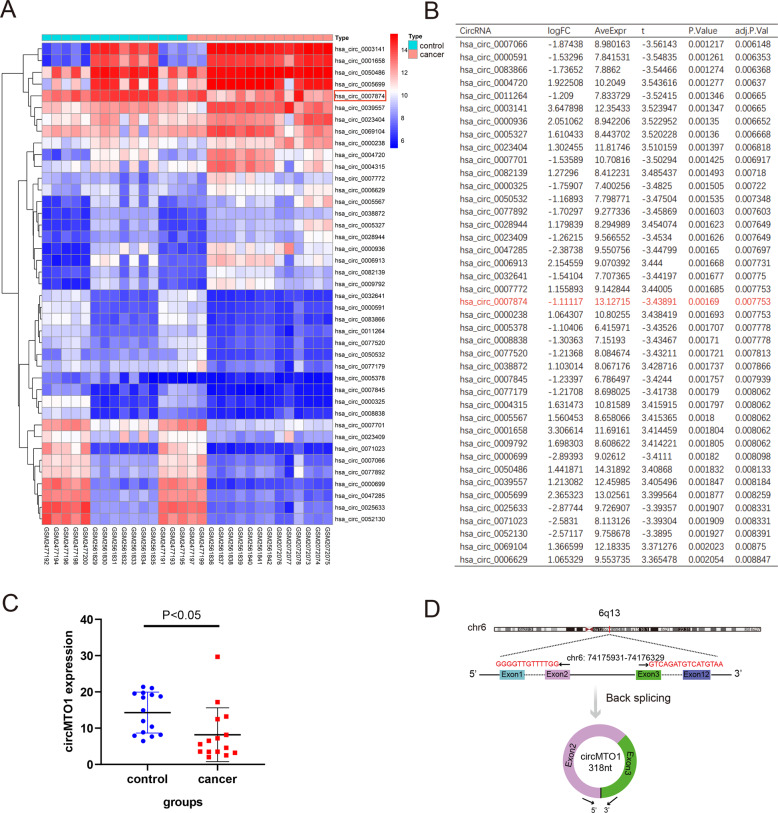
Screening for differential expression of circRNA. **A** Part of the downregulated differently expressed circRNAs and circMTO1 (hsa_circ_0007874) was highlighted in the red box. **B** Part of the differently expressed circRNAs shown using the logFC, and circMTO1 (hsa_circ_0007874) was highlighted in the words in red. **C** The level of circMTO1 was lower in the cancer group compared with the control group. **D** The pattern map of circMTO1 chromosome location and cyclization. LogFC: The expression ratio of has_circ_0007874(circMTO1) in cancer and control groups.

Three microarrays (GSE97332, GSE94508, and GSE78520) from the GEO database containing 15 HCC patients and 15 normal controls were combined. There were 522 differentially expressed circRNAs, including 257 upregulated and 265 downregulated circRNAs (Table S1). A heatmap showing part of the downregulated genes was shown in Fig. 1A. CircMTO1 expression was lower in the tumor group than in the normal group (P < 0.05) (Fig. 1B, C). Fig 1D showed that circMTO1 is located on chr 6 and the circularization of MTO1 exons 2 and 3 forms circMTO1.

### CircMTO1 acted as a miRNA sponge of miR-541-5p in HCC

**Fig. 2:**
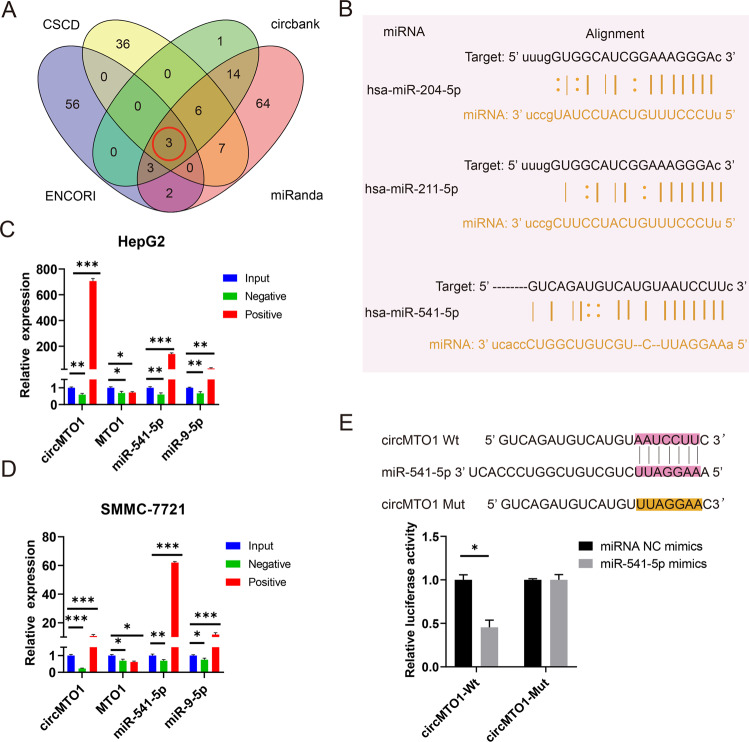
miR-541-5p was regulated by circMTO1. **A** The intersected miRNAs were predicted from the four databases. **B** ENCORI database-predicted sites through which miRNAs bind to circMTO1. **C**, **D** Pull-down assays were performed using circMTO1 probes in HepG2 and SMMC-7721 cells, and circMTO1, linear MTO1, and miRNAs enrichment was detected using RT-qPCR. **E** HEK293T cells were co-transfected with miR-541-5p mimics and Wt or Mut circMTO1 luciferase reporter vectors, and luciferase reporter activity was detected. Data were presented as mean ± SD of three independent experiments. *P < 0.05, **P < 0.01, ***P < 0.001.

Studies have shown that circRNAs can function as miRNA sponges that competitively bind to miRNA, thus abrogating the inhibitory effect of miRNAs on downstream target genes [28, 34]. Since circMTO1 is located in the cytoplasm [23], we studied whether it could function as a miRNA sponge, except miR-9, in HCC cells. The ENCORI/starbase, CSCD, circbank, and miRanda databases were used to predict the miRNAs that may bind to circMTO1 (Fig. 2A and Table S2). Three miRNAs: miR-204-5p, miR-211-5p and miR-541-5p, were predicated from the four databases and taken as possible circMTO1 targets. The ENCORI database-predicted sites where miRNA might bind to circMTO1(Fig. 2B). CircMTO1 is downregulated in HCC as compared to adjacent nontumor, according to the ceRNA theory, miRNAs are negatively correlated with the expression of circRNAs. Studies have shown that miRNA-204-5p [35, 36] and miRNA-211-5p [37, 38] function as tumor suppressor factors, thus miRNA-541-5p was chosen for the pull-down assay. MiR-9, which has been reported as a target of circMTO1, was selected as a positive control in the pull-down assay. The pull-down assay results showed that the expressions of circMTO1, miR-541-5p, and miR-9 were enriched in HCC cell lines compared to that in controls, and the expression of miR-541-5p was relatively greater than that of miR-9, whereas the linear MTO1 had no enrichment (Fig. 2C, D). A dual-luciferase reporter assay was used to verify whether miR-541-5p could bind to circMTO1. We constructed luciferase reporter vectors for wildtype (Wt) circMTO1 and miR-541-5p binding-site mutant (Mut) circMTO1 and co-transfected HEK293T cells with miR-541-5p mimics or NC mimics. Luciferase activity in the Wt group co-transfected with miR-541-5p mimics was significantly reduced, while luciferase activity in the Mut group did not change significantly (Fig. 2E). Finally, miRNA-541-5p was verified as the direct target of circMTO1.

### Effects of miR‑541‑5p on proliferation, migration, and invasion of HCC cells in vitro

**Fig. 3:**
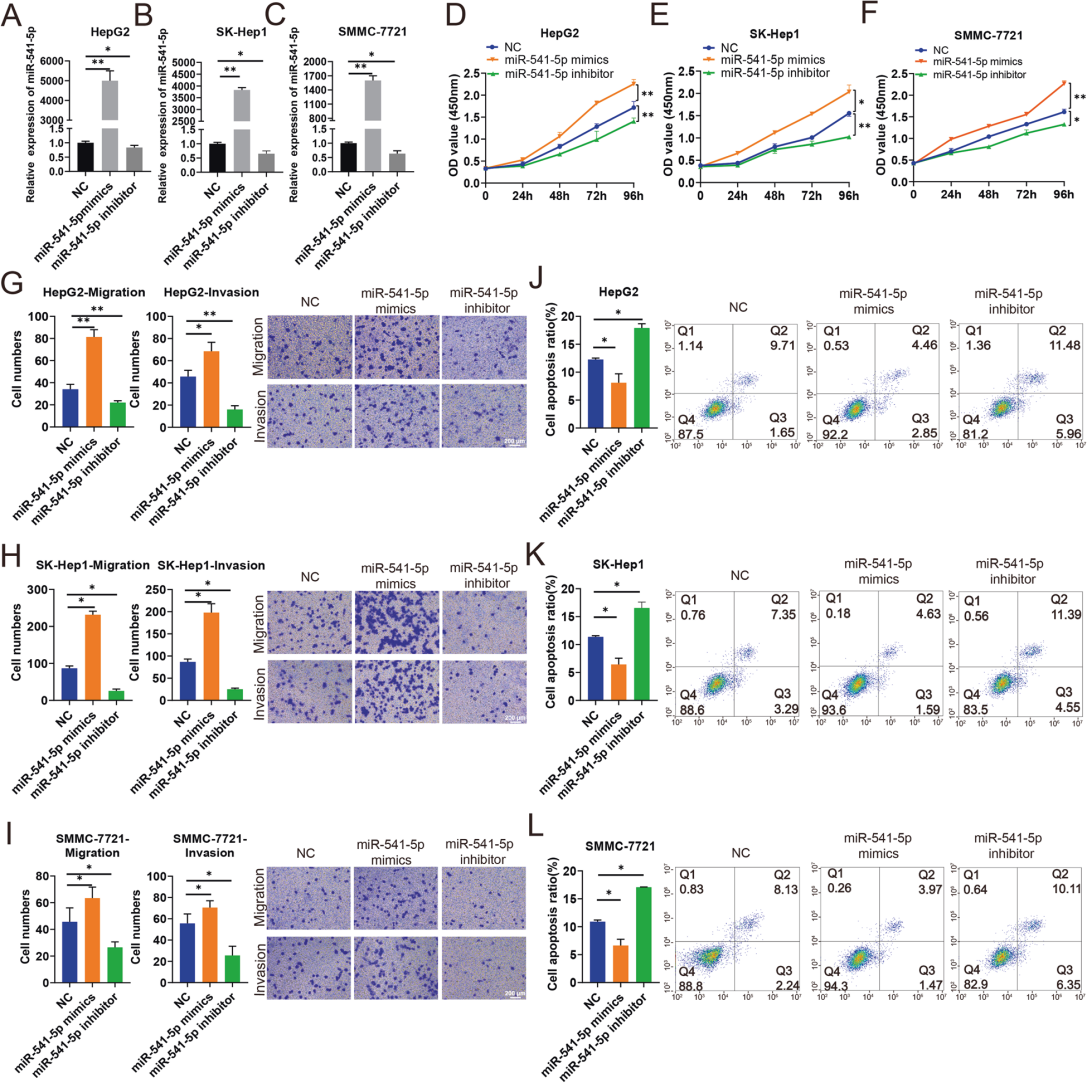
MiR-541-5p promotes proliferation, migration, and invasion but inhibited apoptosis of HepG2, SK-Hep1, and SMMC-7721 cell lines. HepG2, SK-Hep1, and SMMC-7721 cells were transfected with NC, miR-541-5p mimics, and miR-541-5p inhibitor. **A–C** Expression of miR-541-5p in HepG2, SK-Hep1, and SMMC-7721 cells. **D–F** Cell proliferation was detected in HepG2, SK-Hep1, and SMMC-7721 cells using CCK-8 assay. **G–I** Effect of miR-541-5p on migration and invasion were assessed using transwell migration and invasion assays; Scale bar, 200 μm. **J–L** Cells were stained with Annexin V-APC and PI, and the percentage of apoptotic cells was determined through flow cytometry. Data were presented as mean ± SD of three independent experiments. *P < 0.05; **P < 0.01.

To better understand the function of miR-541-5p, we performed CCK-8, transwell, and apoptosis assays to investigate the effects of miR-541-5p on proliferation, migration, and invasion of HCC cells. HepG2, SK-Hep1, and SMMC-7721 cells were transfected with miR-541-5p mimics, miR-541-5p inhibitor, or negative control (NC). RT-qPCR was performed to verify transfection efficiency. The results showed that miR-541-5p expression was significantly upregulated after transfecting HepG2, SK-Hep1, and SMMC-7721 cells with miR-541-5p mimics (all *P* < 0.01), with a thousand-fold increase compared with the control group. MiR-541-5p expression was downregulated in HepG2, SK-Hep1, and SMMC-7721 cells transfected with miR-541-5p inhibitor (all *P* < 0.05) (Fig. 3A–C). CCK-8 experiments showed that miR-541-5p overexpression promoted cell proliferation of HepG2, SK-Hep1, and SMMC-7721 cells compared with the NC and miR-541-5p knockdown inhibited cell proliferation compared with the NC (Fig. 3D–F). The Transwell assay also showed that migration and invasion of HCC cells were significantly enhanced after overexpression of miR-541-5p, whereas migration and invasion of HCC cells were significantly attenuated after miR-541-5p silencing (Fig. 3G–I). However, apoptosis assay showed that miR-541-5p overexpression suppressed the apoptosis of HepG2, SK-Hep1, and SMMC-7721 cells, and inhibition of miR-541-5p promoted the apoptosis of HepG2, SK-Hep1, and SMMC-7721 cells (all *P* < 0.05) (Fig. 3J–L).

### MiR-541-5p reversed the suppressive effect of circMTO1 in HCC cells

**Fig. 4:**
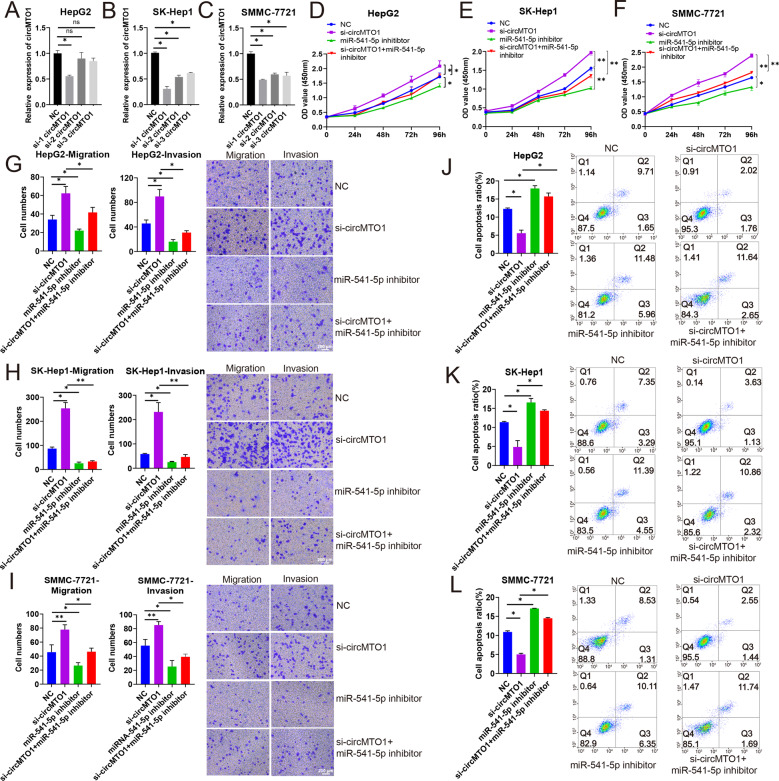
CircMTO1 regulated miR-541-5p and affected the proliferation, migration, invasion, and apoptosis of HCC cells. HepG2, SK-Hep1, and SMMC-7721 cells were transfected with NC, circMTO1 siRNAs, and/or miR-541-5p inhibitor. **A–C** Analysis of circMTO1 expression using RT-qPCR after transfection with circMTO1 siRNAs. **D–F** Cell proliferation was determined using CCK-8 assay. **G–I** The migration and invasion of HepG2, SK-Hep1, and SMMC-7721 cells were evaluated using transwell assays. Scale bar; 200 μm. **J–L** Cells were stained with Annexin V-APC and PI, and the percentage of apoptotic cells was measured using flow cytometry. Data were presented as mean ± SD of three independent experiments. *P < 0.05; **P < 0.01.

We conducted a rescue experiment to explore the suppression mechanism of circMTO1 in HCC working as a miR-541-5p sponge. HepG2, SK-Hep1, and SMMC-7721 cells were transfected with three circMTO1 siRNAs. Among the three circMTO1 siRNAs, si-1 circMTO1 had the highest silencing efficiency in HepG2, SK-Hep1, and SMMC-7721 cells (Fig. 4A–C). Resultingly, it was selected for further study. The results of the CCK-8 assay showed that circMTO1 silencing enhanced the proliferation of HepG2, SK-Hep1, and SMMC-7721 cells and the proliferation of HCC cells was significantly decreased following miR-541-5p inhibitor transfection (*P* < 0.05). Transfection with miR-541-5p inhibitor compromised the enhanced proliferation caused by circMTO1 silencing (Fig. 4D–F). Transwell assays demonstrated that miR-541-5p inhibition reversed the promotion of migration and invasion of HepG2, SK-Hep1, and SMMC-7721 cells caused by circMTO1 knockdown (Fig. 4G–I). Compared with cells transfected with si-circMTO1 alone, co-transfection of HepG2, SK-Hep1, and SMMC-7721 cells with si-circMTO1 and miR-541-5p inhibitor significantly promoted apoptosis (all *P* < 0.05) (Fig. 4J–L). Taken together, these results indicated that circMTO1 suppressed the malignant progression of HCC cells by abolishing the tumorigenic effect of miR-541-5p.

### ZIC1 was a direct target of miR-541-5p

**Fig. 5:**
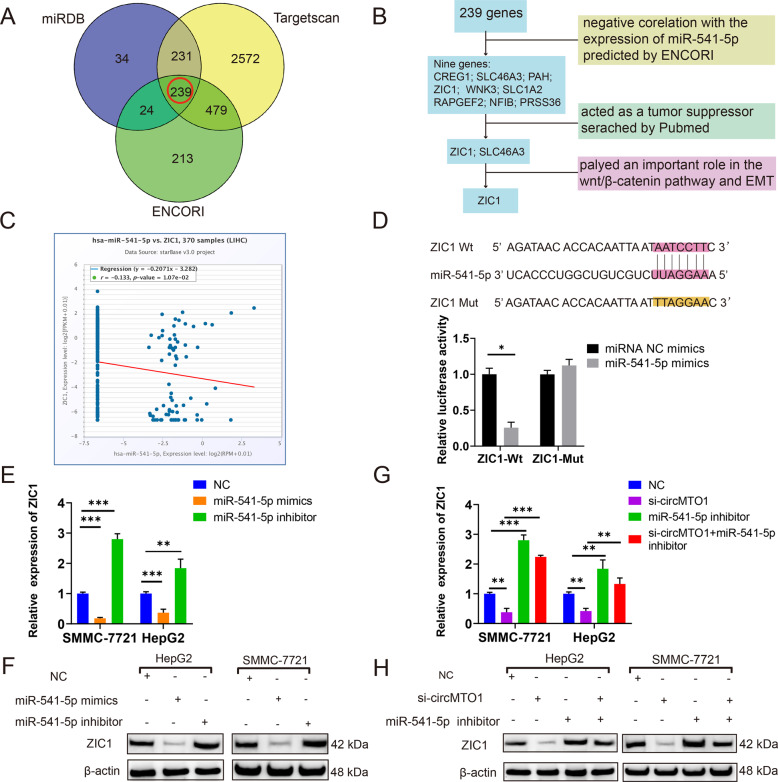
ZIC1 was directly targeted by miR-541-5p. **A**, miRDB, TargetScan, and ENCORI databases were used to predict potential mRNA targets of miR-541-5p. **B** The screening process of miR-541-5p target candidate. **C** The co-expression of miR-541-5p and ZIC1 as predicted by ENCORI in HCC. **D** Schematic illustration of ZIC1 3′UTR-Wt and 3′UTR-Mut luciferase reporter vectors. **E**, **F** Relative ZIC1 mRNA and protein levels were detected in cells using RT-qPCR and western blot, respectively, after transfection with miR-NC, miR-541-5p mimics, and miR-541-5p inhibitor. **G**, **H** Relative ZIC1 mRNA and protein levels were detected in cells using RT-qPCR and western blot, respectively, after transfection with NC, si-circMTO1, miR-541-5p inhibitor, and si-circMTO1 + miR-541-5p inhibitor. Data were presented as mean ± SD of three independent experiments, *P < 0.05, **P < 0.01, ***P < 0.001.

Potential miR-541-5p target genes were predicted using miRDB, TargetScan, and ENCORI databases (Table S3), and 239 genes were identified (Fig. 5A and Table S4). According to the ceRNA theory, circMTO1 is positively correlated with the expression of downstream target genes, and the target genes are negatively correlated with the expression of miR-541-5p and the downstream factors function as tumor suppressors as well as circMTO1. We analyzed the 239 genes in the miRNA-mRNA section of the ENCORI database and found nine genes whose expressions were negatively correlated with miR-541-5p. We conducted a literature search for the nine mRNAs in the PubMed database. Among them, ZIC1 and SLC46A3 have been shown to function as tumor suppressors and ZIC1 was found to play an important role in the Wnt/β-catenin pathway and EMT. Therefore, ZIC1 was chosen as the candidate mRNA (Fig. 5B, C). A luciferase reporter assay was conducted to verify that ZIC1 was the downstream factor of miR-541-5p. MiR-541-5p interacts directly with the 3’UTR of mRNA, thus we constructed Wt ZIC1 3′ UTR and miR-541-5p binding-site Mut luciferase reporter plasmids. The Wt and Mut reporter vectors were co-transfected into cells together with the miR-541-5p mimics. The results showed that the relative luciferase activity of miR-541-5p mimics and the ZIC1 Wt group was lower (*P* < 0.05), while the luciferase activity of the Mut group did not change significantly (Fig. 5D). Thus, ZIC1 was confirmed as a downstream factor of miR-541-5p. In addition, overexpression of miR-541-5p significantly decreased the expression of ZIC1 mRNA and protein, whereas downregulation of miR-541-5p increased ZIC1 expression in SMMC-7721 and HepG2 cells (Fig. 5E, F). Moreover, ZIC1 levels decreased when SMMC-7721 and HepG2 cells were transfected with si-circMTO1. Compared with the circMTO1 silencing group, HCC lines co-transfected with si-circMTO1 and miR-541-5p inhibitor showed higher levels of ZIC1 mRNA and protein (Fig. 5G, H).

### CircMTO1 inhibited the malignant progression of HCC by miR-541-5p/ZIC1 axis by regulating Wnt/β-catenin signaling and EMT

**Fig. 6:**
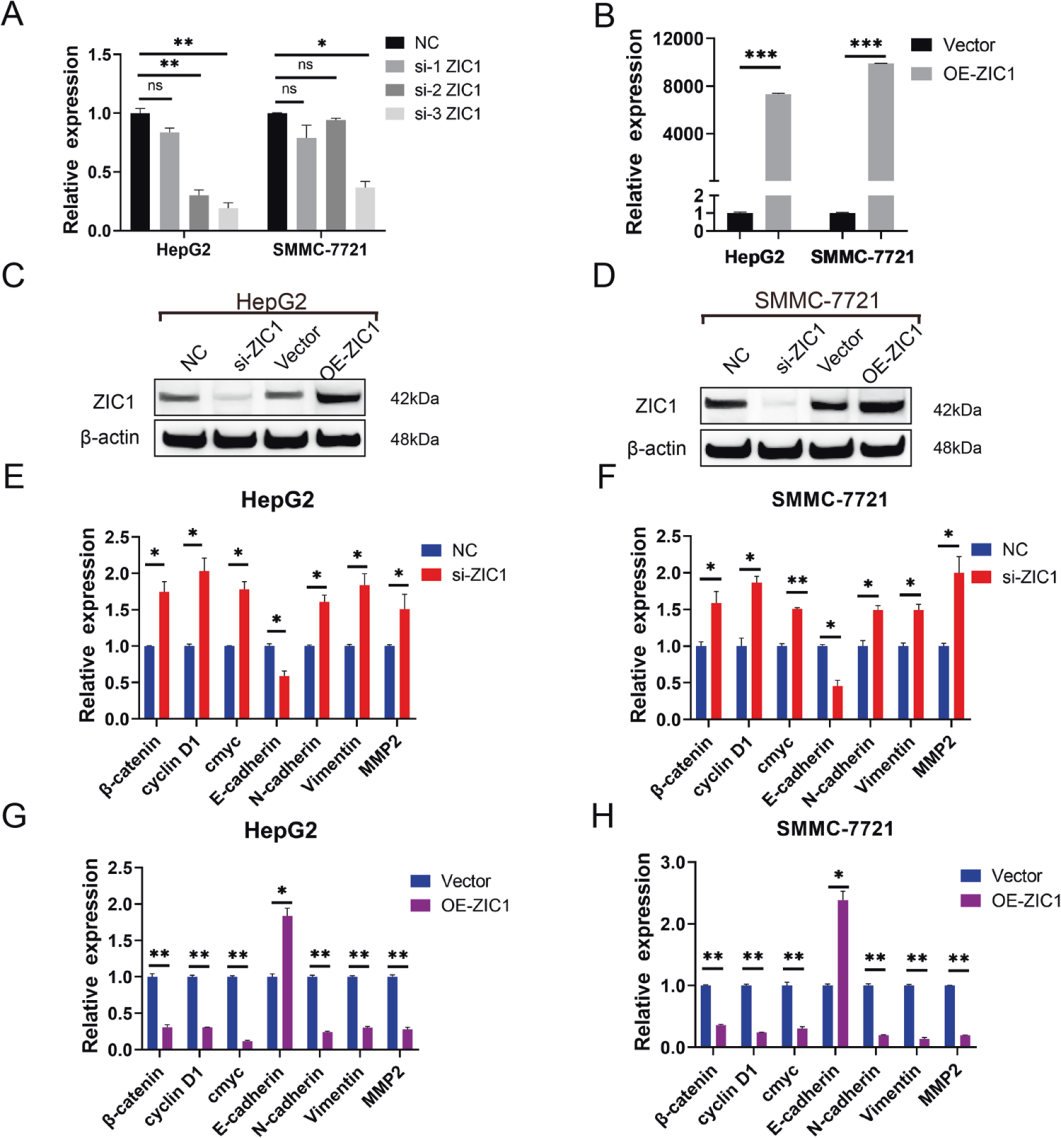
Modulation of ZIC1 and the effect on the indicated downstream mediators of Wnt/β-catenin signaling and EMT. **A**–**D** SMMC-7721 and HepG2 cells were transfected with ZIC1 siRNAs or ZIC1-overexpressing plasmid (OE-ZIC1). **A**, **B** Analysis of ZIC1 expression via RT-qPCR. **C**, **D** Analysis of ZIC1 expression via western blot. Vector: the empty pcDNA3.1(+) plasmid. **E**, **F** β-catenin, cyclin D1, c-myc, E-cadherin, N-cadherin, Vimentin, and MMP2 mRNA expression were detected via RT-qPCR after transfection with si-ZIC1. **G**, **H** β-catenin, cyclin D1, c-myc, E-cadherin, N-cadherin, Vimentin, and MMP2 mRNA expression were detected via RT-qPCR after transfection with OE-ZIC1. Data were presented as mean ± SD of three independent experiments, *P < 0.05, **P < 0.01, ***P < 0.001.

**Fig. 7:**
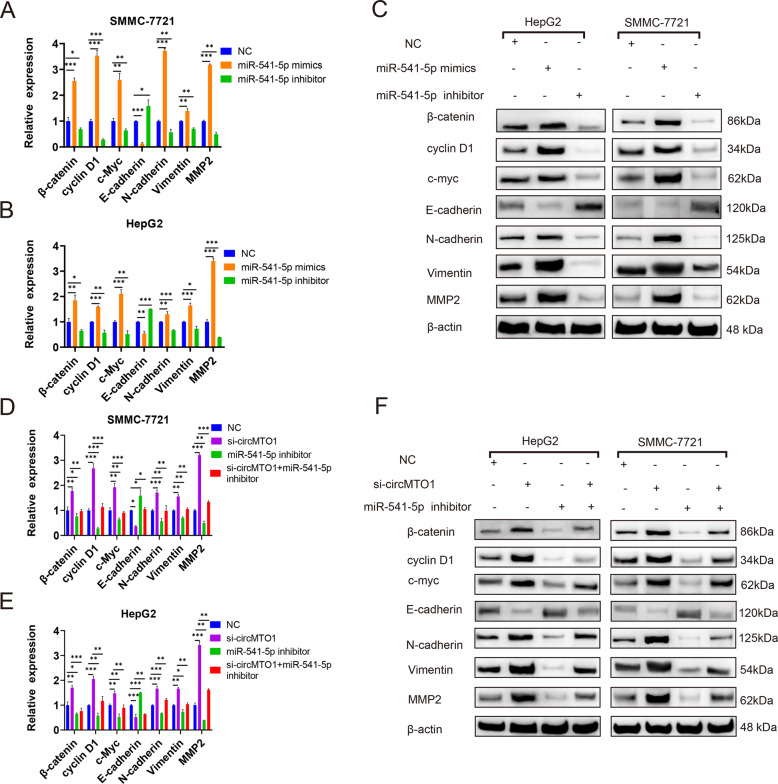
CircMTO1 inhibited the malignant progression of HCC via miR-541-5p/ZIC1 by regulating Wnt/β-catenin signaling and EMT. **A–C** SMMC-7721 and HepG2 cells were transfected with miR-541-5p mimics or miR-541-5p inhibitor. **A-C** β-catenin, cyclin D1, c-myc, E-cadherin, N-cadherin, Vimentin, and MMP2 mRNA and protein expression were detected through RT-qPCR and western blot, respectively. **D–F** SMMC-7721 and HepG2 cells were transfected with si-circMTO1 or co-transfected with si-circMTO1 and miR-541-5p inhibitor **D–F**, β-catenin, cyclin D1, c-myc, E-cadherin, N-cadherin, Vimentin, and MMP2 mRNA and protein expression were detected through RT-qPCR and western blot, respectively. Data were presented as mean ± SD of three independent experiments, *P < 0.05, **P < 0.01, ***P < 0.001.

ZIC1 was modulated to determine whether it affected Wnt/β-catenin signaling and EMT downstream factors. Among the three ZIC1 siRNAs, si-3 ZIC1 had the highest silencing efficiency in HepG2 and SMMC-7721 cells (Fig. 6A), and therefore, it was selected for further study. ZIC1 expression was lower after transfection with si-ZIC1 (Fig. 6A, C) and was significantly upregulated after the transfection of HepG2 and SMMC-7721 cells with the ZIC1-overexpressing plasmid (OE-ZIC1) compared with that in the vector group (Fig. 6B–D). ZIC1 silencing significantly upregulated the expression of β-catenin, cyclin D1, and c-myc, which are key factors in the Wnt/β-catenin signaling pathway, and N-cadherin, Vimentin, and MMP2 expression, which are key factors in EMT; however, the level of E-cadherin, which is the key biomarker in EMT, decreased (Fig. 6E, F). The key factors of the OE-ZIC1 group exhibited the opposite trend (Fig. 6G, H). The next step was to clarify whether circMTO1 inhibited the malignant progression of HCC through the miR-541-5p/ZIC1 axis by regulating Wnt/β-catenin signaling and EMT. When SMMC-7721 and HepG2 cells were transfected with miR-541-5p mimics or si-circMTO1, the expression of downstream ZIC1 regulators, β-catenin, cyclin D1, c-myc, N-cadherin, Vimentin, and MMP2 were upregulated; however, the level of E-cadherin was downregulated (Fig. 7). The downregulation of miR-541-5p produced the opposite results (Fig. 7A–C). And the expression of downstream ZIC1 regulators, β-catenin, cyclin D1, c-myc, N-cadherin, Vimentin, and MMP2 were significantly decreased after co-transfection with the miR-541-5p inhibitor and si-circMTO1 compared with transfection with si-circMTO1 alone. However, the expression of E-cadherin increased (Fig. 7D–F).

### The circMTO1/miR-541-5p/ZIC1 axis inhibited tumor growth in vivo and was a potential therapeutic target

**Fig. 8:**
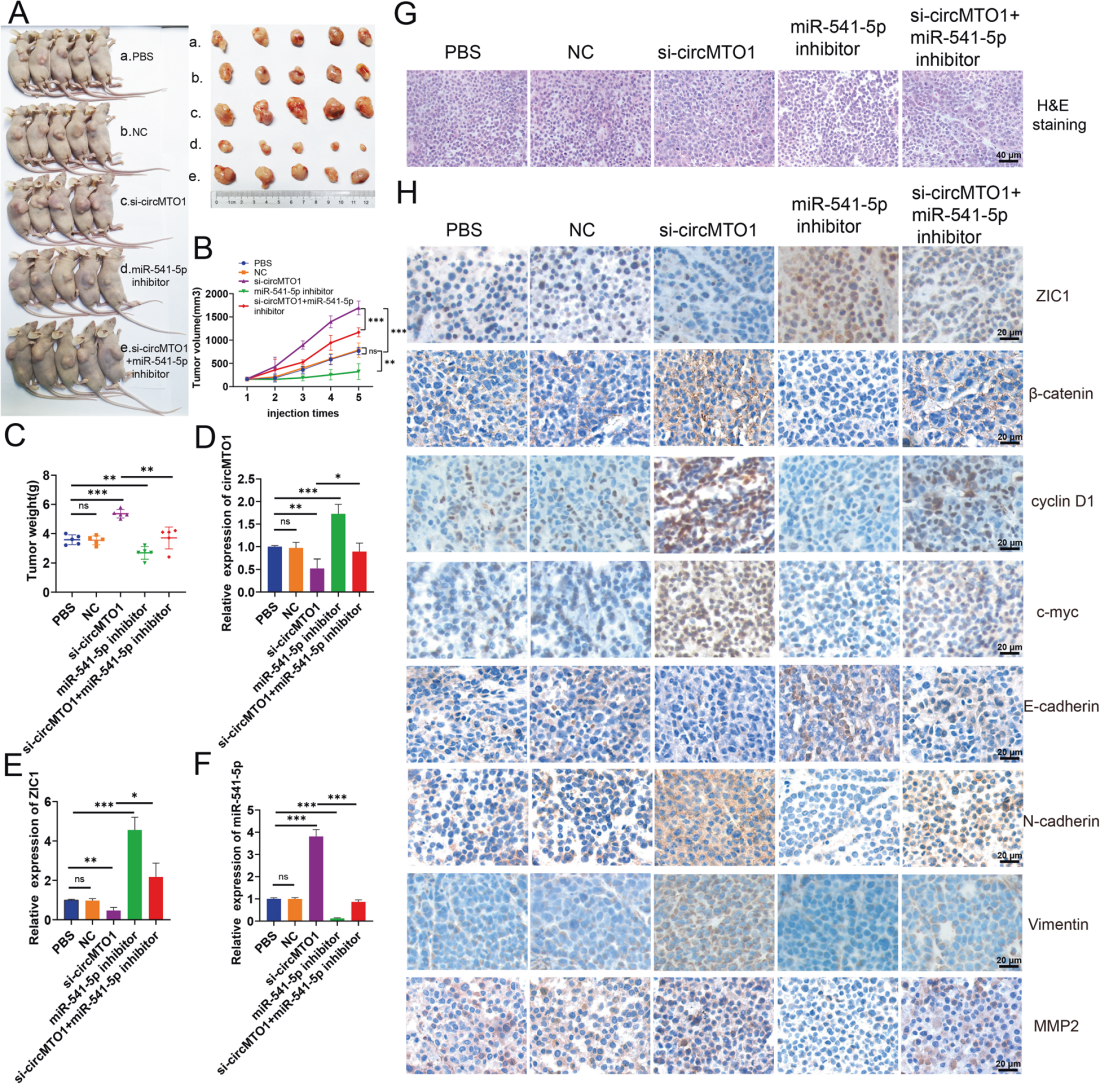
CircMTO1/miR-541-5p/ZIC1 axis inhibited HCC tumor growth in vivo. **A** Nude mice were subcutaneously injected with (a.) PBS, (b.) cholesterol-conjugated negative control (NC), (c.) cholesterol-conjugated si-circMTO1, (d.) cholesterol-conjugated inhibitor (e.) cholesterol-conjugated si-circMTO1 + cholesterol-conjugated inhibitor. After five injections, tumors were dissected and imaged. **B** Before injection, the tumor volume was measured and the tumor growth curve plotted. **C** Tumor weight was calculated on the day the mice were killed. Data represents mean ± SD (*n* = 5 per group). **D–F** Expression levels of circMTO1, miR-541-5p, and ZIC1 in xenograft tumors were measured using RT-qPCR. **G** H&E staining revealed the structure of xenograft tumors. Scale bar; 40 μm. **H** Changes in ZIC1, β-catenin, c-myc, cyclin D1, E-cadherin, N-cadherin, Vimentin, and MMP2 expression in xenograft tumors were detected through IHC staining. Scale bar; 20 μm. *P < 0.05; **P < 0.01; ***P < 0.001.

To investigate whether the circMTO1/ miR-541-5p /ZIC1 axis can serve as a promising therapeutic target, we constructed xenograft tumor models of nude mice by subcutaneously injecting them with SMMC-7721 cells. After intratumoral injection of cholesterol-conjugated circMTO1 siRNAs, tumor growth was significantly accelerated and the tumor weight was also significantly greater than in the PBS and NC groups (Fig. 8A–C). After intratumoral injection of cholesterol-conjugated miR-541-5p inhibitor, tumor growth was inhibited and tumor weight was less than in the PBS and NC groups. This reversed the promoting effect of circMTO1 silencing on tumor growth (Fig. 8A–C). Total RNA extracted from xenograft tumors and RT-qPCR was used to measure the expression of circMTO1, miR-541-5p, and ZIC1. The results showed decreased circMTO1 and ZIC1 expression, while miR-541-5p expression was increased in tumors with circMTO1 silencing (Fig. 8D–F). The expression of miR-541-5p was lower than that in the PBS and NC groups after miR-541-5p knockdown, while the expression of ZIC1 was higher than that in the PBS and NC groups (Fig. 8E, F). Furthermore, H&E staining showed that circMTO1 knockdown increased the nuclear volume and varied the size, and the frequency of nuclear division was also higher than in the other groups (Fig. 8G). IHC staining demonstrated that the expression of ZIC1 and the EMT marker E-cadherin were downregulated in si-circMTO1 xenograft tumors (Fig. 8H). In addition, when the expression of circMTO1 decreased, the Wnt/β-catenin signaling markers, β-catenin, c-myc, cyclin D1 and the EMT markers N-cadherin, Vimentin, and MMP2 were higher than those in the PBS and NC groups, while the Wnt/β-catenin signaling markers β-catenin, c-myc, cyclin D1, and the EMT markers N-cadherin, Vimentin, and MMP2 were lower than those in the PBS and NC groups with a miR-541-5p knockdown. These data confirmed that circMTO1 suppressed the malignant progression of HCC in vivo via the miR-541-5p/ZIC1 axis by regulating Wnt/β-catenin signaling and EMT.

## Discussion

As novel gene regulators, circRNAs exert their roles at the transcriptional or post-transcriptional levels to regulate downstream factors [39]. Increasing evidence suggests that circRNAs can be differently expressed in tumor tissues and can participate in HCC progression [31, 40, 41], working as biomarkers for HCC therapeutic targets or prognosis [42]. A growing body of evidence shows that circMTO1 can function as a suppressor in multiple cancers [43, 44]. Han et al showed that decreased circMTO1 expression was correlated with poor prognosis in a cohort of 116 HCC patients [23]. Therefore, in this study, we did not explore the prognostic function of circMTO1, and data from loss-of-function experiments have indicated that circMTO1 silencing can promote proliferation, migration, and invasion of HCC cells while inhibiting apoptosis. Instead, we focused on identifying the downregulated miRNA of circMTO1 to explore promising therapeutic targets. In this study, we elucidated a new mechanism through which circMTO1 suppresses HCC progression via the miR-541-5p/ZIC1 axis by regulating Wnt/β-catenin signaling and EMT.

MiRNA-541-5p was identified as a direct target of circMTO1. To the best of our knowledge, this study is the first to explore the effect of miR-541-5p on HCC progression. Here, three different cell lines (HepG2, SMMC-7721, and SK-Hep1) were used to improve the robustness of the study and the results were consistent, revealing that miR-541-5p promoted proliferation, migration, and invasion of HCC cells and inhibited apoptosis in vitro. Further, a rescue experiment was carried out to explore whether circMTO1 suppresses HCC by acting as a miR-541-5p sponge. The rescue experiments revealed that in the circMTO1 and miR-541-5p inhibitor co-transfected system, miR-541-5p inhibitor attenuated the effect of downregulated circMTO1 on cell proliferation, migration, invasion, and apoptosis, suggesting that circMTO1 can regulate miR-541-5p and affect HCC progression.

As previously mentioned, miRNAs are also considered a class of non-coding RNAs and are 22–25 nucleotides long [45]. They exert their functions by targeting 3’UTRs of mRNA post-transcriptionally and repressing gene expression [46]. In our study, the dual-luciferase reporter assay identified ZIC1 as the direct target of miR-541-5p. ZIC1 finger of cerebellum 1 (ZIC1) is located on chromosome 3q25.1, belongs to the ZIC family, which encodes a zinc-finger transcription factor and serves as a transcriptional cofactor [47]. Ge et al. demonstrated that lower expression of ZIC1 was correlated with increased lymph node metastasis and poor outcome in GC patients. Mechanistically, ZIC1 can downregulate Wnt targets including c-Myc and cyclin D1 by attenuating the β-catenin/ transcription factor 4 (TCF4) complex and modulating the EMT process in GC cells [48]. EMT also contributes to the progression of the disease, including organ fibrosis and cancer, and has been recognized as a key step in the progression of cancer metastasis [49]. Furthermore, Wnt/β-catenin signaling has a major impact on EMT. Nuclear β-catenin binds to members of the TCF/LEF family of transcription factors to promote EMT [49]. ZIC1 is an essential regulator of the Wnt/β-catenin and EMT signaling networks. Results from RT-qPCR and western blot (Fig. 5G, H) revealed that ZIC1 expression can be regulated by circMTO1 through miR-541-5p. Moreover, circMTO1 competitively bound to miR-541-5p and relieved the inhibitory effect of miR-541-5p on ZIC1 expression, thereby upregulating ZIC1 expression. CircMTO1 silencing increased the expression of Wnt/β-catenin downstream markers such as β-catenin, cyclin D1, c-myc, and mesenchymal markers such as N-cadherin, vimentin, and MMP2, while the epithelial marker, E-cadherin, was downregulated. MiR-541-5p knockdown had the opposite effect and reversed the effect of circMTO1 silencing on the expression of downstream ZIC1 regulator, indicating that the circMTO1 /miR-541-5p /ZIC1 axis suppressed proliferation, migration, and invasion by regulating the Wnt/β-catenin pathway and EMT.

In further in vivo studies, we verified the regulatory relationship of the circMTO1/miR-541-5p/ZIC1 axis. CeRNAs can compete with mRNAs, when miRNAs combine with ceRNAs, miRNAs and circRNAs expression levels are usually reversed. The mRNAs transcription level regulated by miRNAs are consistent with the expression level of circRNAs. Our in vivo results proved that miR-541-5p increased when circMTO1 was knocked down, while the expression of ZIC1 decreased (Fig. 8E, F). MiRNAs are known as non-coding RNAs, and they can be used as biomarkers for HCC treatment [50, 51]. In our in vivo study, tumor growth was inhibited after intratumoral injection of cholesterol-conjugated miR-541-5p inhibitor but increased by intratumoral injection of cholesterol-conjugated circMTO1 siRNA, indicating that the in vivo intervention of circMTO1/miR-541-5p/ZIC1 axis could be a potential target in HCC targeted therapy.

In order to gain a deeper understanding of the role of circMTO1, further investigation of its biogenesis mediated by pre-mRNA splicing needs to be explored. We searched circinteractome (https: //circinteractome. nia. nih. gov/index. html), three RBPs: DGCR8, eukaryotic initiation factor 4A3 (EIF4A3), and U2AF65 were predicted to have putative binding sites matching the flanking regions of circMTO1. A previous study showed that the sequence located upstream of circMMP9, which contained EIF4A3 binding sites, was important for the interaction between EIF4A3 and MMP9 mRNA. EIF4A3 bound to the MMP9 mRNA transcript, inducing circMMP9 cyclization and increasing circMMP9 expression in glioblastoma multiforme [52]. Moreover, EIF4A3 was found to be a core component of the exon junction complex and played an essential role in pre-mRNA splicing. The RT-qPCR assay showed that overexpression of EIF4A3 facilitated the expression of circSEPT9, while EIF4A3 knockdown inhibited circSEPT9 expression in triple-negative breast cancer cells [53]. Exploring whether EIF4A3 with an effect on circMTO1 biogenesis is of great significance and should be pursued in future studies to develop non-invasive circMTO1 detection methods for HCC that can be used in clinical applications.

In summary, our study showed that miR-541-5p promoted the proliferation, migration, and invasion and inhibited apoptosis of HCC cells. Functionally and mechanistically, circMTO1 competitively bound miR-541-5p and eliminated its inhibitory effect on ZIC1, thereby suppressing HCC cell proliferation, migration, and invasion by regulating the Wnt/β-catenin signaling pathway and EMT both in vitro *and* in vivo. These findings provide new insights into the occurrence and progression of HCC and indicate that the circMTO1/miR-541-5p/ZIC1 axis may serve as a potential therapeutic target for the treatment of HCC.

## Materials and methods

### Microarray data information from the Gene Expression Omnibus (GEO) database for the screening study

The gene expression profiles from GSE97332 (including seven adjacent nontumorous samples and seven HCC samples; platform: GPL19978), GSE94508 (including five adjacent nontumorous samples and five HCC samples; platform: GPL19978), GSE78520 (including three liver cancer tissue and three normal liver tissue; platform: GPL19978) were obtained from the GEO database (http://www.ncbi.nlm.nih.gov/geo/). We combined the three microarrays, including 15 liver cancer patients and 15 controls to screen the differential expression of circRNAs. CircRNAs exhibiting fold changes (FCs) ≥2.0 with *P* values <0.05 were considered differentially expressed.

### Cell lines and cultivate

Three HCC cell lines (SK-Hep1, SMMC-7721, and HepG2) and HEK293T cells were purchased from Shanghai Xinyu Biological Technology Co., Ltd. SMMC-7721 cells were cultured in RPMI-1640 medium (Gibco, Carlsbad, CA, USA) supplemented with 10% FBS (Gibco, Carlsbad, CA, USA), 100 U/mL penicillin, and 100 μg/mL streptomycin. HepG2 cells and HEK293T cells were cultured in DMEM (Gibco, Carlsbad, CA, USA) supplemented as above. SK-Hep1 cells was cultured in MEM (Gibco, Carlsbad, CA, USA) supplemented as above. All cell lines were cultured at 37 °C under 5% CO_2_. The medium was replaced every 2 days and the cells in the log phase were used for the experiments.

### RNA extraction and quality control

Total RNA was extracted with Trizol reagent (Invitrogen, USA) according to the manufacturer’s instructions. A NanoDrop ND-2000 instrument (Thermo Fisher Scientific, USA) was used to evaluate the quality of the RNA samples.

### Reverse transcription real-time quantitative PCR (RT-qPCR)

Total RNA was isolated and 2 μg of total RNA was used in reverse transcription with PrimeScript RT Reagent Kit (Takara, Dalian, China) according to the manufacturer’s protocol. RT-qPCR was conducted on the ABI Prism 7500 system (Applied Biosystems, CA, USA) with SYBR Green Premix Ex Taq (Takara, Dalian, China). GAPDH was used as an internal control for circRNA and mRNA, while U6 was for miRNA. The relative expression of genes was calculated using the 2-ΔΔCT method. The Primers used in the experiments were purchased from Sangon Biotech (Shanghai, China) Co., Ltd. and presented in Table S5.

### Plasmid construction and cell transfection

The construction of plasmids that overexpressed circMTO1 and ZIC1 (OE-ZIC1) were conducted by Geneseed Biotech (Guangzhou, China) Co., Ltd. Briefly. The full-length of human circMTO1 was amplified and inserted into the overexpression vector, pLC5-ciR. The ZIC1 expression plasmid was constructed by cloning the full-length ZIC1 open reading frame into the pcDNA3.1(+) vector. The sequence and orientation of the inserts were confirmed using DNA sequencing. The empty pcDNA3.1(+) vector was used as a negative control. For luciferase reporter plasmids, the circMTO1 sequences of wildtype and mutant type that bind to the miR-541-5p and the ZIC1 3′ UTR sequences of wildtype and mutant type that bind to the miR-541-5p were cloned into the pmirGLO vector (Promega, USA). Cell transfections were conducted with Lipofectamine 3000 (Invitrogen, USA) according to the manufacturer’s protocols.

### Small interfering RNA (siRNA), miRNA mimics, and inhibitor

The sequences of circMTO1 siRNAs (siRNA-1, siRNA-2, and siRNA-3 circMTO1), ZIC1 siRNAs (siRNA-1, siRNA-2, and siRNA-3 ZIC1), negative control (NC), miR-541-5p mimics, and miR-541-5p inhibitor were synthesized by GenePharma (Shanghai, China) and are presented in Table S6. CircMTO1 siRNA stable oligonucleotides (modified by cholesterol), cholesterol-conjugated 2′-OMe-modified, chemically modified single-stranded RNA molecule, miR-541-5p inhibitor, and cholesterol-conjugated NC in vivo experiment were synthesized by GenePharma (Shanghai, China).

### RNA pull-down assay

RNA pull-down experiments were performed with RNA Antisense Purification (RAP) Kit (BersinBio, China). Briefly, 4 × 10^7^ HepG2 and SMMC-7721 cells, overexpressed circMTO1, were collected, respectively. And the procedures: cross-link cells and collect cell pellet, homogenization, probe preparation (antisense oligo probes were designed using the following parameters: a. number of probes = 1 probe/100 nucleotides of RNA; b. target GC% = 45; c. oligonucleotide length = 20; d. spacing length = 60–80. circMTO1 probes: probe 1: TGACATCTGACCCAAAACAA; probe 2: GATGCGAGAACACAGGCCAT; probe 3: ATCAATCTGAGCTCTCAGAC; probe 4: AAGATCTTCTACAGCTCCCT. Negative probes: probe 1: CAAACGGCGGATTGACCGTAATGGGATAGGTCACGTTGGTGTAGATGGGCGCATCGTAAC;probe 2: CACCACATACAGGCCGTAGCGGTCGCACAGCGTGTACCACAGCGGATGGTTCGGATAATG;probe 3: CCAATCCGCGCCGGATGCGGTGTATCGCTCGCCACTTCAACATCAACGGTAATCGCCATT), beads preparation, hybridization and capture, RNA elution, RNA purification, were performed according to the manufacturer’s instructions. CircMTO1, linear MTO1, and miRNAs were detected using RT-qPCR.

### Dual-luciferase reporter assay

HEK293T cell was cultured and seeded into 24-well plates. The dual-luciferase reporter system was employed according to the manufacturer’s instructions. Briefly, circMTO1 and ZIC1 3′UTR and their mutant vectors were synthesized and subcloned into luciferase reporter vector pmirGLO, which were transfected into HEK293T cell combination with miR-541-5p mimics or NC mimics The relative luciferase activity was determined by the Dual-Luciferase^®^ Reporter 1000 Assay System (Promega, USA).

### CCK-8 assay

After 24 h transfection, cells were digested, and seeded into 96-well plates (4 × 10^3^/well). At 0, 24, 48, 72, and 96 h after seeding, each well was replaced with 100 μL fresh complete medium and 10 μL CCK-8 reagent (Dojindo, Japan) followed by incubation at 37 °C with 5% CO_2_ for 1 h. The absorbance of the solution was measured at 450 nm using a Multiskan FC detection system (Thermo Fisher, USA).

### Apoptosis assays

Briefly, cells were digested with EDTA-free trypsin and washed with ice-cold PBS. Apoptosis assays were executed using double staining with fluorescein APC-conjugated Annexin V and propidium iodide (PI). Next, the percentages of cells were analyzed on a flow cytometer (BD Accuri C6 Plus, USA). The software Flow Jo was used to analyze the data and draw the pictures.

### Transwell cell migration and invasion assays

The migration/invasion analysis was performed using Transwell plates without/with Matrigel gel in the top chambers. HepG2, SK-Hep1, and SMMC-7721 cells were transfected with NC, miR-541-5p mimics, miR-541-5p inhibitor, and/or si-circMTO1 and incubated in transwell plates for 24 h, digested with 0.25% trypsin, suspended in serum-free medium, and counted. Then, 200 μl of transfected cells were plated in the upper chamber, and 600 µl of DMEM containing 10% FBS was added to the lower chamber. After 24 h of incubation, the upper chamber was washed twice with PBS. The cells on the upper surface were discarded with a cotton swab and the cells left were fixed with anhydrous methanol for 30 min and stained with 0.1% crystal violet for 30 min. The cells that migrated through an 8-μM pored membrane or invaded through the Matrigel-coated membrane were stained and counted under a microscope.

### Western blot analysis

Cells and tissues were added with an appropriate amount of RIPA (PMSF) buffer. Then, the total protein was extracted by centrifugation, the total protein concentration was determined using the BCA method. Equal amounts of total protein (20 μg) were separated by 8% SDS-PAGE and transferred onto PVDF membranes (Millipore, Billerica, MA), followed by 5% skim milk for 1 h at room temperature to block nonspecific bindings. The membranes were incubated with the following primary antibodies overnight at 4 °C and washed five times with 1×TBST for 5 min each time: ZIC1 (Abcam, ab134951), β-catenin (Abcam, ab32572), c-myc (proteintech, 67447-1-lg), cyclin D1 (ab134175), E-cadherin (Abcam, ab40772), N-cadherin (ab76011), Vimentin (Abcam, ab92547), MMP2 (proteintech, 66366-1-lg). β-action (CWBIO, CW0096M) acted as the internal control. The membranes were then incubated with secondary antibodies (HRP-labeled goat anti-rabbit IgG or goat anti-mouse IgG) at room temperature for 1 h and washed five times with 1×TBST for 5 min each time. Bands were detected by a chemiluminescence imaging system (SageCreation Science, Beijing, China) with SuperSignal^TM^ West Femto Maximum Sensitivity Substrate (Thermo Scientific, USA).

### Xenograft tumorigenesis

25 SPF-grade male BALB/C nude mice (6–8 weeks) were purchased from Beijing Vital River Laboratory Animal Technology Co., Ltd. (Beijing, China) with an average weight of 15.4 g (ranging from 14.7 to 16.1 g). All nude mice were housed under SPF conditions (TECNIPLAST S.p.A., Italy) and randomly divided into five groups. A total of 5 × 10^6^ SMMC-7721 cells were suspended in 100 μL PBS and subcutaneously injected into the right flank of each mouse. Once the tumor volume reached 100–200 mm^3^, intratumoral injections of PBS, cholesterol-conjugated NC, cholesterol-conjugated si-circMTO1, cholesterol-conjugated 2′-OMe-modified miR-541-5p inhibitor, and cholesterol-conjugated si-circMTO1 + cholesterol-conjugated 2′-OMe-modified miR-541-5p inhibitor were administered a total of five times, three days apart. Tumor volume was calculated every three days, which was calculated as follows: V (volume) = (length × width^2^)/2. Tumor weight was weighted at the end-point of the experiment. After 24 days, the mice were killed and the tumors were dissected and processed for histological analysis.

### Immunohistochemistry (IHC) staining

In brief, paraffin sections of xenograft tumor tissues were cut into 4-μm sections. Sections underwent dewaxing, rehydration, antigen retrieval, and blocking, and then were incubated with antibodies against ZIC1, β-catenin, c-myc, cyclin D1, E-cadherin, N-cadherin, Vimentin, MMP2 for 70 min at room temperature in a moist chamber, and washed five times with PBS. Sections were incubated with HRP-conjugated secondary antibody for 20 min at room temperature, washed five times with PBS, and then stained with DAB and hematoxylin. Next, sections were dehydrated and mounted with coverslips.

### H&E staining

Tissue samples were fixed in 4% paraformaldehyde, embedded in paraffin, and cut into 4-μm thick sections. H&E staining was performed to observe the histopathological features.

### Statistical analysis

The Kolmogorov–Smirnov test was used to estimate the distribution of the data. Normally distributed continuous variables are presented as mean ± standard deviation values. Statistical significance was determined by a one-way ANOVA test or a Student’s *t*-test. Data were processed with GraphPad (San Diego, California, USA). All *P* values presented are two-tailed, and values of *P* < 0.05 were considered statistically significant. Analyses were performed using the SPSS 20.0 software (Armonk, NY, USA). The free online tool Venny 2.1 was used (https://bioinfogp.cnb.csic.es/tools/venny/).

## Supplementary information

Supplementary Table legends

Table S1

Table S2

Table S3

Table S4

Table S5

Table S6

author-contribution-form

aj-checklist

## Data Availability

The datasets used and analyzed during the current study are available from the corresponding author on reasonable request
