# Supplementary material for: CircMTO1 suppresses hepatocellular carcinoma progression via the miR-541-5p/ZIC1 axis by regulating Wnt/β-catenin signaling pathway and epithelial-to-mesenchymal transition
Source: Cell Death Dis. 2021 Dec 20;13(1):12. doi: 10.1038/s41419-021-04464-3 (PMC8688446; doi:10.1038/s41419-021-04464-3)
Supplement: Supplementary file 1 — Supplementary Table legends [file 41419_2021_4464_MOESM1_ESM.docx]

Table S1 The differentially expressed circRNAs from three microarrays (GSE97332, GSE94508, and GSE78520)

Table S2 The miRNAs may bind to circMTO1 predicted by ENCORI/starbase (http://starbase.sysu.edu.cn/), CSCD (http://gb.whu.edu.cn/CSCD/), circbank and miRanda databases.

Table S3 The possible miR-541-5p target genes predicted by miRDB, TargetScan and ENCORI/starbase databases.

Table S4 The 239 genes possible targeted by miR-541-5p predicted by all miRDB, TargetScan and ENCORI/starbase databases.

Table S5 Primers sequence.

Table S6 The information of the siRNAs, miRNA mimics, and inhibitor.
