# Supplementary material for: CircMTO1 suppresses hepatocellular carcinoma progression via the miR-541-5p/ZIC1 axis by regulating Wnt/β-catenin signaling pathway and epithelial-to-mesenchymal transition
Source: Cell Death Dis. 2021 Dec 20;13(1):12. doi: 10.1038/s41419-021-04464-3 (PMC8688446; doi:10.1038/s41419-021-04464-3)
Supplement: Supplementary file 2 — Table S1 [file 41419_2021_4464_MOESM2_ESM.docx]

Table S1 The differentially expressed circRNAs among three microarrays (GSE97332, GSE94508, and GSE78520)

| circRNAs | logFC | AveExpr | t | P.Value | adj.P.Val |
| --- | --- | --- | --- | --- | --- |
| Downregulated |  |  |  |  |  |
| hsa_circ_0052767 | -1.569 | 8.891 | -7.446 | 2.21E-08 | 4.46E-05 |
| hsa_circ_0011501 | -1.605 | 10.001 | -7.082 | 5.98E-08 | 5.92E-05 |
| hsa_circ_0001191 | -2.132 | 11.772 | -6.819 | 1.24E-07 | 5.92E-05 |
| hsa_circ_0003214 | -1.541 | 7.889 | -6.778 | 1.39E-07 | 5.92E-05 |
| hsa_circ_0049271 | -1.574 | 14.225 | -6.719 | 1.63E-07 | 5.92E-05 |
| hsa_circ_0000217 | -1.755 | 13.173 | -6.596 | 2.30E-07 | 5.92E-05 |
| hsa_circ_0004789 | -2.281 | 10.733 | -6.577 | 2.43E-07 | 5.92E-05 |
| hsa_circ_0009018 | -2.014 | 12.265 | -6.559 | 2.55E-07 | 5.92E-05 |
| hsa_circ_0000715 | -1.821 | 9.964 | -6.548 | 2.64E-07 | 5.92E-05 |
| hsa_circ_0079449 | -2.058 | 11.978 | -6.489 | 3.11E-07 | 6.27E-05 |
| hsa_circ_0008667 | -1.726 | 8.077 | -6.448 | 3.49E-07 | 6.40E-05 |
| hsa_circ_0023704 | -2.241 | 10.764 | -6.343 | 4.69E-07 | 7.29E-05 |
| hsa_circ_0000613 | -1.820 | 7.856 | -6.086 | 9.69E-07 | 0.000121362 |
| hsa_circ_0008797 | -1.573 | 14.293 | -6.083 | 9.78E-07 | 0.000121362 |
| hsa_circ_0008153 | -1.868 | 7.819 | -6.036 | 1.12E-06 | 0.000121362 |
| hsa_circ_0005139 | -1.752 | 13.498 | -6.023 | 1.16E-06 | 0.000121362 |
| hsa_circ_0000220 | -1.969 | 9.851 | -5.942 | 1.46E-06 | 0.000140316 |
| hsa_circ_0056285 | -1.417 | 12.057 | -5.885 | 1.72E-06 | 0.00015067 |
| hsa_circ_0026337 | -1.316 | 14.101 | -5.799 | 2.19E-06 | 0.000177443 |
| hsa_circ_0023685 | -1.537 | 11.102 | -5.794 | 2.22E-06 | 0.000177443 |
| hsa_circ_0063329 | -1.579 | 8.389 | -5.774 | 2.36E-06 | 0.000177443 |
| hsa_circ_0058051 | -2.144 | 10.876 | -5.756 | 2.48E-06 | 0.000177443 |
| hsa_circ_0001167 | -1.556 | 13.926 | -5.750 | 2.52E-06 | 0.000177443 |
| hsa_circ_0001712 | -1.881 | 11.559 | -5.696 | 2.95E-06 | 0.000196339 |
| hsa_circ_0005394 | -2.179 | 13.232 | -5.655 | 3.31E-06 | 0.000196339 |
| hsa_circ_0029596 | -2.995 | 10.437 | -5.640 | 3.45E-06 | 0.000196339 |
| hsa_circ_0000219 | -1.790 | 9.756 | -5.639 | 3.46E-06 | 0.000196339 |
| hsa_circ_0058055 | -1.943 | 10.918 | -5.636 | 3.49E-06 | 0.000196339 |
| hsa_circ_0076798 | -2.224 | 12.948 | -5.635 | 3.50E-06 | 0.000196339 |
| hsa_circ_0000978 | -2.051 | 10.145 | -5.624 | 3.62E-06 | 0.000196602 |
| hsa_circ_0055412 | -2.120 | 13.184 | -5.574 | 4.17E-06 | 0.000210496 |
| hsa_circ_0007178 | -1.892 | 10.019 | -5.512 | 4.98E-06 | 0.0002431 |
| hsa_circ_0004033 | -2.464 | 12.331 | -5.507 | 5.05E-06 | 0.0002431 |
| hsa_circ_0005480 | -1.728 | 13.841 | -5.453 | 5.90E-06 | 0.000270024 |
| hsa_circ_0028048 | -1.547 | 11.986 | -5.440 | 6.12E-06 | 0.000270024 |
| hsa_circ_0032683 | -3.440 | 12.148 | -5.438 | 6.15E-06 | 0.000270024 |
| hsa_circ_0043256 | -1.655 | 11.783 | -5.425 | 6.39E-06 | 0.000274548 |
| hsa_circ_0043947 | -1.842 | 13.770 | -5.358 | 7.74E-06 | 0.000308608 |
| hsa_circ_0063158 | -2.117 | 13.342 | -5.356 | 7.79E-06 | 0.000308608 |
| hsa_circ_0003497 | -1.603 | 13.844 | -5.303 | 9.06E-06 | 0.000345205 |
| hsa_circ_0006078 | -1.469 | 13.065 | -5.278 | 9.72E-06 | 0.000354456 |
| hsa_circ_0002383 | -1.453 | 12.922 | -5.277 | 9.77E-06 | 0.000354456 |
| hsa_circ_0003285 | -1.164 | 12.689 | -5.259 | 1.03E-05 | 0.000360433 |
| hsa_circ_0056856 | -2.254 | 13.382 | -5.257 | 1.03E-05 | 0.000360433 |
| hsa_circ_0078610 | -1.924 | 9.771 | -5.222 | 1.14E-05 | 0.000374818 |
| hsa_circ_0002877 | -1.528 | 8.949 | -5.215 | 1.17E-05 | 0.000374818 |
| hsa_circ_0001605 | -1.199 | 13.815 | -5.211 | 1.18E-05 | 0.000374818 |
| hsa_circ_0004441 | -2.395 | 10.713 | -5.208 | 1.19E-05 | 0.000374818 |
| hsa_circ_0003056 | -2.498 | 10.407 | -5.193 | 1.24E-05 | 0.000374818 |
| hsa_circ_0057319 | -1.695 | 9.031 | -5.187 | 1.26E-05 | 0.000374818 |
| hsa_circ_0032664 | -3.865 | 11.243 | -5.182 | 1.28E-05 | 0.000374818 |
| hsa_circ_0000379 | -2.213 | 13.361 | -5.177 | 1.30E-05 | 0.000375497 |
| hsa_circ_0004069 | -1.845 | 11.816 | -5.164 | 1.35E-05 | 0.000383803 |
| hsa_circ_0001887 | -1.656 | 13.763 | -5.156 | 1.38E-05 | 0.000384744 |
| hsa_circ_0030569 | -2.202 | 13.116 | -5.154 | 1.39E-05 | 0.000384744 |
| hsa_circ_0000086 | -1.632 | 12.141 | -5.136 | 1.46E-05 | 0.000394353 |
| hsa_circ_0043785 | -2.280 | 9.569 | -5.059 | 1.82E-05 | 0.000462964 |
| hsa_circ_0041555 | -1.816 | 14.276 | -5.057 | 1.84E-05 | 0.000462964 |
| hsa_circ_0006665 | -1.742 | 9.580 | -5.052 | 1.86E-05 | 0.000462964 |
| hsa_circ_0003239 | -1.984 | 13.310 | -5.019 | 2.04E-05 | 0.00047963 |
| hsa_circ_0008861 | -1.052 | 9.511 | -5.010 | 2.10E-05 | 0.000481256 |
| hsa_circ_0007934 | -1.807 | 11.028 | -5.010 | 2.10E-05 | 0.000481256 |
| hsa_circ_0002807 | -1.519 | 12.131 | -5.006 | 2.12E-05 | 0.000481256 |
| hsa_circ_0004217 | -1.471 | 13.301 | -4.957 | 2.44E-05 | 0.000538381 |
| hsa_circ_0004207 | -2.936 | 12.180 | -4.941 | 2.56E-05 | 0.000538381 |
| hsa_circ_0005916 | -2.616 | 9.195 | -4.937 | 2.58E-05 | 0.000538381 |
| hsa_circ_0070659 | -2.472 | 13.026 | -4.929 | 2.65E-05 | 0.000538381 |
| hsa_circ_0008139 | -1.625 | 8.636 | -4.926 | 2.67E-05 | 0.000538381 |
| hsa_circ_0000508 | -2.189 | 12.813 | -4.919 | 2.72E-05 | 0.000544707 |
| hsa_circ_0069249 | -1.884 | 12.992 | -4.904 | 2.84E-05 | 0.000556894 |
| hsa_circ_0040573 | -1.571 | 10.687 | -4.873 | 3.10E-05 | 0.000602351 |
| hsa_circ_0035559 | -1.914 | 9.418 | -4.860 | 3.22E-05 | 0.00061432 |
| hsa_circ_0000045 | -2.288 | 10.736 | -4.844 | 3.38E-05 | 0.000637208 |
| hsa_circ_0031017 | -1.371 | 12.033 | -4.836 | 3.45E-05 | 0.000641961 |
| hsa_circ_0005029 | -1.462 | 8.920 | -4.763 | 4.26E-05 | 0.000747769 |
| hsa_circ_0058050 | -1.088 | 6.617 | -4.754 | 4.36E-05 | 0.000759292 |
| hsa_circ_0005651 | -1.856 | 9.428 | -4.751 | 4.40E-05 | 0.000760029 |
| hsa_circ_0069570 | -3.119 | 10.744 | -4.746 | 4.46E-05 | 0.000764024 |
| hsa_circ_0008856 | -1.156 | 11.355 | -4.732 | 4.64E-05 | 0.000782858 |
| hsa_circ_0000662 | -1.232 | 14.543 | -4.729 | 4.69E-05 | 0.000782858 |
| hsa_circ_0047821 | -2.142 | 10.831 | -4.727 | 4.71E-05 | 0.000782858 |
| hsa_circ_0067997 | -2.254 | 8.367 | -4.726 | 4.73E-05 | 0.000782858 |
| hsa_circ_0036282 | -1.594 | 10.886 | -4.696 | 5.15E-05 | 0.000832834 |
| hsa_circ_0003045 | -2.783 | 11.054 | -4.687 | 5.27E-05 | 0.00084553 |
| hsa_circ_0002490 | -2.541 | 11.341 | -4.682 | 5.36E-05 | 0.000846041 |
| hsa_circ_0000189 | -1.714 | 11.552 | -4.663 | 5.66E-05 | 0.000859212 |
| hsa_circ_0001516 | -1.271 | 8.426 | -4.655 | 5.78E-05 | 0.00086178 |
| hsa_circ_0004240 | -1.517 | 11.889 | -4.654 | 5.80E-05 | 0.00086178 |
| hsa_circ_0062317 | -2.269 | 10.644 | -4.633 | 6.16E-05 | 0.000894538 |
| hsa_circ_0040534 | -1.285 | 10.638 | -4.618 | 6.43E-05 | 0.000908708 |
| hsa_circ_0005252 | -1.774 | 12.293 | -4.603 | 6.71E-05 | 0.000941789 |
| hsa_circ_0068641 | -1.497 | 11.430 | -4.597 | 6.83E-05 | 0.000950834 |
| hsa_circ_0000507 | -1.552 | 11.647 | -4.594 | 6.88E-05 | 0.000951715 |
| hsa_circ_0035554 | -1.459 | 11.566 | -4.589 | 6.99E-05 | 0.000957018 |
| hsa_circ_0007518 | -1.564 | 12.926 | -4.553 | 7.74E-05 | 0.001032866 |
| hsa_circ_0047744 | -1.914 | 12.570 | -4.523 | 8.42E-05 | 0.001089952 |
| hsa_circ_0006857 | -1.461 | 14.270 | -4.498 | 9.05E-05 | 0.001149725 |
| hsa_circ_0078607 | -1.666 | 9.253 | -4.487 | 9.32E-05 | 0.001176846 |
| hsa_circ_0029633 | -1.402 | 9.799 | -4.465 | 9.92E-05 | 0.001219858 |
| hsa_circ_0005079 | -1.386 | 7.674 | -4.452 | 0.000103 | 0.001219858 |
| hsa_circ_0000958 | -1.547 | 11.969 | -4.434 | 0.000108 | 0.001236918 |
| hsa_circ_0031851 | -2.465 | 9.782 | -4.411 | 0.000116 | 0.001296426 |
| hsa_circ_0000661 | -1.172 | 8.796 | -4.401 | 0.000119 | 0.001314896 |
| hsa_circ_0001288 | -1.854 | 10.898 | -4.374 | 0.000128 | 0.001396499 |
| hsa_circ_0001033 | -1.402 | 11.573 | -4.370 | 0.00013 | 0.001396499 |
| hsa_circ_0067013 | -1.263 | 11.532 | -4.370 | 0.00013 | 0.001396499 |
| hsa_circ_0007765 | -1.316 | 7.636 | -4.318 | 0.00015 | 0.001532556 |
| hsa_circ_0041252 | -1.904 | 9.928 | -4.305 | 0.000156 | 0.001560644 |
| hsa_circ_0017348 | -2.256 | 8.903 | -4.280 | 0.000167 | 0.001618079 |
| hsa_circ_0004913 | -2.978 | 7.842 | -4.250 | 0.000182 | 0.001703006 |
| hsa_circ_0048129 | -1.842 | 10.392 | -4.239 | 0.000188 | 0.001721962 |
| hsa_circ_0008354 | -1.451 | 9.790 | -4.230 | 0.000193 | 0.00174514 |
| hsa_circ_0047288 | -2.108 | 11.610 | -4.228 | 0.000194 | 0.001748617 |
| hsa_circ_0071834 | -1.485 | 8.833 | -4.204 | 0.000207 | 0.001836245 |
| hsa_circ_0003273 | -1.188 | 7.352 | -4.202 | 0.000208 | 0.001836245 |
| hsa_circ_0007591 | -1.573 | 7.238 | -4.180 | 0.000222 | 0.001922338 |
| hsa_circ_0087232 | -1.742 | 7.415 | -4.179 | 0.000223 | 0.001922338 |
| hsa_circ_0000550 | -1.582 | 10.339 | -4.173 | 0.000226 | 0.001937418 |
| hsa_circ_0043244 | -2.096 | 10.586 | -4.153 | 0.000239 | 0.001987317 |
| hsa_circ_0030045 | -1.089 | 11.175 | -4.104 | 0.000274 | 0.002196389 |
| hsa_circ_0079385 | -1.478 | 14.042 | -4.093 | 0.000283 | 0.002229437 |
| hsa_circ_0000009 | -1.783 | 11.159 | -4.067 | 0.000304 | 0.002318057 |
| hsa_circ_0051527 | -2.435 | 10.432 | -4.060 | 0.00031 | 0.002353339 |
| hsa_circ_0000333 | -2.327 | 10.549 | -4.050 | 0.000318 | 0.002399542 |
| hsa_circ_0062397 | -1.246 | 7.840 | -4.049 | 0.00032 | 0.002401071 |
| hsa_circ_0001874 | -1.133 | 9.866 | -4.045 | 0.000323 | 0.002417071 |
| hsa_circ_0003952 | -1.778 | 8.807 | -4.019 | 0.000347 | 0.002568402 |
| hsa_circ_0087354 | -1.330 | 10.968 | -4.018 | 0.000348 | 0.002568402 |
| hsa_circ_0014437 | -1.204 | 8.785 | -4.013 | 0.000353 | 0.002581103 |
| hsa_circ_0041871 | -2.441 | 9.416 | -4.012 | 0.000354 | 0.002581103 |
| hsa_circ_0008351 | -1.904 | 7.845 | -4.007 | 0.000359 | 0.002601979 |
| hsa_circ_0056472 | -2.735 | 10.370 | -4.007 | 0.000359 | 0.002601979 |
| hsa_circ_0008682 | -1.105 | 8.597 | -4.002 | 0.000364 | 0.002615738 |
| hsa_circ_0087429 | -1.502 | 9.023 | -3.955 | 0.000415 | 0.002910561 |
| hsa_circ_0005039 | -3.657 | 10.150 | -3.951 | 0.00042 | 0.002933963 |
| hsa_circ_0008035 | -1.877 | 6.673 | -3.945 | 0.000427 | 0.002970144 |
| hsa_circ_0065220 | -1.119 | 7.475 | -3.937 | 0.000436 | 0.002993689 |
| hsa_circ_0072387 | -1.331 | 7.135 | -3.936 | 0.000437 | 0.002993689 |
| hsa_circ_0002955 | -1.024 | 6.978 | -3.929 | 0.000445 | 0.00303998 |
| hsa_circ_0000999 | -1.522 | 10.499 | -3.923 | 0.000453 | 0.003071449 |
| hsa_circ_0000670 | -1.567 | 14.424 | -3.883 | 0.000506 | 0.003345776 |
| hsa_circ_0043302 | -1.152 | 6.826 | -3.880 | 0.00051 | 0.003345776 |
| hsa_circ_0008160 | -1.288 | 8.115 | -3.879 | 0.000512 | 0.003346105 |
| hsa_circ_0001579 | -1.240 | 6.848 | -3.866 | 0.000531 | 0.003416902 |
| hsa_circ_0019172 | -1.271 | 9.807 | -3.859 | 0.000541 | 0.003438957 |
| hsa_circ_0062545 | -1.109 | 7.293 | -3.859 | 0.000541 | 0.003438957 |
| hsa_circ_0009715 | -2.763 | 10.755 | -3.839 | 0.000572 | 0.003562756 |
| hsa_circ_0001213 | -1.196 | 7.728 | -3.829 | 0.000588 | 0.003643769 |
| hsa_circ_0000249 | -1.231 | 8.983 | -3.818 | 0.000605 | 0.00372546 |
| hsa_circ_0007387 | -2.565 | 10.758 | -3.816 | 0.000609 | 0.00372546 |
| hsa_circ_0069559 | -1.160 | 11.814 | -3.809 | 0.00062 | 0.003772737 |
| hsa_circ_0046565 | -1.510 | 10.002 | -3.797 | 0.000642 | 0.003858993 |
| hsa_circ_0002881 | -1.685 | 11.453 | -3.782 | 0.000669 | 0.003938913 |
| hsa_circ_0007961 | -2.523 | 8.715 | -3.781 | 0.000671 | 0.003938913 |
| hsa_circ_0008144 | -1.446 | 9.611 | -3.763 | 0.000704 | 0.004096853 |
| hsa_circ_0025006 | -1.864 | 8.952 | -3.746 | 0.000738 | 0.004247916 |
| hsa_circ_0047303 | -1.085 | 9.476 | -3.728 | 0.000775 | 0.004409282 |
| hsa_circ_0009020 | -2.604 | 10.613 | -3.715 | 0.000804 | 0.004488573 |
| hsa_circ_0000266 | -2.310 | 9.395 | -3.715 | 0.000804 | 0.004488573 |
| hsa_circ_0005245 | -1.476 | 10.730 | -3.714 | 0.000804 | 0.004488573 |
| hsa_circ_0019612 | -1.166 | 7.600 | -3.649 | 0.000962 | 0.00512529 |
| hsa_circ_0005711 | -1.151 | 10.204 | -3.632 | 0.001006 | 0.00533433 |
| hsa_circ_0006988 | -2.675 | 9.002 | -3.584 | 0.001144 | 0.005882023 |
| hsa_circ_0012673 | -1.697 | 9.551 | -3.565 | 0.001207 | 0.006110007 |
| hsa_circ_0007066 | -1.874 | 8.980 | -3.561 | 0.001217 | 0.006148001 |
| hsa_circ_0000591 | -1.533 | 7.842 | -3.548 | 0.001261 | 0.006352536 |
| hsa_circ_0083866 | -1.737 | 7.886 | -3.545 | 0.001274 | 0.006368137 |
| hsa_circ_0011264 | -1.209 | 7.834 | -3.524 | 0.001346 | 0.006650344 |
| hsa_circ_0007701 | -1.536 | 10.708 | -3.503 | 0.001425 | 0.006917345 |
| hsa_circ_0000325 | -1.759 | 7.400 | -3.483 | 0.001505 | 0.007219653 |
| hsa_circ_0050532 | -1.169 | 7.799 | -3.475 | 0.001535 | 0.007347728 |
| hsa_circ_0077892 | -1.703 | 9.277 | -3.459 | 0.001603 | 0.007603436 |
| hsa_circ_0023409 | -1.262 | 9.567 | -3.453 | 0.001626 | 0.007649435 |
| hsa_circ_0047285 | -2.387 | 9.551 | -3.448 | 0.00165 | 0.007697108 |
| hsa_circ_0032641 | -1.541 | 7.707 | -3.442 | 0.001677 | 0.00774999 |
| hsa_circ_0007874 | -1.111 | 13.127 | -3.439 | 0.00169 | 0.007752719 |
| hsa_circ_0005378 | -1.104 | 6.416 | -3.435 | 0.001707 | 0.007777701 |
| hsa_circ_0008838 | -1.304 | 7.152 | -3.435 | 0.00171 | 0.007777701 |
| hsa_circ_0077520 | -1.214 | 8.085 | -3.432 | 0.001721 | 0.007813134 |
| hsa_circ_0007845 | -1.234 | 6.786 | -3.424 | 0.001757 | 0.007939383 |
| hsa_circ_0077179 | -1.217 | 8.698 | -3.417 | 0.00179 | 0.008062018 |
| hsa_circ_0000699 | -2.894 | 9.026 | -3.411 | 0.00182 | 0.008098368 |
| hsa_circ_0025633 | -2.877 | 9.727 | -3.394 | 0.001907 | 0.008330811 |
| hsa_circ_0071023 | -2.583 | 8.113 | -3.393 | 0.001909 | 0.008330811 |
| hsa_circ_0052130 | -2.571 | 9.759 | -3.390 | 0.001927 | 0.008391146 |
| hsa_circ_0004771 | -1.536 | 7.580 | -3.327 | 0.002274 | 0.009641498 |
| hsa_circ_0068606 | -1.100 | 12.351 | -3.284 | 0.002547 | 0.01043773 |
| hsa_circ_0000034 | -1.708 | 10.715 | -3.278 | 0.002587 | 0.010556133 |
| hsa_circ_0007762 | -1.237 | 7.830 | -3.263 | 0.002686 | 0.010875185 |
| hsa_circ_0008847 | -1.134 | 7.101 | -3.255 | 0.002744 | 0.011052822 |
| hsa_circ_0054254 | -1.794 | 9.098 | -3.239 | 0.002866 | 0.011371984 |
| hsa_circ_0001581 | -2.199 | 8.631 | -3.221 | 0.002998 | 0.01176035 |
| hsa_circ_0002690 | -2.345 | 8.374 | -3.215 | 0.003045 | 0.011898486 |
| hsa_circ_0045602 | -1.205 | 8.568 | -3.207 | 0.003111 | 0.012083388 |
| hsa_circ_0008832 | -1.396 | 9.396 | -3.201 | 0.003164 | 0.01217719 |
| hsa_circ_0035277 | -2.008 | 9.785 | -3.195 | 0.003212 | 0.012285406 |
| hsa_circ_0008661 | -1.388 | 8.679 | -3.176 | 0.003377 | 0.012642435 |
| hsa_circ_0040000 | -1.117 | 7.214 | -3.157 | 0.003545 | 0.013065902 |
| hsa_circ_0008762 | -2.407 | 8.567 | -3.145 | 0.003658 | 0.013339245 |
| hsa_circ_0066351 | -1.569 | 8.433 | -3.142 | 0.00368 | 0.013395366 |
| hsa_circ_0031677 | -1.553 | 8.178 | -3.129 | 0.003805 | 0.013749211 |
| hsa_circ_0004958 | -1.412 | 8.234 | -3.128 | 0.003818 | 0.013772579 |
| hsa_circ_0002286 | -1.981 | 7.376 | -3.126 | 0.003833 | 0.013802921 |
| hsa_circ_0057684 | -1.019 | 7.885 | -3.088 | 0.004229 | 0.014960777 |
| hsa_circ_0082413 | -2.424 | 8.145 | -3.074 | 0.004381 | 0.015454567 |
| hsa_circ_0000679 | -1.631 | 9.326 | -3.074 | 0.004384 | 0.015454567 |
| hsa_circ_0001573 | -3.506 | 8.871 | -3.066 | 0.004473 | 0.015659596 |
| hsa_circ_0036398 | -1.763 | 7.586 | -3.060 | 0.004544 | 0.015826133 |
| hsa_circ_0000540 | -1.695 | 9.343 | -3.046 | 0.004716 | 0.016227365 |
| hsa_circ_0005243 | -2.390 | 9.317 | -3.025 | 0.004975 | 0.016973837 |
| hsa_circ_0001190 | -1.484 | 7.577 | -3.023 | 0.004999 | 0.016999797 |
| hsa_circ_0078383 | -1.233 | 8.808 | -3.021 | 0.005015 | 0.017026311 |
| hsa_circ_0059512 | -1.119 | 7.775 | -3.020 | 0.005034 | 0.017060934 |
| hsa_circ_0001556 | -2.078 | 7.776 | -3.002 | 0.005263 | 0.017529846 |
| hsa_circ_0072263 | -1.948 | 8.540 | -2.994 | 0.005373 | 0.017764184 |
| hsa_circ_0008432 | -1.860 | 7.752 | -2.960 | 0.005857 | 0.01892825 |
| hsa_circ_0007113 | -1.000 | 8.334 | -2.943 | 0.006113 | 0.019677958 |
| hsa_circ_0063266 | -2.221 | 8.727 | -2.927 | 0.006358 | 0.020322922 |
| hsa_circ_0022723 | -1.297 | 7.570 | -2.922 | 0.006446 | 0.020480141 |
| hsa_circ_0000994 | -1.025 | 7.372 | -2.917 | 0.006519 | 0.020622009 |
| hsa_circ_0005051 | -1.064 | 7.016 | -2.906 | 0.006717 | 0.021036007 |
| hsa_circ_0079064 | -1.618 | 7.329 | -2.890 | 0.006991 | 0.021825123 |
| hsa_circ_0028132 | -2.314 | 9.026 | -2.883 | 0.007108 | 0.022089328 |
| hsa_circ_0000374 | -2.843 | 8.724 | -2.857 | 0.007582 | 0.023170832 |
| hsa_circ_0024758 | -2.877 | 8.949 | -2.856 | 0.007606 | 0.023208549 |
| hsa_circ_0000068 | -1.522 | 8.276 | -2.848 | 0.007748 | 0.023499875 |
| hsa_circ_0000765 | -1.451 | 9.024 | -2.836 | 0.00799 | 0.024197511 |
| hsa_circ_0077096 | -1.544 | 8.431 | -2.821 | 0.008293 | 0.024928458 |
| hsa_circ_0003437 | -1.925 | 7.435 | -2.818 | 0.008338 | 0.024985366 |
| hsa_circ_0004368 | -1.893 | 11.495 | -2.818 | 0.008358 | 0.024985366 |
| hsa_circ_0005387 | -2.136 | 8.309 | -2.812 | 0.008473 | 0.025242888 |
| hsa_circ_0004503 | -1.174 | 7.005 | -2.802 | 0.008676 | 0.025602445 |
| hsa_circ_0006332 | -1.524 | 8.592 | -2.774 | 0.009305 | 0.027123682 |
| hsa_circ_0063306 | -2.339 | 7.935 | -2.769 | 0.00942 | 0.02733652 |
| hsa_circ_0075393 | -3.121 | 8.320 | -2.768 | 0.009432 | 0.02733652 |
| hsa_circ_0038899 | -1.680 | 7.615 | -2.765 | 0.009511 | 0.027524548 |
| hsa_circ_0000370 | -1.455 | 9.713 | -2.757 | 0.009695 | 0.027896889 |
| hsa_circ_0000040 | -2.008 | 8.507 | -2.755 | 0.009747 | 0.027988767 |
| hsa_circ_0005053 | -1.007 | 7.864 | -2.755 | 0.009755 | 0.027988767 |
| hsa_circ_0002259 | -1.856 | 7.445 | -2.753 | 0.009783 | 0.028019196 |
| hsa_circ_0000254 | -1.593 | 10.944 | -2.733 | 0.010286 | 0.029207588 |
| hsa_circ_0067301 | -1.108 | 8.512 | -2.720 | 0.010608 | 0.029844855 |
| hsa_circ_0046534 | -1.842 | 8.276 | -2.711 | 0.01084 | 0.030411598 |
| hsa_circ_0008928 | -1.311 | 6.971 | -2.707 | 0.010962 | 0.030711946 |
| hsa_circ_0003340 | -1.622 | 7.004 | -2.703 | 0.011048 | 0.030868048 |
| hsa_circ_0036610 | -1.318 | 7.349 | -2.700 | 0.01115 | 0.031023065 |
| hsa_circ_0073736 | -1.050 | 11.447 | -2.674 | 0.01186 | 0.032729438 |
| hsa_circ_0044603 | -1.607 | 11.969 | -2.671 | 0.011937 | 0.032806002 |
| hsa_circ_0008614 | -1.440 | 8.003 | -2.669 | 0.012019 | 0.032987886 |
| hsa_circ_0001539 | -1.571 | 7.594 | -2.663 | 0.012174 | 0.033366896 |
| hsa_circ_0045888 | -1.010 | 7.674 | -2.649 | 0.012597 | 0.034202308 |
| hsa_circ_0007763 | -1.017 | 8.778 | -2.647 | 0.012666 | 0.034297878 |
| hsa_circ_0001614 | -1.715 | 8.226 | -2.624 | 0.01339 | 0.035778047 |
| hsa_circ_0004382 | -1.798 | 8.519 | -2.613 | 0.013721 | 0.036469382 |
| hsa_circ_0005267 | -2.491 | 8.672 | -2.613 | 0.013747 | 0.036490347 |
| hsa_circ_0005562 | -1.006 | 11.868 | -2.611 | 0.013783 | 0.03653808 |
| hsa_circ_0000036 | -2.153 | 9.414 | -2.604 | 0.014018 | 0.036851118 |
| hsa_circ_0006980 | -1.107 | 8.381 | -2.603 | 0.014056 | 0.036873073 |
| hsa_circ_0006374 | -1.488 | 7.911 | -2.602 | 0.014107 | 0.036960092 |
| hsa_circ_0005955 | -1.071 | 6.985 | -2.587 | 0.014599 | 0.037904071 |
| hsa_circ_0008015 | -2.560 | 8.145 | -2.514 | 0.017368 | 0.04344467 |
| hsa_circ_0008529 | -1.044 | 10.309 | -2.512 | 0.017421 | 0.04344467 |
| hsa_circ_0002300 | -2.020 | 8.921 | -2.472 | 0.019142 | 0.046699945 |
| hsa_circ_0060828 | -1.748 | 8.224 | -2.471 | 0.019202 | 0.046788444 |
| hsa_circ_0001853 | -2.697 | 9.524 | -2.447 | 0.020284 | 0.048722586 |
| Upregulated |  |  |  |  |  |
| hsa_circ_0041829 | 1.634 | 12.104 | 6.374 | 4.29E-07 | 7.23E-05 |
| hsa_circ_0006735 | 1.444 | 10.138 | 6.021 | 1.17E-06 | 0.000121 |
| hsa_circ_0022505 | 1.312 | 8.797 | 6.010 | 1.20E-06 | 0.000121 |
| hsa_circ_0007001 | 1.394 | 9.110 | 5.923 | 1.54E-06 | 0.000141 |
| hsa_circ_0068176 | 1.858 | 9.202 | 5.652 | 3.34E-06 | 0.000196 |
| hsa_circ_0006553 | 1.663 | 12.896 | 5.608 | 3.79E-06 | 0.000197 |
| hsa_circ_0008226 | 2.373 | 8.978 | 5.440 | 6.12E-06 | 0.00027 |
| hsa_circ_0016867 | 3.127 | 8.876 | 5.405 | 6.76E-06 | 0.000285 |
| hsa_circ_0042521 | 1.330 | 8.512 | 5.371 | 7.46E-06 | 0.000307 |
| hsa_circ_0008946 | 1.304 | 9.127 | 5.275 | 9.83E-06 | 0.000354 |
| hsa_circ_0013062 | 2.311 | 8.256 | 5.215 | 1.17E-05 | 0.000375 |
| hsa_circ_0079958 | 1.212 | 8.836 | 5.210 | 1.18E-05 | 0.000375 |
| hsa_circ_0000956 | 1.095 | 8.117 | 5.117 | 1.55E-05 | 0.000411 |
| hsa_circ_0019627 | 1.723 | 7.391 | 5.051 | 1.86E-05 | 0.000463 |
| hsa_circ_0008016 | 1.360 | 12.733 | 5.046 | 1.89E-05 | 0.000463 |
| hsa_circ_0030793 | 1.872 | 10.739 | 5.037 | 1.94E-05 | 0.000468 |
| hsa_circ_0041992 | 1.174 | 13.583 | 5.022 | 2.03E-05 | 0.00048 |
| hsa_circ_0007146 | 2.521 | 11.139 | 4.943 | 2.54E-05 | 0.000538 |
| hsa_circ_0004366 | 1.065 | 8.773 | 4.940 | 2.56E-05 | 0.000538 |
| hsa_circ_0060063 | 2.089 | 8.730 | 4.938 | 2.58E-05 | 0.000538 |
| hsa_circ_0002078 | 1.859 | 8.566 | 4.936 | 2.59E-05 | 0.000538 |
| hsa_circ_0017639 | 2.614 | 10.138 | 4.869 | 3.14E-05 | 0.000605 |
| hsa_circ_0002945 | 2.137 | 10.197 | 4.835 | 3.46E-05 | 0.000642 |
| hsa_circ_0072758 | 1.971 | 8.203 | 4.831 | 3.50E-05 | 0.000643 |
| hsa_circ_0006014 | 1.330 | 7.795 | 4.816 | 3.66E-05 | 0.000665 |
| hsa_circ_0003006 | 2.527 | 10.764 | 4.794 | 3.89E-05 | 0.000695 |
| hsa_circ_0015928 | 1.401 | 9.168 | 4.776 | 4.10E-05 | 0.000726 |
| hsa_circ_0000053 | 1.297 | 7.437 | 4.717 | 4.85E-05 | 0.000797 |
| hsa_circ_0002320 | 1.502 | 9.266 | 4.683 | 5.34E-05 | 0.000846 |
| hsa_circ_0001998 | 1.054 | 10.846 | 4.663 | 5.65E-05 | 0.000859 |
| hsa_circ_0078522 | 2.548 | 9.585 | 4.663 | 5.66E-05 | 0.000859 |
| hsa_circ_0068669 | 1.014 | 7.410 | 4.640 | 6.04E-05 | 0.000891 |
| hsa_circ_0039914 | 1.121 | 7.163 | 4.635 | 6.13E-05 | 0.000895 |
| hsa_circ_0001338 | 1.023 | 12.281 | 4.625 | 6.29E-05 | 0.000908 |
| hsa_circ_0076054 | 1.492 | 8.439 | 4.618 | 6.43E-05 | 0.000909 |
| hsa_circ_0007769 | 2.074 | 10.251 | 4.549 | 7.82E-05 | 0.001033 |
| hsa_circ_0005835 | 1.995 | 8.147 | 4.546 | 7.89E-05 | 0.001035 |
| hsa_circ_0001749 | 1.427 | 8.330 | 4.531 | 8.22E-05 | 0.001071 |
| hsa_circ_0004071 | 1.714 | 9.447 | 4.482 | 9.47E-05 | 0.001188 |
| hsa_circ_0004599 | 1.729 | 11.469 | 4.474 | 9.67E-05 | 0.001206 |
| hsa_circ_0052455 | 1.956 | 8.381 | 4.462 | 0.0001 | 0.00122 |
| hsa_circ_0046123 | 3.117 | 11.870 | 4.455 | 0.000102 | 0.00122 |
| hsa_circ_0044185 | 2.023 | 7.999 | 4.453 | 0.000103 | 0.00122 |
| hsa_circ_0008616 | 1.719 | 11.887 | 4.449 | 0.000104 | 0.00122 |
| hsa_circ_0010486 | 1.217 | 12.289 | 4.447 | 0.000104 | 0.00122 |
| hsa_circ_0058753 | 1.103 | 8.029 | 4.440 | 0.000106 | 0.001229 |
| hsa_circ_0003220 | 2.358 | 9.152 | 4.436 | 0.000108 | 0.001237 |
| hsa_circ_0009065 | 1.924 | 8.904 | 4.427 | 0.000111 | 0.001256 |
| hsa_circ_0005188 | 2.430 | 8.669 | 4.401 | 0.000119 | 0.001315 |
| hsa_circ_0048234 | 1.209 | 8.321 | 4.387 | 0.000124 | 0.00136 |
| hsa_circ_0000877 | 2.841 | 11.910 | 4.334 | 0.000144 | 0.001523 |
| hsa_circ_0008768 | 1.236 | 8.597 | 4.330 | 0.000146 | 0.001523 |
| hsa_circ_0059175 | 2.023 | 8.650 | 4.330 | 0.000146 | 0.001523 |
| hsa_circ_0008758 | 1.559 | 7.776 | 4.328 | 0.000146 | 0.001523 |
| hsa_circ_0009004 | 1.093 | 8.646 | 4.321 | 0.000149 | 0.001533 |
| hsa_circ_0074736 | 1.005 | 13.130 | 4.320 | 0.00015 | 0.001533 |
| hsa_circ_0070934 | 2.030 | 8.182 | 4.320 | 0.00015 | 0.001533 |
| hsa_circ_0023696 | 1.065 | 7.853 | 4.315 | 0.000152 | 0.001539 |
| hsa_circ_0048122 | 2.257 | 9.333 | 4.313 | 0.000152 | 0.00154 |
| hsa_circ_0007713 | 1.094 | 9.983 | 4.308 | 0.000155 | 0.001557 |
| hsa_circ_0066147 | 2.336 | 12.500 | 4.289 | 0.000163 | 0.00161 |
| hsa_circ_0011692 | 1.536 | 13.105 | 4.287 | 0.000164 | 0.00161 |
| hsa_circ_0000204 | 1.494 | 10.867 | 4.282 | 0.000166 | 0.001614 |
| hsa_circ_0002003 | 3.230 | 10.509 | 4.274 | 0.00017 | 0.001626 |
| hsa_circ_0000560 | 1.264 | 7.777 | 4.273 | 0.000171 | 0.001626 |
| hsa_circ_0002754 | 1.613 | 10.909 | 4.263 | 0.000176 | 0.001651 |
| hsa_circ_0070933 | 1.201 | 7.533 | 4.247 | 0.000184 | 0.001708 |
| hsa_circ_0014130 | 1.975 | 7.765 | 4.245 | 0.000185 | 0.00171 |
| hsa_circ_0009131 | 2.118 | 11.181 | 4.244 | 0.000185 | 0.00171 |
| hsa_circ_0057552 | 2.305 | 8.323 | 4.238 | 0.000188 | 0.001722 |
| hsa_circ_0006109 | 1.128 | 9.963 | 4.225 | 0.000196 | 0.001756 |
| hsa_circ_0087493 | 1.299 | 8.598 | 4.214 | 0.000202 | 0.001794 |
| hsa_circ_0001360 | 1.566 | 12.124 | 4.198 | 0.000211 | 0.001853 |
| hsa_circ_0072012 | 1.548 | 8.781 | 4.191 | 0.000215 | 0.001879 |
| hsa_circ_0003892 | 1.338 | 13.490 | 4.178 | 0.000223 | 0.001922 |
| hsa_circ_0028135 | 1.096 | 9.014 | 4.171 | 0.000227 | 0.001937 |
| hsa_circ_0012283 | 1.225 | 7.624 | 4.169 | 0.000228 | 0.001939 |
| hsa_circ_0020749 | 1.278 | 7.335 | 4.164 | 0.000232 | 0.00196 |
| hsa_circ_0029636 | 1.803 | 12.017 | 4.154 | 0.000239 | 0.001987 |
| hsa_circ_0001056 | 3.195 | 10.403 | 4.153 | 0.000239 | 0.001987 |
| hsa_circ_0070348 | 1.144 | 7.691 | 4.145 | 0.000244 | 0.002017 |
| hsa_circ_0060194 | 1.301 | 7.753 | 4.145 | 0.000245 | 0.002017 |
| hsa_circ_0007345 | 1.328 | 10.834 | 4.132 | 0.000253 | 0.002073 |
| hsa_circ_0008773 | 1.436 | 8.933 | 4.127 | 0.000257 | 0.002085 |
| hsa_circ_0055377 | 1.197 | 7.688 | 4.118 | 0.000263 | 0.002128 |
| hsa_circ_0082628 | 1.128 | 14.330 | 4.108 | 0.000271 | 0.002181 |
| hsa_circ_0022587 | 1.484 | 8.979 | 4.102 | 0.000275 | 0.002199 |
| hsa_circ_0054656 | 1.440 | 8.020 | 4.093 | 0.000283 | 0.002229 |
| hsa_circ_0006276 | 1.040 | 11.212 | 4.090 | 0.000285 | 0.002229 |
| hsa_circ_0003695 | 1.366 | 9.651 | 4.084 | 0.00029 | 0.002243 |
| hsa_circ_0008236 | 2.338 | 8.926 | 4.078 | 0.000294 | 0.002262 |
| hsa_circ_0008284 | 1.476 | 8.262 | 4.053 | 0.000316 | 0.002389 |
| hsa_circ_0031431 | 2.968 | 9.571 | 4.033 | 0.000334 | 0.002492 |
| hsa_circ_0007372 | 1.435 | 10.288 | 4.029 | 0.000338 | 0.002507 |
| hsa_circ_0074854 | 3.142 | 11.623 | 4.013 | 0.000354 | 0.002581 |
| hsa_circ_0072255 | 1.281 | 7.245 | 4.005 | 0.000361 | 0.002603 |
| hsa_circ_0068075 | 1.336 | 10.957 | 4.000 | 0.000367 | 0.002627 |
| hsa_circ_0073930 | 1.245 | 9.065 | 3.980 | 0.000387 | 0.002756 |
| hsa_circ_0052523 | 1.258 | 7.237 | 3.964 | 0.000405 | 0.00286 |
| hsa_circ_0068850 | 1.331 | 8.518 | 3.959 | 0.000411 | 0.002889 |
| hsa_circ_0002039 | 1.279 | 8.238 | 3.943 | 0.000429 | 0.00297 |
| hsa_circ_0072430 | 1.259 | 8.775 | 3.943 | 0.000429 | 0.00297 |
| hsa_circ_0063534 | 1.218 | 9.176 | 3.924 | 0.000452 | 0.003071 |
| hsa_circ_0001700 | 1.258 | 7.612 | 3.922 | 0.000455 | 0.003072 |
| hsa_circ_0034786 | 1.455 | 11.019 | 3.916 | 0.000463 | 0.003114 |
| hsa_circ_0034682 | 1.181 | 8.497 | 3.914 | 0.000464 | 0.003114 |
| hsa_circ_0059802 | 1.873 | 7.533 | 3.905 | 0.000477 | 0.003182 |
| hsa_circ_0038821 | 1.347 | 7.731 | 3.899 | 0.000484 | 0.003218 |
| hsa_circ_0007481 | 1.120 | 7.913 | 3.881 | 0.00051 | 0.003346 |
| hsa_circ_0055201 | 1.082 | 8.651 | 3.866 | 0.000531 | 0.003417 |
| hsa_circ_0065964 | 1.557 | 7.756 | 3.866 | 0.000531 | 0.003417 |
| hsa_circ_0076412 | 1.589 | 8.291 | 3.860 | 0.000539 | 0.003439 |
| hsa_circ_0026652 | 1.901 | 11.035 | 3.853 | 0.00055 | 0.003471 |
| hsa_circ_0005256 | 2.578 | 9.924 | 3.851 | 0.000553 | 0.003477 |
| hsa_circ_0006186 | 2.328 | 9.201 | 3.839 | 0.000571 | 0.003563 |
| hsa_circ_0075736 | 2.736 | 9.384 | 3.838 | 0.000573 | 0.003563 |
| hsa_circ_0005405 | 1.017 | 8.223 | 3.791 | 0.000652 | 0.003895 |
| hsa_circ_0012967 | 1.187 | 7.524 | 3.782 | 0.000669 | 0.003939 |
| hsa_circ_0008263 | 2.117 | 8.880 | 3.778 | 0.000676 | 0.003956 |
| hsa_circ_0008223 | 2.346 | 10.688 | 3.773 | 0.000686 | 0.004006 |
| hsa_circ_0044158 | 2.616 | 10.067 | 3.758 | 0.000715 | 0.00415 |
| hsa_circ_0004066 | 2.824 | 9.811 | 3.744 | 0.000743 | 0.004249 |
| hsa_circ_0006461 | 1.542 | 8.933 | 3.744 | 0.000743 | 0.004249 |
| hsa_circ_0041506 | 1.062 | 8.013 | 3.740 | 0.000749 | 0.004274 |
| hsa_circ_0000721 | 2.534 | 9.568 | 3.725 | 0.000781 | 0.004434 |
| hsa_circ_0064324 | 2.071 | 12.687 | 3.721 | 0.000789 | 0.004464 |
| hsa_circ_0070421 | 1.291 | 7.830 | 3.716 | 0.000801 | 0.004489 |
| hsa_circ_0053907 | 1.173 | 9.099 | 3.709 | 0.000816 | 0.004528 |
| hsa_circ_0060262 | 2.262 | 10.157 | 3.704 | 0.000828 | 0.004572 |
| hsa_circ_0041050 | 1.176 | 7.644 | 3.682 | 0.000879 | 0.004824 |
| hsa_circ_0049783 | 2.330 | 12.276 | 3.677 | 0.00089 | 0.00486 |
| hsa_circ_0049785 | 1.880 | 10.465 | 3.675 | 0.000895 | 0.004875 |
| hsa_circ_0035292 | 1.507 | 8.437 | 3.668 | 0.000912 | 0.004937 |
| hsa_circ_0004519 | 2.597 | 10.912 | 3.664 | 0.000922 | 0.004969 |
| hsa_circ_0007476 | 1.028 | 8.295 | 3.661 | 0.000929 | 0.004979 |
| hsa_circ_0004877 | 1.773 | 10.314 | 3.661 | 0.000929 | 0.004979 |
| hsa_circ_0080425 | 1.247 | 14.424 | 3.654 | 0.000949 | 0.005069 |
| hsa_circ_0082711 | 1.046 | 8.071 | 3.641 | 0.000982 | 0.005221 |
| hsa_circ_0003026 | 1.044 | 8.140 | 3.628 | 0.001016 | 0.005372 |
| hsa_circ_0077527 | 2.829 | 10.956 | 3.627 | 0.00102 | 0.005378 |
| hsa_circ_0081342 | 1.769 | 9.473 | 3.624 | 0.001027 | 0.005402 |
| hsa_circ_0006211 | 1.377 | 14.035 | 3.624 | 0.00103 | 0.005402 |
| hsa_circ_0040148 | 1.311 | 10.046 | 3.615 | 0.001054 | 0.005516 |
| hsa_circ_0000257 | 1.823 | 12.096 | 3.604 | 0.001084 | 0.005645 |
| hsa_circ_0065173 | 1.535 | 8.947 | 3.595 | 0.001113 | 0.005765 |
| hsa_circ_0007676 | 1.433 | 7.899 | 3.586 | 0.001139 | 0.005871 |
| hsa_circ_0052131 | 2.521 | 9.632 | 3.577 | 0.001166 | 0.00595 |
| hsa_circ_0004720 | 1.923 | 10.205 | 3.544 | 0.001277 | 0.00637 |
| hsa_circ_0003141 | 3.648 | 12.354 | 3.524 | 0.001347 | 0.00665 |
| hsa_circ_0000936 | 2.051 | 8.942 | 3.523 | 0.00135 | 0.006652 |
| hsa_circ_0005327 | 1.610 | 8.444 | 3.520 | 0.00136 | 0.006668 |
| hsa_circ_0023404 | 1.302 | 11.817 | 3.510 | 0.001397 | 0.006818 |
| hsa_circ_0082139 | 1.273 | 8.412 | 3.485 | 0.001493 | 0.00718 |
| hsa_circ_0028944 | 1.180 | 8.295 | 3.454 | 0.001623 | 0.007649 |
| hsa_circ_0006913 | 2.155 | 9.070 | 3.444 | 0.001668 | 0.007731 |
| hsa_circ_0007772 | 1.156 | 9.143 | 3.440 | 0.001685 | 0.007753 |
| hsa_circ_0000238 | 1.064 | 10.803 | 3.438 | 0.001693 | 0.007753 |
| hsa_circ_0038872 | 1.103 | 8.067 | 3.429 | 0.001737 | 0.007866 |
| hsa_circ_0004315 | 1.631 | 10.816 | 3.416 | 0.001797 | 0.008062 |
| hsa_circ_0005567 | 1.560 | 8.658 | 3.415 | 0.0018 | 0.008062 |
| hsa_circ_0001658 | 3.307 | 11.692 | 3.414 | 0.001804 | 0.008062 |
| hsa_circ_0009792 | 1.698 | 8.609 | 3.414 | 0.001805 | 0.008062 |
| hsa_circ_0050486 | 1.442 | 14.319 | 3.409 | 0.001832 | 0.008133 |
| hsa_circ_0039557 | 1.213 | 12.460 | 3.405 | 0.001847 | 0.008184 |
| hsa_circ_0005699 | 2.365 | 13.026 | 3.400 | 0.001877 | 0.008259 |
| hsa_circ_0069104 | 1.367 | 12.183 | 3.371 | 0.002023 | 0.00875 |
| hsa_circ_0006629 | 1.065 | 9.554 | 3.365 | 0.002054 | 0.008847 |
| hsa_circ_0027774 | 1.418 | 10.625 | 3.356 | 0.002108 | 0.00906 |
| hsa_circ_0001730 | 1.214 | 8.250 | 3.354 | 0.002117 | 0.00908 |
| hsa_circ_0016863 | 1.461 | 9.065 | 3.341 | 0.002193 | 0.009363 |
| hsa_circ_0020934 | 2.190 | 12.849 | 3.324 | 0.002291 | 0.009658 |
| hsa_circ_0000993 | 2.539 | 9.316 | 3.309 | 0.002382 | 0.00992 |
| hsa_circ_0003763 | 1.585 | 11.028 | 3.307 | 0.002395 | 0.009956 |
| hsa_circ_0003215 | 1.113 | 11.963 | 3.296 | 0.002466 | 0.010188 |
| hsa_circ_0006827 | 2.279 | 12.383 | 3.274 | 0.002613 | 0.010642 |
| hsa_circ_0059859 | 1.123 | 10.372 | 3.269 | 0.002647 | 0.01076 |
| hsa_circ_0008911 | 1.002 | 8.013 | 3.267 | 0.002661 | 0.010794 |
| hsa_circ_0029605 | 1.464 | 8.096 | 3.263 | 0.002692 | 0.010875 |
| hsa_circ_0083756 | 1.558 | 10.751 | 3.254 | 0.002752 | 0.011053 |
| hsa_circ_0066380 | 2.096 | 11.106 | 3.249 | 0.002792 | 0.011169 |
| hsa_circ_0050867 | 3.014 | 12.280 | 3.245 | 0.00282 | 0.011235 |
| hsa_circ_0007669 | 1.145 | 7.806 | 3.240 | 0.002855 | 0.011354 |
| hsa_circ_0007895 | 2.476 | 10.016 | 3.233 | 0.002906 | 0.011466 |
| hsa_circ_0007991 | 1.577 | 11.931 | 3.228 | 0.002947 | 0.011582 |
| hsa_circ_0062261 | 1.013 | 9.352 | 3.212 | 0.003076 | 0.011995 |
| hsa_circ_0003505 | 1.451 | 11.634 | 3.209 | 0.003094 | 0.012044 |
| hsa_circ_0007024 | 1.825 | 13.855 | 3.206 | 0.003123 | 0.012086 |
| hsa_circ_0012265 | 1.493 | 10.614 | 3.200 | 0.003171 | 0.012177 |
| hsa_circ_0013048 | 1.068 | 11.908 | 3.199 | 0.003177 | 0.012177 |
| hsa_circ_0007378 | 2.211 | 12.213 | 3.199 | 0.003177 | 0.012177 |
| hsa_circ_0031250 | 2.759 | 9.851 | 3.193 | 0.003229 | 0.012306 |
| hsa_circ_0067494 | 1.451 | 8.184 | 3.189 | 0.003264 | 0.012392 |
| hsa_circ_0041019 | 1.596 | 7.818 | 3.186 | 0.003283 | 0.012422 |
| hsa_circ_0082582 | 1.140 | 13.709 | 3.186 | 0.003287 | 0.012422 |
| hsa_circ_0004380 | 1.119 | 7.689 | 3.186 | 0.00329 | 0.012422 |
| hsa_circ_0001721 | 1.930 | 9.411 | 3.180 | 0.003342 | 0.012596 |
| hsa_circ_0005043 | 1.110 | 8.078 | 3.177 | 0.003367 | 0.012642 |
| hsa_circ_0006608 | 2.276 | 12.496 | 3.175 | 0.00338 | 0.012642 |
| hsa_circ_0004200 | 1.340 | 10.871 | 3.167 | 0.003455 | 0.012875 |
| hsa_circ_0018814 | 1.203 | 8.466 | 3.162 | 0.003497 | 0.012939 |
| hsa_circ_0002976 | 1.216 | 8.307 | 3.150 | 0.003608 | 0.013229 |
| hsa_circ_0000520 | 2.176 | 11.163 | 3.148 | 0.003624 | 0.013261 |
| hsa_circ_0086686 | 1.913 | 10.716 | 3.141 | 0.003697 | 0.01343 |
| hsa_circ_0015379 | 1.092 | 9.316 | 3.112 | 0.003979 | 0.014253 |
| hsa_circ_0007158 | 1.771 | 12.390 | 3.071 | 0.004421 | 0.01553 |
| hsa_circ_0008539 | 1.085 | 8.929 | 3.064 | 0.0045 | 0.015713 |
| hsa_circ_0040507 | 1.176 | 11.038 | 3.064 | 0.004504 | 0.015713 |
| hsa_circ_0064557 | 2.852 | 11.679 | 3.058 | 0.004568 | 0.015881 |
| hsa_circ_0053317 | 1.199 | 7.775 | 3.049 | 0.004677 | 0.016149 |
| hsa_circ_0020097 | 1.409 | 8.043 | 3.047 | 0.004699 | 0.016198 |
| hsa_circ_0004780 | 1.048 | 8.388 | 3.004 | 0.00524 | 0.017524 |
| hsa_circ_0071681 | 1.132 | 12.065 | 3.003 | 0.005253 | 0.01753 |
| hsa_circ_0004757 | 2.048 | 13.602 | 3.001 | 0.005276 | 0.01753 |
| hsa_circ_0049998 | 1.151 | 9.037 | 3.000 | 0.005289 | 0.017542 |
| hsa_circ_0008121 | 1.419 | 10.643 | 2.994 | 0.005373 | 0.017764 |
| hsa_circ_0006837 | 1.201 | 9.248 | 2.990 | 0.005436 | 0.017902 |
| hsa_circ_0009053 | 1.854 | 10.251 | 2.989 | 0.005441 | 0.017902 |
| hsa_circ_0067934 | 2.027 | 12.521 | 2.977 | 0.005612 | 0.018345 |
| hsa_circ_0007883 | 2.018 | 8.951 | 2.977 | 0.005614 | 0.018345 |
| hsa_circ_0054033 | 1.097 | 10.520 | 2.976 | 0.005622 | 0.018345 |
| hsa_circ_0070396 | 1.039 | 8.030 | 2.969 | 0.005722 | 0.018614 |
| hsa_circ_0003342 | 1.149 | 9.062 | 2.967 | 0.005758 | 0.0187 |
| hsa_circ_0015936 | 1.077 | 7.830 | 2.937 | 0.006208 | 0.019906 |
| hsa_circ_0008001 | 1.886 | 9.154 | 2.934 | 0.006255 | 0.020024 |
| hsa_circ_0011636 | 1.032 | 9.075 | 2.922 | 0.006441 | 0.02048 |
| hsa_circ_0000871 | 1.013 | 12.032 | 2.870 | 0.00734 | 0.022637 |
| hsa_circ_0005038 | 1.300 | 9.890 | 2.859 | 0.007548 | 0.023101 |
| hsa_circ_0040039 | 1.673 | 11.844 | 2.828 | 0.008137 | 0.02457 |
| hsa_circ_0082142 | 1.386 | 8.011 | 2.819 | 0.008326 | 0.024985 |
| hsa_circ_0044226 | 1.038 | 7.503 | 2.817 | 0.008361 | 0.024985 |
| hsa_circ_0046419 | 1.908 | 9.464 | 2.805 | 0.008619 | 0.025566 |
| hsa_circ_0002733 | 1.230 | 10.452 | 2.800 | 0.008728 | 0.025664 |
| hsa_circ_0002657 | 1.295 | 9.020 | 2.784 | 0.009071 | 0.026478 |
| hsa_circ_0057214 | 1.359 | 8.352 | 2.773 | 0.009322 | 0.027124 |
| hsa_circ_0024143 | 1.242 | 10.190 | 2.748 | 0.009911 | 0.028278 |
| hsa_circ_0068610 | 1.911 | 9.974 | 2.737 | 0.010182 | 0.029009 |
| hsa_circ_0006278 | 1.042 | 8.952 | 2.723 | 0.010546 | 0.029754 |
| hsa_circ_0003645 | 1.351 | 14.071 | 2.714 | 0.010768 | 0.030251 |
| hsa_circ_0001707 | 1.422 | 10.176 | 2.694 | 0.011292 | 0.031332 |
| hsa_circ_0075410 | 1.469 | 9.394 | 2.685 | 0.011561 | 0.03199 |
| hsa_circ_0078486 | 1.242 | 8.593 | 2.684 | 0.011584 | 0.032011 |
| hsa_circ_0005927 | 2.861 | 11.357 | 2.661 | 0.012229 | 0.033414 |
| hsa_circ_0018659 | 1.328 | 8.438 | 2.625 | 0.013329 | 0.035756 |
| hsa_circ_0079375 | 1.337 | 10.877 | 2.625 | 0.013354 | 0.035775 |
| hsa_circ_0000517 | 1.391 | 9.169 | 2.621 | 0.013486 | 0.03594 |
| hsa_circ_0000894 | 1.042 | 9.031 | 2.607 | 0.013938 | 0.036826 |
| hsa_circ_0050590 | 1.054 | 7.827 | 2.606 | 0.013971 | 0.036826 |
| hsa_circ_0059702 | 1.565 | 10.962 | 2.604 | 0.014029 | 0.036851 |
| hsa_circ_0009172 | 1.369 | 12.877 | 2.600 | 0.014174 | 0.037087 |
| hsa_circ_0002224 | 1.095 | 11.181 | 2.589 | 0.014526 | 0.037764 |
| hsa_circ_0027355 | 1.065 | 9.094 | 2.568 | 0.01529 | 0.039001 |
| hsa_circ_0075629 | 1.005 | 8.267 | 2.537 | 0.01645 | 0.041726 |
| hsa_circ_0017956 | 1.085 | 8.086 | 2.503 | 0.017792 | 0.044065 |
| hsa_circ_0009361 | 1.365 | 13.360 | 2.498 | 0.018013 | 0.044508 |
| hsa_circ_0046435 | 1.370 | 10.959 | 2.473 | 0.019091 | 0.04663 |
| hsa_circ_0008514 | 1.004 | 7.989 | 2.445 | 0.020388 | 0.048853 |
| hsa_circ_0064644 | 1.287 | 8.280 | 2.439 | 0.020651 | 0.049308 |
