# Supplementary material for: CircMTO1 suppresses hepatocellular carcinoma progression via the miR-541-5p/ZIC1 axis by regulating Wnt/β-catenin signaling pathway and epithelial-to-mesenchymal transition
Source: Cell Death Dis. 2021 Dec 20;13(1):12. doi: 10.1038/s41419-021-04464-3 (PMC8688446; doi:10.1038/s41419-021-04464-3)
Supplement: Supplementary file 3 — Table S2 [file 41419_2021_4464_MOESM3_ESM.docx]

Table S2 The miRNAs may bind to circMTO1 predicted by ENCORI/starbase(http://starbase.sysu.edu.cn/), CSCD (http://gb.whu.edu.cn/CSCD/), circbank and miRanda databases.

| ENCORI/starbase | CSCD | circbank | miRanda |
| --- | --- | --- | --- |
| miRNAs | | | |
| miR-489-3p | miR-1252-5p | miR-204-5p | miR-15a-3p |
| miR-541-5p | miR-1256 | miR-211-5p | miR-29b-1-5p |
| miR-204-5p | miR-1273g-3p | miR-6768-5p | miR-199a-5p |
| miR-211-5p | miR-181a-2-3p | miR-1207-3p | miR-181a-2-3p |
| miR-3200-5p | miR-1825 | miR-152-5p | miR-199b-5p |
| miR-760 | miR-199-5p | miR-1972 | miR-204-5p |
| miR-199a-5p | miR-204-5p | miR-199a-5p | miR-211-5p |
| miR-199b-5p | miR-211-5p | miR-199b-5p | miR-218-2-3p |
| miR-218-5p | miR-2467-5p | miR-29b-1-5p | miR-223-5p |
| miR-448 | miR-3064-3p | miR-3155a | miR-124-5p |
| miR-199a-3p | miR-3124-3p | miR-3155b | miR-152-5p |
| miR-199b-3p | miR-3159 | miR-3159 | miR-9-5p |
| miR-3129-5p | miR-3202 | miR-3686 | miR-138-1-3p |
| miR-515-5p | miR-345-5p | miR-4476 | miR-148b-5p |
| miR-519e-5p | miR-3660 | miR-4494 | miR-484 |
| miR-1287-5p | miR-4526 | miR-4533 | miR-489-3p |
| miR-370-3p | miR-3913-3p | miR-4666b | miR-501-3p |
| miR-6893-3p | miR-425-5p | miR-541-5p | miR-502-3p |
| miR-337-3p | miR-4256 | miR-544a | miR-513a-3p |
| miR-182-5p | miR-4286 | miR-5683 | miR-509-3p |
| miR-200c-3p | miR-4301 | miR-6507-5p | miR-544a |
| miR-429 | miR-4302 | miR-6730-3p | miR-576-3p |
| miR-200b-3p | miR-4304 | miR-6736-3p | miR-602 |
| miR-4782-3p | miR-4433b-5p | miR-6876-5p | miR-617 |
| miR-6766-3p | miR-4446-5p | miR-7161-3p | miR-642a-5p |
| miR-219a-5p | miR-4462 | miR-760 | miR-659-3p |
| miR-320a | miR-4476 | miR-888-3p | miR-660-3p |
| miR-320b | miR-6876-5p |  | miR-454-5p |
| miR-9-3p | miR-4533 |  | miR-874-5p |
| miR-320c | miR-4666-5p |  | miR-888-3p |
| miR-4429 | miR-4715-3p |  | miR-541-5p |
| miR-320d | miR-4750-3p |  | miR-876-5p |
| miR-802 | miR-513-3p |  | miR-760 |
| miR-4465 | miR-541-5p |  | miR-1207-3p |
| miR-26a-5p | miR-548-3p |  | miR-513c-3p |
| miR-26b-5p | miR-1323 |  | miR-1972 |
| miR-1297 | miR-548 |  | miR-2682-5p |
| miR-296-3p | miR-4445-3p |  | miR-548 |
| miR-216a-5p | miR-602 |  | miR-3155a |
| miR-376a-3p | miR-6504-3p |  | miR-3156-5p |
| miR-376b-3p | miR-651-3p |  | miR-3159 |
| miR-4739 | miR-652-5p |  | miR-3200-5p |
| miR-4756-5p | miR-6719-3p |  | miR-4302 |
| miR-1321 | miR-6730-3p |  | miR-4303 |
| miR-379-3p | miR-6733-3p |  | miR-4286 |
| miR-411-3p | miR-6793-3p |  | miR-3615 |
| miR-202-5p | miR-6832-3p |  | miR-3653-5p |
| miR-1294 | miR-6834-3p |  | miR-3657 |
| miR-584-5p | miR-6846-3p |  | miR-3686 |
| miR-30c-5p | miR-6859-3p |  | miR-3910 |
| miR-30b-5p | miR-7977 |  | miR-3916 |
| miR-30a-5p | miR-888-3p |  | miR-3927-5p |
| miR-30d-5p |  |  | miR-3934-5p |
| miR-30e-5p |  |  | miR-3938 |
| miR-191-5p |  |  | miR-642b-5p |
| miR-3127-5p |  |  | miR-4462 |
| miR-374a-3p |  |  | miR-4463 |
| miR-330-5p |  |  | miR-4469 |
| miR-326 |  |  | miR-4476 |
| miR-499b-5p |  |  | miR-3155 |
| miR-212-5p |  |  | miR-4494 |
| miR-670-5p |  |  | miR-4519 |
| miR-642b-3p |  |  | miR-4520-5p |
| miR-642a-3p |  |  | miR-4529-5p |
|  |  |  | miR-4533 |
|  |  |  | miR-4646-3p |
|  |  |  | miR-4652-3p |
|  |  |  | miR-4667-3p |
|  |  |  | miR-4672 |
|  |  |  | miR-4677-3p |
|  |  |  | miR-4682 |
|  |  |  | miR-4704-5p |
|  |  |  | miR-4715-3p |
|  |  |  | miR-4737 |
|  |  |  | miR-4778-5p |
|  |  |  | miR-5004-5p |
|  |  |  | miR-5006-3p |
|  |  |  | miR-5100 |
|  |  |  | miR-5587-5p |
|  |  |  | miR-5683 |
|  |  |  | miR-4666b |
|  |  |  | miR-6131 |
|  |  |  | miR-6507-5p |
|  |  |  | miR-6514-5p |
|  |  |  | miR-6730-3p |
|  |  |  | miR-6734-3p |
|  |  |  | miR-6736-3p |
|  |  |  | miR-6764-3p |
|  |  |  | miR-6768-5p |
|  |  |  | miR-6792-5p |
|  |  |  | miR-6824-3p |
|  |  |  | miR-6825-3p |
|  |  |  | miR-6830-5p |
|  |  |  | miR-6834-3p |
|  |  |  | miR-6848-3p |
|  |  |  | miR-6876-5p |
|  |  |  | miR-7106-3p |
|  |  |  | miR-7152-3p |
|  |  |  | miR-7161-3p |
