# Supplementary material for: CircMTO1 suppresses hepatocellular carcinoma progression via the miR-541-5p/ZIC1 axis by regulating Wnt/β-catenin signaling pathway and epithelial-to-mesenchymal transition
Source: Cell Death Dis. 2021 Dec 20;13(1):12. doi: 10.1038/s41419-021-04464-3 (PMC8688446; doi:10.1038/s41419-021-04464-3)
Supplement: Supplementary file 4 — Table S3 [file 41419_2021_4464_MOESM4_ESM.docx]

Table S3 The possible miR-541-5p target genes predicted by miRDB, TargetScan and ENCORI/starbase databases.

| miRDB | TargetScan | ENCORI |
| --- | --- | --- |
| target genes | | |
| ULK2 | ZNF208 | DDX3Y |
| ANKRD46 | ZNF676 | DFFA |
| ASTE1 | RBBP4 | RCC2 |
| NAP1L1 | C12orf5 | IFFO2 |
| WNK3 | ZNF256 | PPT1 |
| MAPK1 | EBLN1 | ATPAF1 |
| FBXO33 | PAGE2B | NDC1 |
| CLEC6A | PAGE2 | MYSM1 |
| CEP112 | GNRH1 | SLC35D1 |
| PICALM | ASTE1 | SERBP1 |
| ZNF781 | CST11 | ANKRD13C |
| TEP1 | C12orf75 | DNTTIP2 |
| TMPRSS11A | C8orf34 | SLC16A4 |
| MAGEB2 | ZIK1 | RSBN1 |
| CHD6 | CLC | PTPN22 |
| GFPT1 | CEP112 | NRAS |
| CCDC82 | ARMS2 | IGSF3 |
| VPS13C | ZCRB1 | WARS2 |
| PTGDR | TIPIN | OTUD7B |
| EIF5A2 | IFT52 | ENSA |
| TRHDE | ZNF781 | GATAD2B |
| GLTP | KRTAP9-8 | ADAR |
| NCAN | APIP | ASH1L |
| DERL1 | KRTAP9-2 | KHDC4 |
| CTDSPL2 | PANK2 | MEX3A |
| TIGAR | NRM | HDGF |
| PRRC2C | DENND1B | PIGM |
| FRMD3 | CD1E | B4GALT3 |
| KPNA1 | TINF2 | CREG1 |
| MAP4K5 | PPHLN1 | RC3H1 |
| CDK6 | MT2A | EDEM3 |
| GATAD2A | GLTP | DENND1B |
| SGTB | MAGEB2 | ZNF281 |
| NUDT19 | SMIM10 | KDM5B |
| MARF1 | GABARAPL2 | KLHL12 |
| JAZF1 | RMI1 | ELK4 |
| KIF3A | SYAP1 | NUCKS1 |
| COPS2 | RNPEP | YOD1 |
| TMEM161B | CDC42EP4 | RAB3GAP2 |
| SAXO2 | GFPT1 | DUSP10 |
| LUM | PICALM | LBR |
| LRRFIP1 | TMED7 | CDC42BPA |
| PIGM | SERF1B | CCSAP |
| RHNO1 | SERF1A | EXOC8 |
| MIPOL1 | PTGR2 | ARID4B |
| C18orf32 | CLEC6A | CEP170 |
| VPS13A | AL590452.1 | AKT3 |
| CNTN3 | LSMEM2 | MFN2 |
| TM7SF3 | ATXN7L3B | PRDM2 |
| RPL17-C18orf32 | SPZ1 | DDI2 |
| DHX40 | ARGLU1 | ZBTB40 |
| SOCS4 | FAM78B | PITHD1 |
| CLVS2 | LUM | CLIC4 |
| SLC7A6 | C11orf82 | FAM76A |
| GSPT1 | 44257 | ATP5IF1 |
| NR3C2 | SH3YL1 | RCC1 |
| GRM5 | RBM17 | KPNA6 |
| MED14 | HIST1H4I | RBBP4 |
| RAPH1 | ZMYND19 | AGO1 |
| FRS2 | LRRFIP1 | ZMPSTE24 |
| SPRED1 | LRRC7 | ZFP69B |
| ARFIP1 | NRG4 | SRSF11 |
| MED13 | P2RX3 | VCAM1 |
| CCDC141 | ST8SIA4 | PRPF38B |
| PRKG2 | BOLL | TMEM167B |
| UBE2W | ZNF728 | AHCYL1 |
| PAQR3 | CDK8 | CTTNBP2NL |
| SCG3 | TNFRSF8 | DCLRE1B |
| ABCB7 | CPA3 | HIPK1 |
| TINF2 | PDCD5 | MAN1A2 |
| PLXDC2 | RGS21 | NOTCH2NL |
| LRRC7 | HMMR | PDZK1 |
| RAD51B | COPS2 | BCL9 |
| MEX3A | METTL15 | CIART |
| TMEM248 | GMFB | RPRD2 |
| CHD8 | LCLAT1 | MLLT11 |
| ZNF708 | IL22RA2 | ZNF687 |
| LSMEM2 | ANGPTL3 | SNX27 |
| TENM3 | GYPE | HCN3 |
| RPS6KA3 | TM6SF1 | UHMK1 |
| GABRG2 | AAGAB | PBX1 |
| APIP | TMEM161B | POU2F1 |
| C8orf34 | EIF5A2 | C1orf112 |
| SPON1 | RP11-861L17.3 | PRRC2C |
| SLC46A3 | DDX4 | PRDX6 |
| LPP | ESPL1 | RABGAP1L |
| ZNF326 | FBXO33 | SOAT1 |
| FGF14 | KCTD12 | XPR1 |
| DHX33 | PRPF38B | NEK7 |
| NRM | GRPEL2 | NAV1 |
| CDH8 | ZNF345 | RNPEP |
| DGKI | BLOC1S6 | PPP1R12B |
| ZNF586 | CD180 | MDM4 |
| GLIPR1 | AL627171.2 | PROX1 |
| RBM17 | PCDHB6 | DISP1 |
| GNPNAT1 | AL360004.1 | FBXO28 |
| CLC | ZNF189 | LARP4B |
| GABARAPL2 | SPRED1 | PIP4K2A |
| CAMSAP2 | UEVLD | EPC1 |
| CPA3 | PANK3 | HNRNPF |
| IFT52 | ZNF586 | RTKN2 |
| RNF144A | SLMO2 | RUFY2 |
| VEZF1 | PRSS12 | DNA2 |
| PRELID3B | PAQR3 | TCTN3 |
| SMNDC1 | SAMD14 | CCDC186 |
| FBXO25 | FGF16 | IKZF5 |
| PCDHGB7 | RSBN1L | EEF1AKMT2 |
| PM20D2 | DERL2 | ADAM12 |
| ING3 | DCAF13 | MKI67 |
| C12orf75 | RBM43 | EBF3 |
| TMED7 | CCDC47 | RBM17 |
| CST11 | ENY2 | STAM |
| RUFY2 | CTD-2616J11.4 | KIAA1217 |
| ADAMTSL3 | CCNH | ZEB1 |
| EFNB2 | OR2M3 | CSGALNACT2 |
| NUDT12 | ZNF708 | H2AFY2 |
| USP38 | CASP1 | ANAPC16 |
| SLC16A4 | HINFP | VCL |
| MYF5 | GPX8 | KAT6B |
| PKD1L1 | IVL | GHITM |
| ZFYVE16 | C7orf76 | PTEN |
| GRID2 | SYNGR4 | CCNJ |
| SLFN13 | GALNTL6 | PGAM1 |
| THAP10 | RAB9B | ZDHHC16 |
| ENOSF1 | GSTM2 | SLF2 |
| ZNF189 | SEC63 | BTRC |
| ZNF426 | MDM4 | WBP1L |
| C10orf25 | TMX4 | SHOC2 |
| HIP1 | MTRNR2L11 | VTI1A |
| DERL2 | PCTP | HTRA1 |
| BACE1 | LDB3 | ZNF195 |
| SDC2 | ACER3 | CAVIN3 |
| FBXW2 | RNASE8 | SBF2 |
| CDC42BPA | ATPIF1 | RNF141 |
| NDST2 | DAB2 | UEVLD |
| CATSPERE | CLVS2 | CD59 |
| TMOD2 | FABP1 | AL049629.2 |
| CSTB | LINS | FBXO3 |
| VCAN | NMU | SLC1A2 |
| ZNF773 | KLHL12 | CKAP5 |
| USP9X | SNX13 | CELF1 |
| F5 | ST6GAL2 | CHKA |
| ATG4C | PCDHB11 | KMT5B |
| ZNF333 | RP1-241P17.4 | SHANK2 |
| KCTD12 | CREG1 | CLNS1A |
| CAPRIN2 | RP11-542P2.1 | RSF1 |
| TOX | SPAM1 | NARS2 |
| SCAMP1 | GABRG2 | CREBZF |
| TNFRSF8 | UPP2 | PICALM |
| TTC21B | DHX33 | EXPH5 |
| ANGPTL3 | SLC46A3 | ALG9 |
| PITPNB | EIF1AY | TRIM22 |
| MYO5A | GLB1L3 | ARNTL |
| SLC6A1 | NRG3 | FAR1 |
| FNIP1 | KIAA1191 | SPON1 |
| ZSCAN25 | PPTC7 | HTATIP2 |
| ANAPC16 | TEX36 | METTL15 |
| ERC1 | UGT2B10 | QSER1 |
| ARGLU1 | PCMTD2 | PTPRJ |
| REV1 | PCDHB3 | CTNND1 |
| NCKAP5 | ZNF528 | ZFP91 |
| IL33 | PI15 | RBM4 |
| LPCAT2 | PF4 | CCND1 |
| MACC1 | INHBA | EMSY |
| GLI3 | DHX40 | ACER3 |
| KHDC4 | MAP4K5 | DDIAS |
| CD74 | CCL22 | TMEM135 |
| ESPL1 | CTC-241N9.1 | ZC3H12C |
| ELOVL6 | INHBB | PAFAH1B2 |
| PAX6 | ZNF451 | KMT2A |
| ONECUT2 | ANKRD46 | ARCN1 |
| SEC24A | N4BP2L1 | C12orf4 |
| ENC1 | C18orf32 | MFAP5 |
| ELAVL1 | MBD1 | DUSP16 |
| EPHA3 | SMIM9 | SOX5 |
| NEK7 | FFAR2 | KRAS |
| SAMD14 | RNPC3 | ERGIC2 |
| ELOVL7 | DTX3L | CAPRIN2 |
| NEK10 | TMPRSS11A | DENND5B |
| PPHLN1 | ATP5J2 | CPNE8 |
| NRAS | MYOZ1 | ARF3 |
| SRSF8 | ZNF25 | CBX5 |
| ZNF676 | STOM | GLS2 |
| DCLK1 | CAPRIN2 | PTGES3 |
| PRSS36 | TMPRSS11B | GRIP1 |
| QSER1 | MBD3L1 | CPM |
| AGTR2 | PKD1L1 | ZFC3H1 |
| EPB41L4B | CPOX | NAP1L1 |
| TBL1XR1 | C10orf40 | PAWR |
| MTRF1L | C10orf25 | ATP2B1 |
| CPSF6 | GDI1 | LUM |
| SH3PXD2B | RUFY2 | EEA1 |
| SNX2 | ULK2 | FGD6 |
| AMFR | GRIA4 | PAH |
| PTGR2 | ZNF544 | NT5DC3 |
| MEF2A | VWC2 | WSB2 |
| HES5 | AC090186.1 | CAMKK2 |
| MMRN1 | SUMO2 | ERC1 |
| TMX4 | GNA12 | RHNO1 |
| PNPLA8 | FCGR3B | TIGAR |
| EPC1 | UST | NANOG |
| TMEM255A | CCNG2 | RASSF8 |
| SH3BP2 | FAM154B | FGFR1OP2 |
| BMS1 | SPAG6 | ARNTL2 |
| DCAF5 | PRKCDBP | FGD4 |
| ZKSCAN1 | SMIM6 | PPHLN1 |
| APOLD1 | BTG1 | LARP4 |
| IFIT1 | RBM12B-AS1 | DAZAP2 |
| PCDHB11 | ZG16 | ZNF740 |
| TTC33 | KRT12 | ESPL1 |
| DENND3 | JMJD6 | SP1 |
| PHF6 | CMC2 | RAB5B |
| MED23 | SPOCK1 | SPRYD4 |
| PCDHB6 | CER1 | USP15 |
| SRSF12 | RHNO1 | HMGA2 |
| PPP2R5E | IL33 | DYRK2 |
| FMN1 | C11orf44 | MDM2 |
| CHCHD7 | PHF6 | CPSF6 |
| CASP10 | ZNF195 | FRS2 |
| SETD9 | CBLN2 | THAP2 |
| KIAA1191 | KRT26 | ATXN7L3B |
| GRID1 | UFL1 | MRPL42 |
| ZNF138 | TMEM248 | ACTR6 |
| SYAP1 | PAH | CHPT1 |
| DISC1 | C1orf51 | PARPBP |
| PRSS12 | NRAS | CHST11 |
| C1orf189 | STMN4 | WASHC4 |
| PAH | H2AFY2 | C12orf75 |
| GRSF1 | TIMMDC1 | PTPN11 |
| SERINC5 | TTC33 | XPO4 |
| MDM2 | SLU7 | SLC46A3 |
| NRG3 | KIAA0907 | HMGB1 |
| SMAP1 | CTDSPL2 | KBTBD6 |
| DZIP1 | FAM107B | TSC22D1 |
| FGD4 | PDE11A | INTS6 |
| PDGFD | TBCK | KCTD12 |
| PNKD | FHL2 | DZIP1 |
| IFNG | KLF17 | ZMYM2 |
| CPNE8 | DYNLT3 | PAN3 |
| ZEB1 | GRB2 | MEDAG |
| SLC25A53 | LSM14B | NHLRC3 |
| FNIP2 | AC079210.1 | AKAP11 |
| FAM78B | SGTB | ARL11 |
| GALK2 | ZNF257 | GPR180 |
| EAF1 | CPNE8 | TM9SF2 |
| ZNF618 | SEC14L3 | CHD8 |
| ZNF268 | GRID2 | TINF2 |
| GRPEL2 | SPINK2 | HEATR5A |
| ANKRD13C | FCGR3A | AL139353.1 |
| MUC21 | MYF5 | SPTSSA |
| EYS | PGAM4 | PPP2R3C |
| ZNF728 | CCDC126 | SEC23A |
| FAM122C | USP15 | FBXO33 |
| DENND1B | MTRF1L | MAP4K5 |
| APOPT1 | ZBTB6 | ERO1A |
| PTGIS | GALT | GNPNAT1 |
| PAPPA | ZNF148 | PPP2R5E |
| ATP2B1 | RPL17-C18orf32 | NUMB |
| PUM2 | GNPNAT1 | AREL1 |
| PCMTD2 | ZC4H2 | TMED10 |
| LRRTM2 | AK2 | SPTLC2 |
| KLF17 | MGLL | SEL1L |
| METTL15 | ELOVL6 | BTBD7 |
| SPEF2 | SUMO1 | PAX9 |
| CCPG1 | ZNF157 | SSTR1 |
| WDR72 | GNAI3 | PNN |
| ZCRB1 | LYZL4 | FRMD6 |
| DYNC1I1 | BMP3 | SOCS4 |
| ARL4C | RNF144A | KTN1 |
| HNRNPD | OARD1 | DAAM1 |
| HELZ | XRN2 | HIF1A |
| ST8SIA2 | C12orf76 | CHURC1 |
| SETD6 | KRTAP20-2 | PCNX1 |
| DDX4 | GALNT11 | PSEN1 |
| RBM43 | PLCXD3 | PTGR2 |
| TMEM106B | MFAP5 | BBOF1 |
| TAL1 | C15orf62 | EIF2B2 |
| SYNCRIP | NUDT19 | WDR20 |
| ZBTB6 | ANKRD39 | UBE3A |
| ZMYND19 | METTL10 | GOLGA8A |
| CD1E | CSTB | GOLGA8B |
| ZNF780A | ARG1 | AQR |
| PANK1 | KPNA1 | TP53BP1 |
| CEMIP2 | RNLS | COPS2 |
| NUFIP2 | MICU3 | MYO5A |
| UEVLD | AC012360.2 | FAM214A |
| GNAS | IL17A | WDR72 |
| BACH2 | ENAM | PRTG |
| FAM114A1 | ZNF80 | VPS13C |
| SNAI2 | PPT1 | PARP16 |
| RNPEP | RNF113B | IGDCC4 |
| LPIN2 | EIF2B2 | TIPIN |
| ARMS2 | GTF2E1 | AAGAB |
| RPL15 | TNR | CT62 |
| SLITRK4 | AKAP17A | NPTN |
| CMC2 | BCL2L10 | MAN2C1 |
| PARP15 | RAC1 | C15orf40 |
| PCDHB3 | XG | HDGFL3 |
| LPCAT1 | KCNJ13 | PEX11A |
| PRDM2 | VPS36 | AP3S2 |
| FGFR1OP2 | DSG4 | C15orf38-AP3S2 |
| PLAA | CLIC4 | SPRED1 |
| ASPH | ANKRD34B | MGA |
| KRTAP9-2 | FAXDC2 | CTDSPL2 |
| SLFN11 | CAMK1G | BLOC1S6 |
| RSBN1L | SCGB2A2 | TMOD2 |
| SMIM10 | C14orf142 | RNF111 |
| OTULIN | UBXN2B | MAP2K1 |
| ZNF629 | SRSF11 | FBXO22 |
| ARPIN-AP3S2 | PLVAP | CHRNA5 |
| TMCC3 | MS4A3 | TM6SF1 |
| AP3S2 | GJA3 | ARRDC4 |
| PI15 | PLAT | MEF2A |
| WWC2 | VWC2L | GLYR1 |
| MOB1A | IFNG | PPL |
| BDP1 | FAM172A | CARHSP1 |
| RMND5A | CHD8 | GRIN2A |
| GNRH1 | CD74 | TXNDC11 |
| BICC1 | FRMD3 | GSPT1 |
| SESTD1 | KRTAP10-3 | NTAN1 |
| RNF2 | C11orf72 | MARF1 |
| CTDP1 | VPS13C | ZNF629 |
| ZNF624 | MBNL3 | PRSS36 |
| GUCY1A2 | CHI3L2 | AMFR |
| MRPL42 | RLN1 | CSNK2A2 |
| STAG1 | TFRC | ZFHX3 |
| ARID4B | RANBP9 | CMC2 |
| GLB1L3 | OSTN | ZCCHC14 |
| ZNF730 | CHST11 | FAHD1 |
| HOOK3 | NDC1 | ABAT |
| CAPS2 | L3HYPDH | ZNF720 |
| PRPF38B | CTDP1 | CYLD |
| FAM114A2 | SPATA6L | ARL2BP |
| CREG1 | C1orf101 | CCL22 |
| MSRB3 | EAF1 | PDP2 |
| PRKD3 | UBE3A | CBFB |
| AFF4 | RNF150 | C16orf70 |
| PDGFRA | TTC32 | SLC7A6 |
| CRKL | FIGF | VPS4A |
| CTTNBP2NL | TRHDE | NFAT5 |
| EIF4E2 | SCRG1 | ATXN1L |
| PHC3 | LOR | DHX38 |
| VPS13B | TMEM255A | ZFP1 |
| CCDC162P | PATE3 | GABARAPL2 |
| ANTXR2 | COPZ1 | CENPN |
| FKBP5 | LRAT | DHX33 |
| DHX38 | GPATCH2L | PITPNM3 |
| FMR1 | BMP8B | ZNF624 |
| SMARCD2 | PDE5A | ULK2 |
| SPTLC2 | PIH1D3 | LYRM9 |
| RPP14 | C15orf40 | PIGS |
| MFAP3L | SMNDC1 | PHF12 |
| ENKUR | DEFB127 | NUFIP2 |
| DNMT3B | WEE2 | RFFL |
| DISP2 | MEX3A | SLFN11 |
| ATXN1 | SLC8A1 | EPOP |
| CTNND1 | RNF175 | MED1 |
| KRT26 | TAL1 | SPOP |
| GNA12 | YWHAG | TRIM25 |
| EXPH5 | HTR2C | SRSF1 |
| SLITRK6 | EFCAB12 | USP32 |
| DNALI1 | ENC1 | BRIP1 |
| ZNF25 | LRRC6 | MED13 |
| GTPBP10 | ZNF226 | CCDC47 |
| USP15 | ING3 | SMARCD2 |
| PDP2 | SERPINB4 | GNA13 |
| LIMD1 | PITHD1 | HELZ |
| PLCXD3 | GYS2 | CDC42EP4 |
| LRAT | SERPINB3 | SUMO2 |
| EYA4 | AGTR2 | GRB2 |
| UNC45B | ELK3 | SRP68 |
| TMPRSS3 | SLC22A15 | PRPSAP1 |
| CAMK1G | CEP19 | MXRA7 |
| GTF2I | NCK1 | JMJD6 |
| LCLAT1 | KRTAP9-7 | WDR81 |
| ZC4H2 | SELL | PAFAH1B1 |
| SH3YL1 | ZNF12 | SENP3 |
| FLT1 | AC012215.1 | KDM6B |
| FOXJ2 | CHRFAM7A | ALDH3A2 |
| VPS4A | CECR6 | TAOK1 |
| BAALC | C12orf4 | RNF135 |
| HNRNPR | PDGFD | SUZ12 |
| DENND2C | AC144568.2 | RHOT1 |
| IQSEC1 | NMRK1 | TADA2A |
| SYN1 | VMA21 | CDK12 |
| OLFML3 | MYCT1 | CALCOCO2 |
| MPP6 | USP44 | LUC7L3 |
| RAB9B | FAM105B | STXBP4 |
| C19orf84 | DISP2 | PCTP |
| RBBP4 | OAS3 | MSI2 |
| ZNF208 | MT-ATP8 | PRR11 |
| KMT5B | SYNCRIP | DHX40 |
| TNFRSF11A | NCAN | RPS6KB1 |
| ADAL | FN3KRP | PPM1D |
| PHIP | IL1A | TANC2 |
| CRISPLD1 | FSHR | DCAF7 |
| AHCYL1 | LMOD3 | PITPNC1 |
| PGAM1 | TIPRL | BIRC5 |
| FBXO48 | TTC21B | FN3KRP |
| ZNF559-ZNF177 | SNAI2 | ENOSF1 |
| HCCS | AL163636.6 | SMAD2 |
| SLC1A2 | PIGM | C18orf32 |
| SLC40A1 | NME1 | RPL17-C18orf32 |
| ZNF544 | CHM | TCF4 |
| NARS2 | MAPK1 | RNF152 |
| ARNTL | ULBP3 | PIGN |
| FFAR2 | DENND3 | BCL2 |
| PAFAH1B2 | POU3F2 | RTTN |
| CAMKK2 | C16orf70 | VAPA |
| PGAM4 | CERS3 | MIB1 |
| SUMO1 | ZNF300 | KIAA1328 |
| YBX1 | LETMD1 | ONECUT2 |
| STOM | ST8SIA2 | NEDD4L |
| CDC42EP4 | FGD4 | CTDP1 |
| NUMB | SLC25A46 | INSR |
| DAB2 | FKBP1A | ELAVL1 |
| ATP11B | TRIM58 | ZNF558 |
| 44260 | PLAC1L | ZNF426 |
| UBE2J1 | C1orf141 | NOTCH3 |
| NFIB | AMFR | ZNF708 |
| SV2B | PDP2 | ZNF208 |
| SOAT1 | SPRYD4 | ZNF676 |
| ZBTB20 | VBP1 | ZNF91 |
| C11orf44 | C10orf35 | ZNF461 |
| PDE3B | AACS | ZNF850 |
| DTWD2 | MRPS25 | ZNF585B |
| CAVIN3 | IRF2 | ZNF180 |
| CPOX | PM20D2 | OPA3 |
| NFASC | PRND | FBXO46 |
| GPX8 | MRPL40 | CARD8 |
| FAM172A | PRRC1 | ZNF649 |
| ZNF354C | FBXO28 | ZNF816-ZNF321P |
| ITGB8 | SEPSECS | ZNF256 |
| ANO1 | PLXDC2 | ZNF329 |
| MRPS7 | SPIN1 | CYP4F3 |
| MAP4 | LRRTM2 | GATAD2A |
| LPAR4 | HS3ST4 | PDCD5 |
| E2F6 | TRIM27 | ZNF302 |
| BTG1 | GPR63 | ZNF345 |
| ZNF527 | KCNMB1 | ZNF527 |
| RMI1 | KCNJ15 | EPN1 |
| SHLD1 | KIF2A | ZNF264 |
| DGKH | GK5 | ZNF549 |
| H2AFV | AC069547.1 | ZNF211 |
| REL | APOLD1 | ZNF551 |
| SLC12A8 | ZNF487 | ZNF586 |
| TANK | NPTN | SH3YL1 |
| MBNL3 | HDGFRP3 | PUM2 |
| NCBP1 | C1orf180 | PRKD3 |
| TMEM268 | OLFML3 | SOS1 |
| SEMA5A | UGT3A2 | COX7A2L |
| WDR20 | ADRA1A | MCFD2 |
| UFL1 | DYNAP | RAB1A |
| PPM1E | SLFN13 | PPP3R1 |
| HLTF | TMEM52B | AC017083.3 |
| RTKN2 | ICA1L | GFPT1 |
| ZFP1 | PEX13 | TIA1 |
| ABCA5 | CEP68 | SFXN5 |
| ZIC1 | CBR1 | GCFC2 |
| KLHL12 | THAP10 | TGFBRAP1 |
| ALG10 | FKBP1C | FHL2 |
| BLCAP | CT62 | IL1A |
| KCTD4 | ARL8B | NCKAP5 |
| ACER3 | CYP3A5 | PRPF40A |
| C3orf14 | C1orf213 | FIGN |
| GON7 | DAOA | SCN9A |
| ADD1 | SLC25A53 | TLK1 |
| IRF2BP2 | FSTL5 | LNPK |
| KTN1 | FAM114A1 | SESTD1 |
| SEM1 | RPL23 | TFPI |
| SLC8A1 | EDEM3 | SLC40A1 |
| ZCCHC14 | PRKCZ | SUMO1 |
| PNN | AP000695.1 | RAPH1 |
| TMEM181 | HLA-DRA | INO80D |
| RBM4 | FAM214A | NDUFS1 |
| BMPR1B | PEX11A | KLF7 |
| P2RX1 | 44266 | ERBB4 |
| CLEC2A | TDRD6 | TUBA4A |
| ANO5 | CRISP3 | ARL4C |
| NME6 | TMEM14E | SOX11 |
| G6PC2 | FAHD1 | RNF144A |
| VAPA | C4orf46 | SELENOI |
| SLF2 | LPAR4 | PPP1CB |
| ZNF705E | NEK7 | LCLAT1 |
| ZNF160 | TTLL11 | SPAST |
| GNA13 | NDNL2 | PPM1B |
| CTNNA3 | PPAPDC2 | ERLEC1 |
| RAPGEF2 | C20orf197 | SPTBN1 |
| POLR2F | RNASE4 | CEP68 |
| TRMT10A | E2F6 | KDM3A |
| GYS2 | CC2D2B | LIPT1 |
| CHM | GSTA4 | C2orf49 |
| JARID2 | MOB1A | EPB41L5 |
| ST8SIA4 | AKAP5 | INHBB |
| GRB2 | LBR | AMER3 |
| JAM2 | SHOC2 | PLEKHB2 |
| AMZ1 | C12orf74 | RIF1 |
| GYPE | FAM76A | TANK |
| ZNF619 | MRPS7 | CSRNP3 |
| WDR41 | CASP10 | AGPS |
| RANBP9 | CISD2 | PNKD |
| KDM5B | FAM151B | EIF4E2 |
| ANO3 | LRRC58 | D2HGDH |
| OPRM1 | SEC22A | TMX4 |
| MYSM1 | URB2 | FLRT3 |
| DTX3L | FBXO3 | CRNKL1 |
| SSX2IP | DCAF4 | RALGAPA2 |
| CSE1L | WARS2 | NOL4L |
| DNAJC27 | BLCAP | BLCAP |
| HTR2C | ITGB8 | CHD6 |
| LVRN | PRDX3 | ZNFX1 |
| UST | PRDM1 | PRELID3B |
| ZFP82 | NME4 | PSMF1 |
| SELENOI | ARL4C | PANK2 |
| HNRNPF | SSX1 | CDS2 |
| SEC14L3 | TMEM116 | MACROD2 |
| TCAF1 | ZNF461 | MGME1 |
| GSS | PRRC2C | XRN2 |
| CLIC4 | SCFD2 | ITCH |
| DKK3 | TMEM65 | EPB41L1 |
| ZMYM2 | ZIC1 | RALGAPB |
| KLHL5 | SPARC | CSE1L |
| BEST3 | CHD6 | GNAS |
| ANKRD34B | REG4 | LSM14B |
| SMC2 | RORA | SS18L1 |
| SLC22A15 | MAN2C1 | NRIP1 |
| ZKSCAN3 | ZNF510 | URB1 |
| PANK2 | ZNF391 | SYNJ1 |
|  | ARL6IP1 | SETD4 |
|  | ZDHHC16 | HLCS |
|  | CAMK4 | CSTB |
|  | CYCS | USP25 |
|  | RBMY1F | SLC5A3 |
|  | GPA33 | MAPK1 |
|  | SLC6A1 | PITPNB |
|  | RBMY1A1 | DDX17 |
|  | AMMECR1 | ST13 |
|  | NMUR1 | POLR3H |
|  | RBMY1J | CRKL |
|  | AP3S2 | UBE2L3 |
|  | RBMY1D | EWSR1 |
|  | ZNF212 | KCTD17 |
|  | TMEM60 | TNRC6B |
|  | ZSCAN25 | SMDT1 |
|  | PLS1 | TAMM41 |
|  | ALMS1 | MRPS25 |
|  | NR3C2 | ANKRD28 |
|  | WDR81 | NEK10 |
|  | RBMY1B | OSBPL10 |
|  | P2RX1 | TMPPE |
|  | AQPEP | SMARCC1 |
|  | CYP19A1 | SFMBT1 |
|  | RBMY1E | C3orf67 |
|  | AP1AR | FRMD4B |
|  | RCC1 | EIF4E3 |
|  | TUBB2A | RYBP |
|  | C8orf37 | DCBLD2 |
|  | PRPS2 | USF3 |
|  | SOX4 | NAA50 |
|  | HIST1H3E | B4GALT4 |
|  | ATG5 | LRRC58 |
|  | FNIP1 | FSTL1 |
|  | PJA2 | KPNA1 |
|  | C10orf118 | PARP9 |
|  | ACTR2 | ZNF148 |
|  | PATE4 | ZXDC |
|  | RBMXL2 | ASTE1 |
|  | AXIN2 | STAG1 |
|  | EPT1 | GK5 |
|  | MAPK7 | COMMD2 |
|  | MMRN1 | KPNA4 |
|  | MAPKAPK2 | MECOM |
|  | TMEM107 | EIF5A2 |
|  | GALNT13 | PLD1 |
|  | ZNF740 | TBL1XR1 |
|  | AP001652.1 | DCUN1D1 |
|  | TRIM22 | DGKG |
|  | SLC38A11 | BDH1 |
|  | FILIP1L | EDEM1 |
|  | LPCAT2 | SLC6A6 |
|  | MAGEB1 | NR2C2 |
|  | DEK | RPL15 |
|  | STMND1 | ZNF619 |
|  | ZNF91 | LIMD1 |
|  | SCAMP1 | CACNA1D |
|  | DHX38 | CCDC66 |
|  | RP11-181C3.1 | RPP14 |
|  | RP11-156E8.1 | HTD2 |
|  | NDST2 | C3orf14 |
|  | TNFSF4 | MITF |
|  | AC107021.1 | ALCAM |
|  | SCN7A | ABHD10 |
|  | PDCL3 | ATP6V1A |
|  | ZKSCAN1 | GTF2E1 |
|  | IL7R | SEC22A |
|  | GOLGA8A | ATP2C1 |
|  | CES5A | NCK1 |
|  | KIAA1143 | ARMC8 |
|  | NAP1L2 | SLC25A36 |
|  | SFMBT1 | PLS1 |
|  | CCDC15 | ZIC1 |
|  | DNMT3B | MME |
|  | GPR135 | NMD3 |
|  | CTNNA3 | FNDC3B |
|  | CXCL14 | SOX2 |
|  | SNAPC1 | ATP11B |
|  | KIAA0430 | LPP |
|  | NDUFS1 | LRCH3 |
|  | SULT1B1 | LMLN |
|  | PAK3 | CTBP1 |
|  | BDP1 | TAPT1 |
|  | CRISPLD1 | LCORL |
|  | BTG2 | PPARGC1A |
|  | SLAMF9 | SEPSECS |
|  | TTC22 | PDS5A |
|  | AKAP2 | FRYL |
|  | NETO1 | USP46 |
|  | TECTB | CLOCK |
|  | ZNF354C | NMU |
|  | SULT1C2 | EPHA5 |
|  | PROK2 | G3BP2 |
|  | HSPA6 | CNOT6L |
|  | NUMB | PAQR3 |
|  | HLF | ANTXR2 |
|  | C12orf36 | RASGEF1B |
|  | ST8SIA6 | HNRNPD |
|  | AL591479.1 | WDFY3 |
|  | NAA16 | TBCK |
|  | RPL37 | ELOVL6 |
|  | LCMT2 | PDE5A |
|  | MEF2A | PCDH18 |
|  | USP38 | SLC7A11 |
|  | MYO6 | ZNF827 |
|  | UNC45B | SH3D19 |
|  | ZFP3 | FGA |
|  | FBXO48 | IRF2 |
|  | LYRM9 | PCGF3 |
|  | GRM5 | SH3BP2 |
|  | ZNF160 | ADD1 |
|  | KLF10 | HTRA3 |
|  | FRS2 | CPEB2 |
|  | EPGN | ZCCHC4 |
|  | HCCS | FAM114A1 |
|  | RAD18 | N4BP2 |
|  | PRIM2 | SLAIN2 |
|  | CENPN | PDGFRA |
|  | SLC5A8 | REST |
|  | TMPRSS11E | CCNG2 |
|  | RSPH3 | AFF1 |
|  | NT5C3A | SMARCAD1 |
|  | GBP5 | BMPR1B |
|  | KRTAP4-12 | CISD2 |
|  | CNIH4 | AIMP1 |
|  | SLC30A9 | SGMS2 |
|  | TXNDC15 | CYP2U1 |
|  | CD160 | HADH |
|  | PTGES | MCUB |
|  | PSMA8 | AP1AR |
|  | MSR1 | ELMOD2 |
|  | RNASE9 | USP38 |
|  | ANKRD49 | ARFIP1 |
|  | LIMD1 | FNIP2 |
|  | NCKAP5 | RAPGEF2 |
|  | SNAPC3 | TENM3 |
|  | TMEM182 | WWC2 |
|  | EDNRB | LPCAT1 |
|  | PDZK1 | C5orf42 |
|  | PRR11 | RICTOR |
|  | ZNF791 | DAB2 |
|  | SFXN5 | PLCXD3 |
|  | ARL2BP | PAIP1 |
|  | TM9SF2 | IL6ST |
|  | ZFYVE16 | MIER3 |
|  | TGM2 | PDE4D |
|  | PAFAH1B2 | SGTB |
|  | ASPH | ENC1 |
|  | WWP1 | WDR41 |
|  | GCC1 | SSBP2 |
|  | ADD2 | TMEM161B |
|  | TNKS | LYSMD3 |
|  | SORD | ARRDC3 |
|  | ULBP1 | FAM172A |
|  | TOX | ELL2 |
|  | GTF2H3 | PJA2 |
|  | DERL1 | TMED7 |
|  | TMOD2 | ZNF608 |
|  | CYP2U1 | FNIP1 |
|  | MRPL42 | IRF1 |
|  | ZBTB37 | AFF4 |
|  | AL138847.1 | KLHL3 |
|  | PTGIS | FAM13B |
|  | GLS | CSNK1A1 |
|  | MAP10 | SPARC |
|  | SEC24A | FAXDC2 |
|  | CCDC163P | SLU7 |
|  | SH3D19 | PANK3 |
|  | FGF14 | KIAA1191 |
|  | NCAPG | CLK4 |
|  | RAB1A | ZFP62 |
|  | 44260 | MTRR |
|  | ERCC4 | OTULIN |
|  | B4GALT3 | SPEF2 |
|  | INSM2 | GPX8 |
|  | TMEM181 | MAP3K1 |
|  | SLC2A2 | KIF2A |
|  | NARS2 | NLN |
|  | CRKL | ERBIN |
|  | AC008443.1 | SERF1A |
|  | WIBG | BDP1 |
|  | MDM2 | MAP1B |
|  | BACE1 | TNPO1 |
|  | ZBTB33 | VCAN |
|  | ERO1L | LNPEP |
|  | MTAP | SLC25A46 |
|  | HDGF | CAMK4 |
|  | SMARCD2 | DCP2 |
|  | NLRP2 | TNFAIP8 |
|  | HNRNPF | PRRC1 |
|  | PIGK | SLC12A2 |
|  | TADA2A | SEC24A |
|  | MATN2 | CAMLG |
|  | HAPLN4 | TXNDC15 |
|  | PALM2-AKAP2 | SMAD5 |
|  | TTC34 | EGR1 |
|  | FNIP2 | MATR3 |
|  | RBM8A | PCDHAC1 |
|  | DIRAS2 | PCDHB3 |
|  | ZNF619 | PCDHB11 |
|  | CD58 | GRPEL2 |
|  | SLC25A36 | PPARGC1B |
|  | VPS4A | SLC26A2 |
|  | DCP2 | HMMR |
|  | SMCO3 | RARS |
|  | AC084121.16 | RANBP17 |
|  | ZNF629 | NEURL1B |
|  | RP11-1118M6.1 | CREBRF |
|  | TUFT1 | ARL10 |
|  | PPP3R2 | RMND5B |
|  | PPM1E | MAML1 |
|  | CYP1A1 | CNOT6 |
|  | MED14 | SSR1 |
|  | YTHDF1 | GFOD1 |
|  | ZNF415 | RANBP9 |
|  | PLA2G2D | ATXN1 |
|  | GRIP1 | DEK |
|  | CTNNA2 | NRM |
|  | TMEM135 | BAG6 |
|  | FBXL22 | SRPK1 |
|  | HTRA1 | ZNF318 |
|  | CACNA1D | HMGCLL1 |
|  | PANK1 | DST |
|  | ARL6 | PHIP |
|  | CDH13 | SYNCRIP |
|  | HIBCH | UBE2J1 |
|  | C6orf141 | EPHA7 |
|  | CYP4F22 | MMS22L |
|  | SETX | SEC63 |
|  | F9 | CDK19 |
|  | SPSB4 | REV3L |
|  | ZKSCAN3 | GOPC |
|  | SMUG1 | MAN1A1 |
|  | C11orf48 | PTPRK |
|  | ITK | LATS1 |
|  | BCAS2 | NUP43 |
|  | PIGS | ZBTB2 |
|  | DUSP18 | AGPAT4 |
|  | RAD54B | PRPF4B |
|  | GSS | ID4 |
|  | SLFN11 | SOX4 |
|  | C17orf102 | ALDH5A1 |
|  | CYP20A1 | ACOT13 |
|  | TAF7L | ZNF391 |
|  | ARL10 | ZKSCAN8 |
|  | VWA5A | ATAT1 |
|  | PSAT1 | ITPR3 |
|  | PARK2 | HMGA1 |
|  | METTL9 | UHRF1BP1 |
|  | MCMDC2 | MAPK14 |
|  | KBTBD6 | BICRAL |
|  | MYO5A | ZNF451 |
|  | PPP1CB | PHF3 |
|  | BCKDHB | SMAP1 |
|  | LRRC38 | SH3BGRL2 |
|  | LHFPL5 | BCKDHB |
|  | GOLGA6L9 | TPBG |
|  | PAN3 | POU3F2 |
|  | LPP | PRDM1 |
|  | C8orf47 | FOXO3 |
|  | PRSS16 | CEP57L1 |
|  | C2orf49 | PDE7B |
|  | MME | ARFGEF3 |
|  | B4GALT6 | AIG1 |
|  | MAPRE1 | UST |
|  | FAM118B | ULBP1 |
|  | PPP3R1 | TMEM181 |
|  | PCMTD1 | QKI |
|  | HHLA2 | GNA12 |
|  | CTD-2370N5.3 | ZNF12 |
|  | KIF3A | SNX13 |
|  | JUND | MACC1 |
|  | BIRC5 | FAM126A |
|  | AC016559.1 | JAZF1 |
|  | KDM1B | PDE1C |
|  | SPTLC2 | INHBA |
|  | PEF1 | GLI3 |
|  | ARNTL | H2AFV |
|  | ABCA4 | PURB |
|  | MAPK13 | HIP1 |
|  | KRAS | YWHAG |
|  | KTN1 | CACNA2D1 |
|  | SLC16A14 | CDK6 |
|  | PXN | PON1 |
|  | GSTM4 | SEM1 |
|  | SUV420H1 | SMURF1 |
|  | DOK6 | ATP5MF |
|  | ZBTB21 | KDM7A |
|  | C17orf67 | TCAF1 |
|  | SPTSSA | ZNF746 |
|  | CTBP1 | KMT2C |
|  | SOCS4 | RAC1 |
|  | GOLGA6L9 | UMAD1 |
|  | SYT10 | TMEM106B |
|  | C20orf196 | CCDC126 |
|  | CA8 | MTURN |
|  | PAX2 | ZNF138 |
|  | FEV | TMEM248 |
|  | THUMPD1 | EIF4H |
|  | GPM6A | RSBN1L |
|  | KIAA1429 | GTPBP10 |
|  | PIK3C2A | CLDN12 |
|  | FGF7 | ANKIB1 |
|  | C15orf38-AP3S2 | VPS50 |
|  | STK32A | COL1A2 |
|  | GAB4 | ZKSCAN5 |
|  | BCOR | ZSCAN25 |
|  | CLVS1 | ZKSCAN1 |
|  | AK4 | MEPCE |
|  | TNFSF15 | CAV2 |
|  | GSG1L | ING3 |
|  | AL033381.1 | METTL2B |
|  | MYOZ3 | UBN2 |
|  | ANO3 | CNTNAP2 |
|  | GNE | REPIN1 |
|  | CALCOCO2 | GALNT11 |
|  | EXOSC2 | MFHAS1 |
|  | LRRD1 | SOX7 |
|  | HAVCR1 | PLAT |
|  | PCGF3 | PCMTD1 |
|  | NT5C2 | RB1CC1 |
|  | TIAM2 | MYBL1 |
|  | GRK4 | VCPIP1 |
|  | SPATA5 | PEX2 |
|  | MUC21 | RUNX1T1 |
|  | CD300C | UQCRB |
|  | NOTCH2NL | ANKRD46 |
|  | GOLGA6L10 | YWHAZ |
|  | PROX1 | RRM2B |
|  | LIN28A | KLF10 |
|  | ONECUT1 | TRPS1 |
|  | YES1 | SAMD12 |
|  | URB1 | DERL1 |
|  | SRBD1 | TMEM65 |
|  | EFNB2 | AGO2 |
|  | TMEM2 | TNKS |
|  | SLAMF8 | SLC7A2 |
|  | TP63 | SLC39A14 |
|  | GTF2H2C | SLC25A37 |
|  | GPR111 | DPYSL2 |
|  | NECAB1 | MAK16 |
|  | CSRNP3 | HOOK3 |
|  | EDIL3 | CHCHD7 |
|  | EYA4 | UBXN2B |
|  | CCDC6 | YTHDF3 |
|  | PRLR | PI15 |
|  | RNASEL | WWP1 |
|  | TOR3A | SDC2 |
|  | ARMCX3 | MATN2 |
|  | POSTN | VPS13B |
|  | LEPROT | DCAF13 |
|  | GNAL | ENY2 |
|  | ZCCHC14 | DENND3 |
|  | UHMK1 | NFIB |
|  | ALKBH1 | BNC2 |
|  | ZNF329 | ELAVL2 |
|  | BLOC1S5 | BAG1 |
|  | NEURL1B | TPM2 |
|  | AKR1B1 | GNE |
|  | MACC1 | TMEM2 |
|  | ADD1 | FRMD3 |
|  | NADK2 | ZNF510 |
|  | KCTD4 | PTBP3 |
|  | TMEM33 | RAB14 |
|  | RIN1 | PTGES |
|  | AHCYL1 | SETX |
|  | FAM169A | ZMYND19 |
|  | NR6A1 | DMRT3 |
|  | FOSL1 | PLPP6 |
|  | CD164 | CDC37L1 |
|  | OXCT1 | SNAPC3 |
|  | FAR1 | MTAP |
|  | YWHAZ | GALT |
|  | PRKCA | CBWD3 |
|  | PUM2 | FAM122A |
|  | MPDU1 | RMI1 |
|  | H2BFM | NTRK2 |
|  | VCAM1 | SPIN1 |
|  | SOX2 | ZNF189 |
|  | RP13-996F3.5 | SMC2 |
|  | APOL6 | ZNF462 |
|  | NUDT7 | PALM2-AKAP2 |
|  | TUBA4A | AKAP2 |
|  | GSPT1 | UGCG |
|  | ALG10B | ZNF618 |
|  | GLRA2 | TMEM268 |
|  | GATAD2B | PAPPA |
|  | ZNF624 | SET |
|  | RPP14 | EXOSC2 |
|  | MXI1 | PNPLA4 |
|  | SMAP1 | RPS6KA3 |
|  | RAB14 | DYNLT3 |
|  | PPL | BCOR |
|  | ANKRD22 | MED14 |
|  | ZNF384 | CASK |
|  | HTR5A-AS1 | WNK3 |
|  | TRIM32 | HDAC8 |
|  | GOLGA6L4 | NAP1L2 |
|  | NDRG1 | ATRX |
|  | GPN2 | CHM |
|  | DAZAP2 | PCDH19 |
|  | PSMD10 | SLC25A53 |
|  | CDHR3 | AMMECR1 |
|  | ABHD3 | THOC2 |
|  | LUZP2 | ZDHHC9 |
|  | AKAP7 | MBNL3 |
|  | CDPF1 | SLITRK4 |
|  | RP11-422N16.3 | HCCS |
|  | CTSE | PRPS2 |
|  | CABP4 | SYAP1 |
|  | SULT1C4 | SYTL5 |
|  | TMEM123 | KDM6A |
|  | ACTR3C | PLP2 |
|  | EPB41L5 | CLCN5 |
|  | ELTD1 | AR |
|  | HLTF | CHIC1 |
|  | ILDR1 | ARMCX3 |
|  | STRADA | FAM199X |
|  | PABPC5 | ZBTB33 |
|  | MUC4 | XIAP |
|  | IL20RB | PHF6 |
|  | ZBTB2 | ZNF449 |
|  | PLAA | FMR1 |
|  | ZDHHC9 | MTMR1 |
|  | SHC1 | VMA21 |
|  | PCDH11Y | ABCD1 |
|  | NYAP2 | GDI1 |
|  | ARFIP1 |  |
|  | EED |  |
|  | FAM126A |  |
|  | LAMP3 |  |
|  | C11orf57 |  |
|  | CLEC11A |  |
|  | COX11 |  |
|  | GNA13 |  |
|  | MED18 |  |
|  | EMX2 |  |
|  | TBX20 |  |
|  | DRGX |  |
|  | MSTO1 |  |
|  | ZNF667 |  |
|  | C18orf54 |  |
|  | PPP1R3G |  |
|  | IL24 |  |
|  | FAM122C |  |
|  | WBP1L |  |
|  | SYNDIG1L |  |
|  | ZNF606 |  |
|  | GCLM |  |
|  | KDM3A |  |
|  | RNF135 |  |
|  | SS18L1 |  |
|  | SOX7 |  |
|  | GDPD4 |  |
|  | WNT2B |  |
|  | EYS |  |
|  | MAGEB4 |  |
|  | GOLGA8G |  |
|  | TMEM86A |  |
|  | C7orf41 |  |
|  | TNFRSF11A |  |
|  | ZNF107 |  |
|  | LPCAT1 |  |
|  | TMEM59L |  |
|  | DGKH |  |
|  | GPATCH2 |  |
|  | BLOC1S3 |  |
|  | C2CD2 |  |
|  | ARRDC3 |  |
|  | ZNF618 |  |
|  | RASSF4 |  |
|  | FBXO34 |  |
|  | IPP |  |
|  | GK |  |
|  | NCBP1 |  |
|  | ADAM28 |  |
|  | FHL1 |  |
|  | ACAA2 |  |
|  | DZIP1 |  |
|  | MSRA |  |
|  | FAM161A |  |
|  | RAB15 |  |
|  | RBM7 |  |
|  | RXFP1 |  |
|  | HIST2H2BE |  |
|  | TMEM236 |  |
|  | ZNF146 |  |
|  | TMEM236 |  |
|  | SETD6 |  |
|  | TFAP2C |  |
|  | LGI1 |  |
|  | PLEKHG1 |  |
|  | C1QL3 |  |
|  | PLP2 |  |
|  | ACBD3 |  |
|  | FAM199X |  |
|  | MAP2K1 |  |
|  | DACT2 |  |
|  | PPARGC1B |  |
|  | ADIPOR1 |  |
|  | IGF1 |  |
|  | MAK16 |  |
|  | FOXJ2 |  |
|  | POLD3 |  |
|  | MTMR1 |  |
|  | SLITRK6 |  |
|  | FGD5 |  |
|  | SLC16A4 |  |
|  | FAM117B |  |
|  | SCN9A |  |
|  | NME6 |  |
|  | HDGFL1 |  |
|  | DCLRE1B |  |
|  | BICC1 |  |
|  | KLHL6 |  |
|  | ECT2L |  |
|  | DFFA |  |
|  | BAALC |  |
|  | SRPK1 |  |
|  | ANKRD45 |  |
|  | PEX26 |  |
|  | SLITRK4 |  |
|  | FAM129C |  |
|  | COPS4 |  |
|  | UBQLN4 |  |
|  | GCFC2 |  |
|  | DUSP16 |  |
|  | PPAP2B |  |
|  | CDK5R2 |  |
|  | DCHS2 |  |
|  | RRP36 |  |
|  | DCUN1D1 |  |
|  | CDKL1 |  |
|  | KAL1 |  |
|  | CASC4 |  |
|  | ASRGL1 |  |
|  | HPCAL4 |  |
|  | TMEM167B |  |
|  | MMGT1 |  |
|  | B4GALT4 |  |
|  | ATP13A4 |  |
|  | SLC4A4 |  |
|  | SSR1 |  |
|  | XPR1 |  |
|  | FKTN |  |
|  | AC010441.1 |  |
|  | SGK1 |  |
|  | ERMN |  |
|  | ZNF320 |  |
|  | WNK3 |  |
|  | NAA40 |  |
|  | KCNG1 |  |
|  | SLC7A6 |  |
|  | DESI2 |  |
|  | PRKG2 |  |
|  | FUNDC2 |  |
|  | ABCD2 |  |
|  | GATM |  |
|  | SYNDIG1 |  |
|  | HTR1F |  |
|  | NUP62CL |  |
|  | SPRY3 |  |
|  | CXCL5 |  |
|  | GPR15 |  |
|  | PPARGC1A |  |
|  | BTLA |  |
|  | DCAF4L1 |  |
|  | ITPR3 |  |
|  | EID1 |  |
|  | TRMT10A |  |
|  | AGFG1 |  |
|  | GDF11 |  |
|  | PRRT4 |  |
|  | N4BP2 |  |
|  | ZNF549 |  |
|  | ACAD11 |  |
|  | JAM2 |  |
|  | STRIP1 |  |
|  | CCSAP |  |
|  | HIP1 |  |
|  | WDR47 |  |
|  | VTI1B |  |
|  | METTL7A |  |
|  | RHD |  |
|  | CHRNA7 |  |
|  | ANKRD34C |  |
|  | SAV1 |  |
|  | STK32B |  |
|  | PAQR8 |  |
|  | CNTNAP2 |  |
|  | AFF4 |  |
|  | UBE2G1 |  |
|  | IL17D |  |
|  | EPSTI1 |  |
|  | FOXN2 |  |
|  | KCNK13 |  |
|  | CTNND1 |  |
|  | GABRG1 |  |
|  | AP005482.1 |  |
|  | EFCAB6 |  |
|  | CAMKK2 |  |
|  | IKZF5 |  |
|  | DAP |  |
|  | THBD |  |
|  | EIF4H |  |
|  | GATAD2A |  |
|  | GLIPR1 |  |
|  | KPNA6 |  |
|  | DGKI |  |
|  | AL031666.2 |  |
|  | C7 |  |
|  | MYZAP |  |
|  | DPYSL2 |  |
|  | C1orf189 |  |
|  | C12orf79 |  |
|  | COL1A2 |  |
|  | ACER2 |  |
|  | WWC2 |  |
|  | STAU2 |  |
|  | ZBED1 |  |
|  | SH3RF2 |  |
|  | TNFSF10 |  |
|  | KIAA1024 |  |
|  | NPHP3 |  |
|  | ZKSCAN5 |  |
|  | PPAT |  |
|  | SRP72 |  |
|  | ADAMTS9 |  |
|  | MNT |  |
|  | CDKN2B |  |
|  | SLCO2B1 |  |
|  | ZNF706 |  |
|  | NUDT4 |  |
|  | WDR70 |  |
|  | BNIP3 |  |
|  | PAWR |  |
|  | CD59 |  |
|  | COX20 |  |
|  | ZNF341 |  |
|  | GOLGA8F |  |
|  | PLEKHH2 |  |
|  | BMP2 |  |
|  | ATP5E |  |
|  | DMRT3 |  |
|  | DTWD2 |  |
|  | HAVCR2 |  |
|  | SEMA3E |  |
|  | FAM196B |  |
|  | SERINC5 |  |
|  | TMA16 |  |
|  | ZIC5 |  |
|  | DNAJC27 |  |
|  | C10orf88 |  |
|  | PAX9 |  |
|  | CSNK1A1 |  |
|  | DDHD2 |  |
|  | GABRQ |  |
|  | GRM7 |  |
|  | ASB15 |  |
|  | ZNF561 |  |
|  | SLC19A1 |  |
|  | BCL7A |  |
|  | PTPRE |  |
|  | ZCCHC7 |  |
|  | ZNF780B |  |
|  | CYP27C1 |  |
|  | WAPAL |  |
|  | CYP51A1 |  |
|  | ONECUT3 |  |
|  | ZBTB4 |  |
|  | UQCRFS1 |  |
|  | KLHL9 |  |
|  | UGT1A6 |  |
|  | UGT1A8 |  |
|  | UGT1A5 |  |
|  | LPIN2 |  |
|  | UGT1A9 |  |
|  | UGT1A3 |  |
|  | GOLGA6C |  |
|  | GOLGA6D |  |
|  | UGT1A4 |  |
|  | UGT1A7 |  |
|  | FSTL1 |  |
|  | ANK3 |  |
|  | RHOQ |  |
|  | CASK |  |
|  | RAB3IP |  |
|  | PDLIM5 |  |
|  | ELL2 |  |
|  | TAS2R10 |  |
|  | IPMK |  |
|  | FKBP5 |  |
|  | CDHR1 |  |
|  | UGT1A10 |  |
|  | MYO5B |  |
|  | PINX1 |  |
|  | UGT1A1 |  |
|  | AHR |  |
|  | KPNA4 |  |
|  | EDEM1 |  |
|  | REL |  |
|  | CD226 |  |
|  | C9orf91 |  |
|  | EFCAB1 |  |
|  | HS3ST3A1 |  |
|  | JAZF1 |  |
|  | YOD1 |  |
|  | TTBK2 |  |
|  | MAGOHB |  |
|  | TCTN3 |  |
|  | LIN52 |  |
|  | UBL4B |  |
|  | SETBP1 |  |
|  | IREB2 |  |
|  | ELF5 |  |
|  | GOLT1B |  |
|  | MED1 |  |
|  | FMR1 |  |
|  | PITPNB |  |
|  | ALDH18A1 |  |
|  | MIXL1 |  |
|  | STARD7 |  |
|  | ALG10 |  |
|  | STXBP4 |  |
|  | MTIF2 |  |
|  | BAK1 |  |
|  | ITGB6 |  |
|  | ZNF302 |  |
|  | ZDHHC23 |  |
|  | TOX2 |  |
|  | DENND2C |  |
|  | ALKBH5 |  |
|  | NPR1 |  |
|  | COL9A1 |  |
|  | BDH1 |  |
|  | ZNF264 |  |
|  | ART4 |  |
|  | PRKCH |  |
|  | CASC3 |  |
|  | GOLGA8J |  |
|  | KIAA1467 |  |
|  | GFRA3 |  |
|  | KMO |  |
|  | SRSF3 |  |
|  | ZNF449 |  |
|  | HIST1H2AK |  |
|  | ANGPT1 |  |
|  | SERP1 |  |
|  | TMEM106B |  |
|  | PDE3B |  |
|  | LYSMD3 |  |
|  | LMBRD2 |  |
|  | HELZ |  |
|  | STXBP5L |  |
|  | SLC1A2 |  |
|  | UTP14C |  |
|  | TECTA |  |
|  | SLC41A1 |  |
|  | RNF187 |  |
|  | VCL |  |
|  | GPR64 |  |
|  | SBSPON |  |
|  | NCR3LG1 |  |
|  | CAAP1 |  |
|  | TRABD2A |  |
|  | CHMP1B |  |
|  | DDX3Y |  |
|  | GOLGA8H |  |
|  | KCNQ5 |  |
|  | KIAA1257 |  |
|  | REPIN1 |  |
|  | CAPN12 |  |
|  | ANXA4 |  |
|  | MLLT11 |  |
|  | SCG3 |  |
|  | VEZF1 |  |
|  | CCDC68 |  |
|  | PTPLAD2 |  |
|  | RASSF1 |  |
|  | HES5 |  |
|  | LCT |  |
|  | GLCE |  |
|  | GHITM |  |
|  | KLHL41 |  |
|  | VTI1A |  |
|  | DIAPH3 |  |
|  | DOK1 |  |
|  | LCP2 |  |
|  | LRRC63 |  |
|  | TBK1 |  |
|  | ZNF705D |  |
|  | ZNF705B |  |
|  | EXPH5 |  |
|  | ANKRD13C |  |
|  | PHC3 |  |
|  | FRMD6 |  |
|  | TMEM245 |  |
|  | OLR1 |  |
|  | APAF1 |  |
|  | RARRES3 |  |
|  | C10orf67 |  |
|  | PARP15 |  |
|  | NHLH1 |  |
|  | JAKMIP3 |  |
|  | GCSAML |  |
|  | TMEM196 |  |
|  | NUDT12 |  |
|  | TSC22D1 |  |
|  | MFAP3L |  |
|  | TMEM56 |  |
|  | HN1 |  |
|  | NADK |  |
|  | SENP5 |  |
|  | C5orf51 |  |
|  | CAPN14 |  |
|  | HRH1 |  |
|  | RCOR1 |  |
|  | SAMD12 |  |
|  | AJAP1 |  |
|  | TMEM239 |  |
|  | CAV2 |  |
|  | SYTL5 |  |
|  | PTPRJ |  |
|  | CYP4F3 |  |
|  | FLRT3 |  |
|  | ZNF705G |  |
|  | ZNF471 |  |
|  | CCRN4L |  |
|  | CBLN1 |  |
|  | PHF12 |  |
|  | VSIG10L |  |
|  | UBXN10 |  |
|  | TAOK1 |  |
|  | GOSR2 |  |
|  | ORMDL3 |  |
|  | BRMS1L |  |
|  | TRMT44 |  |
|  | AL603965.1 |  |
|  | AL591684.1 |  |
|  | NANOG |  |
|  | KCTD5 |  |
|  | TMEM41B |  |
|  | OSBP2 |  |
|  | ZBTB8A |  |
|  | BMPR1B |  |
|  | GUCY1A2 |  |
|  | ZNF292 |  |
|  | MAB21L1 |  |
|  | YPEL1 |  |
|  | HIST1H2AH |  |
|  | MGAM |  |
|  | CXCL9 |  |
|  | CLCA2 |  |
|  | ZNF705A |  |
|  | GOLGA8I |  |
|  | ARL5B |  |
|  | TP73 |  |
|  | HHIP |  |
|  | ST6GAL1 |  |
|  | FSD1L |  |
|  | ZNF562 |  |
|  | ZFP36L1 |  |
|  | SLC25A24 |  |
|  | DNAH11 |  |
|  | ZNF616 |  |
|  | CAV1 |  |
|  | FBXO36 |  |
|  | TCF12 |  |
|  | PDK1 |  |
|  | FYTTD1 |  |
|  | COMMD2 |  |
|  | ELAVL2 |  |
|  | SLC14A2 |  |
|  | ZNF852 |  |
|  | PLCXD2 |  |
|  | ZNF721 |  |
|  | LMX1A |  |
|  | PDPN |  |
|  | COA5 |  |
|  | PURG |  |
|  | GRIK4 |  |
|  | MAX |  |
|  | PPP4R1 |  |
|  | ELK4 |  |
|  | CHURC1 |  |
|  | ADAMTSL3 |  |
|  | TIFA |  |
|  | VAPB |  |
|  | FABP4 |  |
|  | KIAA1715 |  |
|  | KCTD7 |  |
|  | PHLDA3 |  |
|  | MAATS1 |  |
|  | FMOD |  |
|  | MYRIP |  |
|  | AMER3 |  |
|  | LETM1 |  |
|  | G3BP2 |  |
|  | TOX4 |  |
|  | CBFB |  |
|  | SMC2 |  |
|  | HSPA4 |  |
|  | ADAM12 |  |
|  | FSBP |  |
|  | MED13 |  |
|  | CCND1 |  |
|  | RAB32 |  |
|  | C1orf109 |  |
|  | MINPP1 |  |
|  | NCKAP1L |  |
|  | LMAN1 |  |
|  | GPR160 |  |
|  | RAPGEF2 |  |
|  | SUCNR1 |  |
|  | ONECUT2 |  |
|  | HTR2A |  |
|  | C1orf52 |  |
|  | ATP6V1C1 |  |
|  | BHLHE22 |  |
|  | EGR1 |  |
|  | GPR180 |  |
|  | PAPPA |  |
|  | CREBZF |  |
|  | CSGALNACT2 |  |
|  | CEP170 |  |
|  | ARHGAP42 |  |
|  | ADAL |  |
|  | RUNX2 |  |
|  | PAX1 |  |
|  | HGF |  |
|  | PAX3 |  |
|  | MSTN |  |
|  | GAD2 |  |
|  | RARS2 |  |
|  | SNX21 |  |
|  | TP53INP1 |  |
|  | MEPCE |  |
|  | PCYOX1 |  |
|  | RFESD |  |
|  | FKBP14 |  |
|  | C6orf89 |  |
|  | NUS1 |  |
|  | C17orf77 |  |
|  | GOLGA8O |  |
|  | SERPINB9 |  |
|  | GOLGA8R |  |
|  | TSPYL5 |  |
|  | ARL5C |  |
|  | KAT7 |  |
|  | CHD5 |  |
|  | SGK223 |  |
|  | HLCS |  |
|  | ZFP69B |  |
|  | RSBN1 |  |
|  | SLC30A6 |  |
|  | CCND2 |  |
|  | AL354898.1 |  |
|  | SLC6A12 |  |
|  | MYO7A |  |
|  | TMCO1 |  |
|  | QSER1 |  |
|  | LHX4 |  |
|  | GREM1 |  |
|  | FBXO25 |  |
|  | CCDC176 |  |
|  | COA1 |  |
|  | FITM2 |  |
|  | XXYLT1 |  |
|  | TASP1 |  |
|  | MYF6 |  |
|  | LRRC4 |  |
|  | GOLGA8N |  |
|  | NTRK2 |  |
|  | PDGFC |  |
|  | PLD5 |  |
|  | EXOC8 |  |
|  | ARL11 |  |
|  | ZNF785 |  |
|  | PDE4A |  |
|  | ZMPSTE24 |  |
|  | HSPBAP1 |  |
|  | BMP2K |  |
|  | MOB4 |  |
|  | SLC25A37 |  |
|  | COLEC10 |  |
|  | KLHL5 |  |
|  | CLEC5A |  |
|  | SLC26A2 |  |
|  | ZNF776 |  |
|  | ARID4B |  |
|  | PPP2R5E |  |
|  | MAT2B |  |
|  | ZNF649 |  |
|  | FBXO46 |  |
|  | PDE4D |  |
|  | SDHAF2 |  |
|  | NT5DC3 |  |
|  | CDC42BPA |  |
|  | ATXN7 |  |
|  | CADM2 |  |
|  | MCFD2 |  |
|  | ERLEC1 |  |
|  | ANAPC16 |  |
|  | FAM178A |  |
|  | DR1 |  |
|  | CHRNA5 |  |
|  | GOLGA8K |  |
|  | BMPR1A |  |
|  | RNF215 |  |
|  | SDS |  |
|  | TEAD1 |  |
|  | TOR1B |  |
|  | PCDH19 |  |
|  | ZNF138 |  |
|  | OR1L8 |  |
|  | KANK4 |  |
|  | SDC2 |  |
|  | VPS41 |  |
|  | MARK4 |  |
|  | CDKN2AIP |  |
|  | SPRYD3 |  |
|  | ABCC4 |  |
|  | B3GNT5 |  |
|  | FAM163A |  |
|  | SYN1 |  |
|  | GOLGA8M |  |
|  | C16orf72 |  |
|  | PID1 |  |
|  | MSRB3 |  |
|  | PPCS |  |
|  | FBXO22 |  |
|  | FAF2 |  |
|  | ZFP1 |  |
|  | MPP6 |  |
|  | MTMR9 |  |
|  | CALCR |  |
|  | CLEC3A |  |
|  | SOGA2 |  |
|  | ADORA1 |  |
|  | DUSP10 |  |
|  | MCOLN2 |  |
|  | PGM1 |  |
|  | OXTR |  |
|  | MFSD8 |  |
|  | PGAP1 |  |
|  | SYNJ2BP |  |
|  | KLHL28 |  |
|  | ANKRD62 |  |
|  | KAT2A |  |
|  | SERPINA11 |  |
|  | CPNE3 |  |
|  | PLEKHB2 |  |
|  | RCC2 |  |
|  | TBX3 |  |
|  | MEX3C |  |
|  | CCDC85A |  |
|  | SNAP25 |  |
|  | STAG1 |  |
|  | SMAD5 |  |
|  | LTB4R |  |
|  | NHLRC3 |  |
|  | GMPS |  |
|  | FGD6 |  |
|  | ADORA3 |  |
|  | MKX |  |
|  | SLC16A6 |  |
|  | RB1CC1 |  |
|  | SASS6 |  |
|  | ZNF583 |  |
|  | RAP2A |  |
|  | TRPM4 |  |
|  | SFXN1 |  |
|  | CDK19 |  |
|  | PCDH10 |  |
|  | CDK6 |  |
|  | TGS1 |  |
|  | OSBPL10 |  |
|  | FCHSD2 |  |
|  | SRP9 |  |
|  | MED7 |  |
|  | EMILIN3 |  |
|  | MAPK14 |  |
|  | LIPA |  |
|  | SLC12A8 |  |
|  | DBX2 |  |
|  | EPHA5 |  |
|  | DNALI1 |  |
|  | PIWIL3 |  |
|  | SLC36A4 |  |
|  | UNC5D |  |
|  | ZC3HAV1L |  |
|  | FBXW11 |  |
|  | PEG10 |  |
|  | ZNF699 |  |
|  | ARRDC4 |  |
|  | PDGFRA |  |
|  | 44441 |  |
|  | TMPRSS12 |  |
|  | TPMT |  |
|  | SVIP |  |
|  | NR1D2 |  |
|  | SV2B |  |
|  | CIAO1 |  |
|  | ARHGAP28 |  |
|  | TRIM2 |  |
|  | SLC4A7 |  |
|  | ZNF878 |  |
|  | SCNN1G |  |
|  | COL19A1 |  |
|  | KRT38 |  |
|  | PRDX6 |  |
|  | DCTN6 |  |
|  | DAAM1 |  |
|  | RABL5 |  |
|  | TIMM8A |  |
|  | SOAT1 |  |
|  | ATF7IP |  |
|  | ST6GALNAC3 |  |
|  | ANKS6 |  |
|  | CCDC36 |  |
|  | B3GALT5 |  |
|  | PVRL3 |  |
|  | CHIC1 |  |
|  | NDE1 |  |
|  | NXPH3 |  |
|  | E2F5 |  |
|  | IFIT2 |  |
|  | ZEB1 |  |
|  | SMIM13 |  |
|  | KCNH5 |  |
|  | ZMAT3 |  |
|  | THRB |  |
|  | FOXD2 |  |
|  | ABTB2 |  |
|  | PLEKHA2 |  |
|  | BBX |  |
|  | BCLAF1 |  |
|  | ZFAND3 |  |
|  | CDH6 |  |
|  | PAFAH1B1 |  |
|  | MDFIC |  |
|  | CAMSAP2 |  |
|  | GPR137C |  |
|  | CALCRL |  |
|  | LRRTM3 |  |
|  | C17orf51 |  |
|  | RNF111 |  |
|  | ZCCHC8 |  |
|  | CNOT1 |  |
|  | NAA35 |  |
|  | ARF3 |  |
|  | FNBP1L |  |
|  | PIK3R1 |  |
|  | PHF21A |  |
|  | CTDSP2 |  |
|  | ZNF483 |  |
|  | CYP4A11 |  |
|  | TIGD4 |  |
|  | TSPYL4 |  |
|  | RET |  |
|  | UBE2J1 |  |
|  | TMEM132D |  |
|  | SEMA5A |  |
|  | MECOM |  |
|  | PHIP |  |
|  | CARHSP1 |  |
|  | SLC25A17 |  |
|  | KLK13 |  |
|  | PNPLA8 |  |
|  | FANCB |  |
|  | TMEM43 |  |
|  | RDX |  |
|  | LIN28B |  |
|  | PWWP2A |  |
|  | ERBB3 |  |
|  | ZNF321P |  |
|  | ITGA8 |  |
|  | SERBP1 |  |
|  | GREM2 |  |
|  | PNPLA4 |  |
|  | KLRC4 |  |
|  | CBX5 |  |
|  | WDR36 |  |
|  | CSE1L |  |
|  | RCHY1 |  |
|  | TMEM68 |  |
|  | RTKN2 |  |
|  | ATPAF1 |  |
|  | ZC3H12C |  |
|  | RRAGC |  |
|  | CCT2 |  |
|  | ZNF28 |  |
|  | HSF5 |  |
|  | EID2B |  |
|  | LONP2 |  |
|  | SLC5A1 |  |
|  | CAPRIN1 |  |
|  | ATG4C |  |
|  | SOX11 |  |
|  | ZNF268 |  |
|  | HMCES |  |
|  | ZNF556 |  |
|  | TMCC3 |  |
|  | SLC12A2 |  |
|  | COIL |  |
|  | NAALADL2 |  |
|  | TNPO1 |  |
|  | ARHGAP40 |  |
|  | TYSND1 |  |
|  | MACROD2 |  |
|  | C2orf69 |  |
|  | STON1 |  |
|  | PURB |  |
|  | WSB1 |  |
|  | GBA |  |
|  | CNP |  |
|  | CASR |  |
|  | C1orf204 |  |
|  | HS3ST3B1 |  |
|  | GLTSCR1L |  |
|  | VPS13B |  |
|  | ST13 |  |
|  | MYLK |  |
|  | NRP1 |  |
|  | TGM4 |  |
|  | SLC40A1 |  |
|  | ZNF641 |  |
|  | TIMP4 |  |
|  | BNC2 |  |
|  | RBM33 |  |
|  | USP1 |  |
|  | MKLN1 |  |
|  | PSKH1 |  |
|  | C6orf223 |  |
|  | ORC2 |  |
|  | GALNT2 |  |
|  | PELO |  |
|  | INPP5F |  |
|  | RGR |  |
|  | ATP10B |  |
|  | ANKRD27 |  |
|  | POU2F1 |  |
|  | ANKRD13B |  |
|  | TRIM65 |  |
|  | HSPE1-MOB4 |  |
|  | KIF24 |  |
|  | RBM25 |  |
|  | MEMO1 |  |
|  | PTPRF |  |
|  | LZIC |  |
|  | KLHL42 |  |
|  | GLI3 |  |
|  | ARL5A |  |
|  | FHL5 |  |
|  | RAB30 |  |
|  | IKBKG |  |
|  | C3orf14 |  |
|  | PDPK1 |  |
|  | PDE10A |  |
|  | SDF2 |  |
|  | AGO1 |  |
|  | ZRSR1 |  |
|  | TIMM22 |  |
|  | KCND3 |  |
|  | BCAS1 |  |
|  | EFHD2 |  |
|  | PVRL4 |  |
|  | MDGA2 |  |
|  | FAM83F |  |
|  | PITPNM3 |  |
|  | ZFY |  |
|  | PPP2R5A |  |
|  | CNTN5 |  |
|  | NIP7 |  |
|  | FAHD2A |  |
|  | EPHA3 |  |
|  | HIF1A |  |
|  | POLK |  |
|  | GAREM |  |
|  | USP46 |  |
|  | NPY1R |  |
|  | AIMP1 |  |
|  | C1QTNF7 |  |
|  | DDAH1 |  |
|  | ALCAM |  |
|  | VGLL4 |  |
|  | PLD1 |  |
|  | NRG1 |  |
|  | SLC39A14 |  |
|  | TRIM25 |  |
|  | LOX |  |
|  | POLR2F |  |
|  | RMND5A |  |
|  | MGAT2 |  |
|  | SAP30 |  |
|  | CCDC80 |  |
|  | XPO7 |  |
|  | KDSR |  |
|  | USP6 |  |
|  | ZNF638 |  |
|  | EPB42 |  |
|  | EPN1 |  |
|  | ARCN1 |  |
|  | FAM120AOS |  |
|  | JHDM1D |  |
|  | XRN1 |  |
|  | ZC3H10 |  |
|  | TAPT1 |  |
|  | SLC38A1 |  |
|  | TPRG1L |  |
|  | TEP1 |  |
|  | DGKG |  |
|  | GLYR1 |  |
|  | TBC1D26 |  |
|  | TNPO3 |  |
|  | RNF152 |  |
|  | ZNF41 |  |
|  | TSC22D2 |  |
|  | NAV2 |  |
|  | CDK12 |  |
|  | ZNF546 |  |
|  | FAM219B |  |
|  | ERN1 |  |
|  | TIRAP |  |
|  | RC3H1 |  |
|  | ATP2B1 |  |
|  | OSTM1 |  |
|  | ANO1 |  |
|  | XIAP |  |
|  | ZNF224 |  |
|  | MAN1A2 |  |
|  | RGS18 |  |
|  | MKI67 |  |
|  | SERPINB8 |  |
|  | FAM155B |  |
|  | PAQR9 |  |
|  | ADIPOQ |  |
|  | TEKT1 |  |
|  | TMEM63C |  |
|  | GOPC |  |
|  | FAM129A |  |
|  | TBX18 |  |
|  | LGI2 |  |
|  | NR2C2 |  |
|  | LANCL3 |  |
|  | GATAD1 |  |
|  | FAM110B |  |
|  | PRKD3 |  |
|  | TBC1D5 |  |
|  | JDP2 |  |
|  | DNAJA2 |  |
|  | PKIA |  |
|  | DIAPH2 |  |
|  | VAMP1 |  |
|  | SAMD7 |  |
|  | MMAA |  |
|  | DCTN5 |  |
|  | ENTPD1 |  |
|  | FAM212B |  |
|  | EEA1 |  |
|  | NR2C1 |  |
|  | RP11-724O16.1 |  |
|  | SRPK2 |  |
|  | LRRC14 |  |
|  | GXYLT2 |  |
|  | CERK |  |
|  | TIGD6 |  |
|  | EPS8L2 |  |
|  | ZFP62 |  |
|  | GPC5 |  |
|  | TPK1 |  |
|  | RHO |  |
|  | SHISA5 |  |
|  | ASAP1 |  |
|  | TRAF6 |  |
|  | ZNF501 |  |
|  | TPRA1 |  |
|  | NKD1 |  |
|  | CYLD |  |
|  | RGS4 |  |
|  | FAM174B |  |
|  | FZD3 |  |
|  | TMED8 |  |
|  | ZNF462 |  |
|  | FAM168A |  |
|  | FMNL2 |  |
|  | RORB |  |
|  | GBP4 |  |
|  | TLR7 |  |
|  | CHCHD7 |  |
|  | JPH2 |  |
|  | PRDM6 |  |
|  | DCLK1 |  |
|  | DUSP4 |  |
|  | MYH10 |  |
|  | TMEM200C |  |
|  | GTF3C4 |  |
|  | MATR3 |  |
|  | VAPA |  |
|  | ENPP1 |  |
|  | FGF1 |  |
|  | FAM71F2 |  |
|  | SNX11 |  |
|  | SLC7A2 |  |
|  | CLEC2D |  |
|  | OTX2 |  |
|  | TMEM229B |  |
|  | SLC35D1 |  |
|  | POLR3D |  |
|  | MTM1 |  |
|  | RABGAP1L |  |
|  | CDC26 |  |
|  | BHLHA15 |  |
|  | SNTB2 |  |
|  | METTL14 |  |
|  | ATP5S |  |
|  | PLXNA4 |  |
|  | SGCD |  |
|  | ELOVL7 |  |
|  | PRKCB |  |
|  | CAP1 |  |
|  | DLGAP2 |  |
|  | NOS1 |  |
|  | SLC35E2B |  |
|  | ASXL3 |  |
|  | KCNJ6 |  |
|  | MID1 |  |
|  | UNC13A |  |
|  | KIF26B |  |
|  | FAT3 |  |
|  | TENM3 |  |
|  | ANK2 |  |
|  | RTTN |  |
|  | ABCA1 |  |
|  | CPN2 |  |
|  | SIAE |  |
|  | ZNF280B |  |
|  | MYLK4 |  |
|  | SPTLC1 |  |
|  | TMPPE |  |
|  | CHTOP |  |
|  | SDAD1 |  |
|  | MIB1 |  |
|  | RALGAPA2 |  |
|  | PDCD4 |  |
|  | ARHGAP12 |  |
|  | CERS6 |  |
|  | PRKD1 |  |
|  | ABCF3 |  |
|  | CLNK |  |
|  | LGR4 |  |
|  | HIST1H2AE |  |
|  | ERC1 |  |
|  | IMPACT |  |
|  | CDC37L1 |  |
|  | INTS6 |  |
|  | AAK1 |  |
|  | PGBD4 |  |
|  | TRPC6 |  |
|  | AKT3 |  |
|  | ABL2 |  |
|  | ME3 |  |
|  | RRM2 |  |
|  | PPM1F |  |
|  | OTUD7B |  |
|  | ANO5 |  |
|  | WDR41 |  |
|  | ACSBG1 |  |
|  | SHOX |  |
|  | PLA2G4D |  |
|  | SLC35E4 |  |
|  | CALN1 |  |
|  | SAMD8 |  |
|  | ADAMTS5 |  |
|  | DPP8 |  |
|  | GPR141 |  |
|  | ATP1A2 |  |
|  | TRMT10B |  |
|  | PRKAA2 |  |
|  | FBXO27 |  |
|  | HOXA11 |  |
|  | C11orf30 |  |
|  | HS3ST1 |  |
|  | ANKRD33B |  |
|  | SAMD4A |  |
|  | BRD7 |  |
|  | MFN2 |  |
|  | UHRF1BP1 |  |
|  | PDHX |  |
|  | ZNF850 |  |
|  | CLIC5 |  |
|  | SESTD1 |  |
|  | ZNF665 |  |
|  | PROSER1 |  |
|  | KRT80 |  |
|  | DYNLL2 |  |
|  | ARPP19 |  |
|  | SLC16A10 |  |
|  | ATG2B |  |
|  | ADRB3 |  |
|  | MPLKIP |  |
|  | TMED5 |  |
|  | TGFBRAP1 |  |
|  | RIF1 |  |
|  | SUN1 |  |
|  | GPR156 |  |
|  | SIM2 |  |
|  | COG1 |  |
|  | SLC6A6 |  |
|  | ERBB4 |  |
|  | SKIL |  |
|  | ZNF514 |  |
|  | IRAK3 |  |
|  | DUSP3 |  |
|  | FAM182B |  |
|  | TOMM22 |  |
|  | TMEM180 |  |
|  | UTP6 |  |
|  | ATXN1 |  |
|  | RPS6KA3 |  |
|  | ZNF787 |  |
|  | PLAG1 |  |
|  | PDK3 |  |
|  | NFIB |  |
|  | NEDD4L |  |
|  | NAV1 |  |
|  | RAB3GAP2 |  |
|  | PAIP2B |  |
|  | CTBS |  |
|  | C20orf112 |  |
|  | C2orf68 |  |
|  | SP3 |  |
|  | SLC10A6 |  |
|  | LIPH |  |
|  | LCOR |  |
|  | KIAA1755 |  |
|  | COL18A1 |  |
|  | HIST1H2BD |  |
|  | UBR2 |  |
|  | FAM63B |  |
|  | MAVS |  |
|  | MAPK8 |  |
|  | NXN |  |
|  | OLA1 |  |
|  | LOXL4 |  |
|  | ALG6 |  |
|  | YY1 |  |
|  | PRSS36 |  |
|  | RUNX1T1 |  |
|  | NDUFA5 |  |
|  | UBE2Z |  |
|  | MC2R |  |
|  | RAG1 |  |
|  | LMLN |  |
|  | CAPZA2 |  |
|  | MYSM1 |  |
|  | KITLG |  |
|  | N6AMT1 |  |
|  | PDE12 |  |
|  | PLOD2 |  |
|  | SREK1IP1 |  |
|  | AQP4 |  |
|  | TMEM41A |  |
|  | MYPN |  |
|  | NAA38 |  |
|  | ESCO1 |  |
|  | C1RL |  |
|  | ANTXR2 |  |
|  | SLC30A4 |  |
|  | AFF3 |  |
|  | FUT11 |  |
|  | DRAM2 |  |
|  | FMN1 |  |
|  | CSF2RB |  |
|  | PABPC1L2B |  |
|  | ITPKB |  |
|  | MPP2 |  |
|  | FAM47E |  |
|  | UGT8 |  |
|  | ATP8B2 |  |
|  | ID4 |  |
|  | TM7SF3 |  |
|  | STXBP6 |  |
|  | ZNF799 |  |
|  | MTX3 |  |
|  | ST6GALNAC5 |  |
|  | NHLRC2 |  |
|  | ZNF468 |  |
|  | NR4A3 |  |
|  | AGO3 |  |
|  | ANKH |  |
|  | BRIP1 |  |
|  | TYRP1 |  |
|  | PTBP2 |  |
|  | TTC5 |  |
|  | CRCP |  |
|  | HYOU1 |  |
|  | SLC26A9 |  |
|  | KCNN3 |  |
|  | CARF |  |
|  | IRF2BP2 |  |
|  | RSPH9 |  |
|  | NCOA3 |  |
|  | ACSL6 |  |
|  | ZFP91 |  |
|  | GPLD1 |  |
|  | FRK |  |
|  | MRE11A |  |
|  | FOXC1 |  |
|  | RBPJ |  |
|  | MFHAS1 |  |
|  | PABPC1L2A |  |
|  | SPEF2 |  |
|  | KRT77 |  |
|  | PPP1R16B |  |
|  | PAX7 |  |
|  | SLC3A1 |  |
|  | GPR110 |  |
|  | ITFG3 |  |
|  | TANC2 |  |
|  | ALPK2 |  |
|  | TENM1 |  |
|  | KIAA1161 |  |
|  | GLRA3 |  |
|  | AR |  |
|  | TREML1 |  |
|  | USH2A |  |
|  | MON1B |  |
|  | RSPO3 |  |
|  | SFMBT2 |  |
|  | TMEM132B |  |
|  | SLCO5A1 |  |
|  | PLCE1 |  |
|  | ZNF596 |  |
|  | KIAA0247 |  |
|  | ZNF652 |  |
|  | ADH5 |  |
|  | MTF2 |  |
|  | RAB39A |  |
|  | LOH12CR1 |  |
|  | SNAP23 |  |
|  | SUZ12 |  |
|  | NDUFAF6 |  |
|  | RPRD2 |  |
|  | AGO2 |  |
|  | CNTN3 |  |
|  | LRRC8C |  |
|  | APBB2 |  |
|  | ACLY |  |
|  | RANBP10 |  |
|  | RAB22A |  |
|  | NEBL |  |
|  | DDX19B |  |
|  | FCRL5 |  |
|  | PBX1 |  |
|  | GALNT6 |  |
|  | TANGO2 |  |
|  | GPRC5B |  |
|  | KCNC3 |  |
|  | ALDH3A2 |  |
|  | GPSM2 |  |
|  | SOCS6 |  |
|  | APH1B |  |
|  | SPPL2A |  |
|  | DNAJA4 |  |
|  | BRWD1 |  |
|  | SLC47A1 |  |
|  | KRBOX4 |  |
|  | BACH2 |  |
|  | PYGO1 |  |
|  | CTD-2140B24.4 |  |
|  | ABCB7 |  |
|  | TCF4 |  |
|  | C5orf63 |  |
|  | PRSS21 |  |
|  | CWF19L2 |  |
|  | KLK2 |  |
|  | CD200 |  |
|  | FARSB |  |
|  | PCDH18 |  |
|  | TCERG1 |  |
|  | STK17A |  |
|  | CACNA2D1 |  |
|  | SH3RF1 |  |
|  | GTDC1 |  |
|  | TAP2 |  |
|  | NOVA2 |  |
|  | RRN3 |  |
|  | MED21 |  |
|  | COL4A4 |  |
|  | DRD1 |  |
|  | CDC14B |  |
|  | ZDHHC7 |  |
|  | KCNE1 |  |
|  | AEBP2 |  |
|  | HMBOX1 |  |
|  | FRMD5 |  |
|  | FBXO45 |  |
|  | GTPBP10 |  |
|  | DDX52 |  |
|  | SMLR1 |  |
|  | FNDC3B |  |
|  | INTU |  |
|  | IFNLR1 |  |
|  | CACNA2D4 |  |
|  | DNM1L |  |
|  | HDAC11 |  |
|  | PSPC1 |  |
|  | CLCN4 |  |
|  | SH3BP2 |  |
|  | HNRNPD |  |
|  | YBX2 |  |
|  | IL17RE |  |
|  | CCNT2 |  |
|  | RAB2A |  |
|  | PVR |  |
|  | AKAP13 |  |
|  | PHEX |  |
|  | TPBG |  |
|  | IGF2BP1 |  |
|  | RAD51L3-RFFL |  |
|  | PON1 |  |
|  | PCNX |  |
|  | RSF1 |  |
|  | CTTNBP2NL |  |
|  | NIT2 |  |
|  | REEP5 |  |
|  | MMP16 |  |
|  | RALGPS1 |  |
|  | ACAP2 |  |
|  | PARVA |  |
|  | SOX12 |  |
|  | FBXO42 |  |
|  | PEX5L |  |
|  | C18orf21 |  |
|  | NEGR1 |  |
|  | NLE1 |  |
|  | SH3BGRL2 |  |
|  | ADCY5 |  |
|  | CDCA2 |  |
|  | TTC39A |  |
|  | ZFP30 |  |
|  | ADH6 |  |
|  | STON2 |  |
|  | CEP57 |  |
|  | KRBA2 |  |
|  | ZNF780A |  |
|  | KIAA1958 |  |
|  | PRPF4B |  |
|  | FRRS1 |  |
|  | TRPM3 |  |
|  | MCOLN3 |  |
|  | GAS7 |  |
|  | HOOK3 |  |
|  | SH3PXD2B |  |
|  | TGFBR2 |  |
|  | MEI4 |  |
|  | MCM9 |  |
|  | SUGT1 |  |
|  | IL12RB2 |  |
|  | LEPREL1 |  |
|  | RCSD1 |  |
|  | GAB2 |  |
|  | ZNF331 |  |
|  | ZFYVE1 |  |
|  | GIPC2 |  |
|  | SETD7 |  |
|  | EXTL3 |  |
|  | TRIM67 |  |
|  | MED28 |  |
|  | LMCD1 |  |
|  | MGA |  |
|  | FLRT2 |  |
|  | LCORL |  |
|  | PDHA1 |  |
|  | KIAA0319 |  |
|  | MAP2K6 |  |
|  | ZNF284 |  |
|  | UNC80 |  |
|  | YTHDF3 |  |
|  | METTL20 |  |
|  | MOCOS |  |
|  | EML5 |  |
|  | NKX6-2 |  |
|  | PTRF |  |
|  | SNX1 |  |
|  | DDX20 |  |
|  | KDM5B |  |
|  | CAMK2N1 |  |
|  | NUDCD3 |  |
|  | CECR2 |  |
|  | POTEI |  |
|  | RPS6KB1 |  |
|  | ITGA9 |  |
|  | SHC4 |  |
|  | ZNF687 |  |
|  | LSAMP |  |
|  | EXD2 |  |
|  | SEH1L |  |
|  | PRMT6 |  |
|  | SRSF12 |  |
|  | ZNHIT6 |  |
|  | LPGAT1 |  |
|  | KLHL14 |  |
|  | ZNF805 |  |
|  | USP32 |  |
|  | TBL1XR1 |  |
|  | MRTO4 |  |
|  | IFNE |  |
|  | GOLGA6A |  |
|  | SHROOM4 |  |
|  | PANX1 |  |
|  | FAM126B |  |
|  | NUDT16 |  |
|  | VPS35 |  |
|  | EPC1 |  |
|  | KIF5C |  |
|  | STRIP2 |  |
|  | POTEG |  |
|  | PDZD2 |  |
|  | RNF165 |  |
|  | ZNF783 |  |
|  | CYFIP2 |  |
|  | ABI3BP |  |
|  | WIZ |  |
|  | TRIM71 |  |
|  | GRIA1 |  |
|  | TRERF1 |  |
|  | SP1 |  |
|  | MICAL2 |  |
|  | PER1 |  |
|  | CACNA1E |  |
|  | DHRSX |  |
|  | ISLR2 |  |
|  | FLT1 |  |
|  | KIAA2018 |  |
|  | RPP40 |  |
|  | MYCL |  |
|  | ACPP |  |
|  | RAB37 |  |
|  | KAT6B |  |
|  | C11orf34 |  |
|  | C6orf222 |  |
|  | FRMPD3 |  |
|  | PPP1R12A |  |
|  | CFTR |  |
|  | KANK2 |  |
|  | NYNRIN |  |
|  | MYOCD |  |
|  | DLG2 |  |
|  | NFASC |  |
|  | CELF5 |  |
|  | GSX2 |  |
|  | RP11-766F14.2 |  |
|  | MYBL1 |  |
|  | RNF44 |  |
|  | CRY2 |  |
|  | CACNA2D2 |  |
|  | CNR2 |  |
|  | PRDM2 |  |
|  | PITPNM2 |  |
|  | OPN3 |  |
|  | SSTR3 |  |
|  | IER5L |  |
|  | PCSK2 |  |
|  | CLDN16 |  |
|  | ST18 |  |
|  | ZMYM3 |  |
|  | POTEM |  |
|  | ASH1L |  |
|  | BSN |  |
|  | NAIP |  |
|  | SLC11A1 |  |
|  | USP9Y |  |
|  | ASXL2 |  |
|  | NRF1 |  |
|  | ALS2 |  |
|  | FBXL7 |  |
|  | EDA |  |
|  | POLH |  |
|  | CLOCK |  |
|  | ADAR |  |
|  | FAT4 |  |
|  | CD93 |  |
|  | PCYT1B |  |
|  | SNX29 |  |
|  | SYNJ1 |  |
|  | SHISA7 |  |
|  | HIVEP3 |  |
|  | BACH1 |  |
|  | TTL |  |
|  | PREX2 |  |
|  | PCDH7 |  |
|  | CD28 |  |
|  | CDH1 |  |
|  | ISY1-RAB43 |  |
|  | SLC25A29 |  |
|  | GRIK3 |  |
|  | AURKA |  |
|  | PLCXD1 |  |
|  | TAF5L |  |
|  | GPR26 |  |
|  | HK2 |  |
|  | KSR2 |  |
|  | KIF21B |  |
|  | NKX3-1 |  |
|  | SYT2 |  |
|  | ZYG11B |  |
|  | ZNF831 |  |
|  | INO80D |  |
|  | DISC1 |  |
|  | SLC25A45 |  |
|  | CCDC144A |  |
|  | TPRXL |  |
|  | LGR5 |  |
|  | APOL4 |  |
|  | SFXN3 |  |
|  | NFIA |  |
|  | GABRA4 |  |
|  | ADPRH |  |
|  | ADAMTS2 |  |
|  | GPATCH11 |  |
|  | NT5C1A |  |
|  | HCN3 |  |
|  | KIAA1244 |  |
|  | MASP1 |  |
|  | RAPGEF1 |  |
|  | ACTN2 |  |
|  | SKA3 |  |
|  | POLR3H |  |
|  | KIAA0408 |  |
|  | SMARCE1 |  |
|  | SLC2A13 |  |
|  | HEG1 |  |
|  | BEND4 |  |
|  | ITGAV |  |
|  | GRID1 |  |
|  | KMT2A |  |
|  | GDF6 |  |
|  | RAB3D |  |
|  | NIN |  |
|  | EMC10 |  |
|  | MRAS |  |
|  | HIPK1 |  |
|  | MDGA1 |  |
|  | CEACAM1 |  |
|  | UBASH3B |  |
|  | FOXO1 |  |
|  | DIP2B |  |
|  | GPR126 |  |
|  | ITPRIPL2 |  |
|  | FBXO41 |  |
|  | KDM5A |  |
|  | DHX36 |  |
|  | CCDC103 |  |
|  | C16orf87 |  |
|  | TANC1 |  |
|  | MMP19 |  |
|  | COQ7 |  |
|  | ARHGEF7 |  |
|  | CPEB2 |  |
|  | NPTX1 |  |
|  | RFFL |  |
|  | ZNF518B |  |
|  | C5orf47 |  |
|  | ST8SIA1 |  |
|  | 44256 |  |
|  | ZNF460 |  |
|  | VPS33A |  |
|  | MITF |  |
|  | TMBIM4 |  |
|  | RNF141 |  |
|  | SCYL3 |  |
|  | DOCK11 |  |
|  | NRIP1 |  |
|  | LONRF2 |  |
|  | RPL15 |  |
|  | DSTYK |  |
|  | OSBPL8 |  |
|  | DCBLD2 |  |
|  | NANOS1 |  |
|  | STK35 |  |
|  | IL6ST |  |
|  | CTBP2 |  |
|  | SYNPO2 |  |
|  | TRPS1 |  |
|  | ETF1 |  |
|  | USP25 |  |
|  | PTCH1 |  |
|  | C1orf95 |  |
|  | RNF157 |  |
|  | HSPA13 |  |
|  | SORT1 |  |
|  | RBMXL1 |  |
|  | MAGI3 |  |
|  | ZNF326 |  |
|  | ABHD2 |  |
|  | DPYSL3 |  |
|  | PITPNC1 |  |
|  | DDX21 |  |
|  | JARID2 |  |
|  | PPME1 |  |
|  | KCND2 |  |
|  | PIP4K2A |  |
|  | REST |  |
|  | ITPR2 |  |
|  | APLF |  |
|  | MPRIP |  |
|  | GDF7 |  |
|  | FAM20A |  |
|  | ACOT13 |  |
|  | FKBP4 |  |
|  | P4HA2 |  |
|  | CLSTN2 |  |
|  | CLCN5 |  |
|  | FAM122A |  |
|  | NONO |  |
|  | CELF1 |  |
|  | MCC |  |
|  | KIAA0930 |  |
|  | VCAN |  |
|  | SLC7A11 |  |
|  | ZNF33B |  |
|  | DDR2 |  |
|  | EGFR |  |
|  | SH2B3 |  |
|  | PHACTR2 |  |
|  | HEBP2 |  |
|  | ZFHX3 |  |
|  | KCNQ3 |  |
|  | RAB5B |  |
|  | SMCHD1 |  |
|  | CHKA |  |
|  | SLC16A7 |  |
|  | RALGAPB |  |
|  | RP11-111M22.2 |  |
|  | DPY19L2 |  |
|  | WDHD1 |  |
|  | CNKSR2 |  |
|  | VSIG10 |  |
|  | VEGFC |  |
|  | MIPOL1 |  |
|  | EVI5 |  |
|  | ITFG1 |  |
|  | FBXW2 |  |
|  | FDX1 |  |
|  | SPATA17 |  |
|  | PTEN |  |
|  | CENPL |  |
|  | SRSF1 |  |
|  | GPRC5A |  |
|  | SLFN12 |  |
|  | WSCD1 |  |
|  | MAML2 |  |
|  | IER3IP1 |  |
|  | PPP1R1A |  |
|  | TNFRSF9 |  |
|  | AFF1 |  |
|  | TTC30A |  |
|  | GFI1 |  |
|  | CETN2 |  |
|  | PLEKHA8 |  |
|  | ATP2C1 |  |
|  | IGFBPL1 |  |
|  | GPCPD1 |  |
|  | C19orf12 |  |
|  | TAB3 |  |
|  | DOPEY1 |  |
|  | TRIQK |  |
|  | ZNF793 |  |
|  | DNAJC10 |  |
|  | TACC1 |  |
|  | ABCG8 |  |
|  | INSR |  |
|  | SELPLG |  |
|  | BZW1 |  |
|  | IGF1R |  |
|  | SOWAHB |  |
|  | SHANK2 |  |
|  | CCBL1 |  |
|  | HSD17B12 |  |
|  | RNF216 |  |
|  | ARFGEF2 |  |
|  | RAD50 |  |
|  | CNIH1 |  |
|  | ZCCHC17 |  |
|  | SSX2IP |  |
|  | DSEL |  |
|  | SPRYD7 |  |
|  | ABCB10 |  |
|  | GNB1 |  |
|  | ILDR2 |  |
|  | TLN1 |  |
|  | UBE2W |  |
|  | CDS2 |  |
|  | GGCX |  |
|  | SLC30A7 |  |
|  | AVPR1A |  |
|  | HS2ST1 |  |
|  | UACA |  |
|  | USP49 |  |
|  | ZMIZ2 |  |
|  | FRRS1L |  |
|  | LCTL |  |
|  | LYST |  |
|  | CDC20B |  |
|  | SLC4A8 |  |
|  | ENPEP |  |
|  | WDR72 |  |
|  | METAP1 |  |
|  | EXTL1 |  |
|  | ZBTB25 |  |
|  | PRMT10 |  |
|  | SLC30A1 |  |
|  | PLSCR1 |  |
|  | CCPG1 |  |
|  | CPSF6 |  |
|  | ST7L |  |
|  | PAPOLG |  |
|  | SSPN |  |
|  | TRPC4 |  |
|  | BAG4 |  |
|  | ATP11B |  |
|  | GNL3L |  |
|  | TBRG1 |  |
|  | ZBTB10 |  |
|  | LNPEP |  |
|  | STC2 |  |
|  | DCAF5 |  |
|  | LRRC28 |  |
|  | HECW1 |  |
|  | ABCA5 |  |
|  | ACVR1C |  |
|  | TRIP12 |  |
|  | SFT2D2 |  |
|  | ZNF560 |  |
|  | PRPF40A |  |
|  | ENAH |  |
|  | DNAL1 |  |
|  | NOX5 |  |
|  | ZNF585B |  |
|  | KATNBL1 |  |
|  | ZDHHC14 |  |
|  | GPR83 |  |
|  | BACE2 |  |
|  | ZSCAN16 |  |
|  | BTRC |  |
|  | ABI2 |  |
|  | KIAA0355 |  |
|  | ZNF24 |  |
|  | RPS6KA6 |  |
|  | SGPP2 |  |
|  | C1orf21 |  |
|  | LDLRAD4 |  |
|  | SETD9 |  |
|  | HOXD4 |  |
|  | GTF2A1 |  |
|  | ZNF490 |  |
|  | AP5B1 |  |
|  | FIGN |  |
|  | SOX5 |  |
|  | PHF14 |  |
|  | SLC5A12 |  |
|  | CCDC127 |  |
|  | PAG1 |  |
|  | NUP98 |  |
|  | MLLT6 |  |
|  | WDR53 |  |
|  | PLCL1 |  |
|  | ZNF286B |  |
|  | ATXN7L1 |  |
|  | G3BP1 |  |
|  | SMAD2 |  |
|  | ZNF704 |  |
|  | GAN |  |
|  | HEMK1 |  |
|  | VN1R1 |  |
|  | AP4S1 |  |
|  | CSRNP1 |  |
|  | ZEB2 |  |
|  | SNRPE |  |
|  | CCDC171 |  |
|  | ING2 |  |
|  | ZNF230 |  |
|  | PPP3CC |  |
|  | XRCC2 |  |
|  | SNX2 |  |
|  | MEF2C |  |
|  | PTAR1 |  |
|  | CCDC160 |  |
|  | SPIRE1 |  |
|  | IRAK1BP1 |  |
|  | SBK1 |  |
|  | SYT14 |  |
|  | SMARCC1 |  |
|  | METTL2B |  |
|  | TMC7 |  |
|  | PPIL6 |  |
|  | RNF2 |  |
|  | EIF5AL1 |  |
|  | RBMS3 |  |
|  | SLC33A1 |  |
|  | MSANTD2 |  |
|  | H2AFJ |  |
|  | PARVB |  |
|  | QKI |  |
|  | TSLP |  |
|  | ZNF626 |  |
|  | MAP4 |  |
|  | ZNF862 |  |
|  | DCLK3 |  |
|  | ZNF286A |  |
|  | HUNK |  |
|  | PEG3 |  |
|  | TSPEAR |  |
|  | GPR56 |  |
|  | TGFA |  |
|  | PRICKLE2 |  |
|  | ATG9B |  |
|  | PTK7 |  |
|  | ZBTB3 |  |
|  | PTPRT |  |
|  | CNOT6L |  |
|  | ZNF347 |  |
|  | SPECC1L |  |
|  | ZNF324B |  |
|  | IQGAP3 |  |
|  | ABCC12 |  |
|  | AFAP1L1 |  |
|  | ELMSAN1 |  |
|  | SRRM4 |  |
|  | FOXN1 |  |
|  | LDOC1L |  |
|  | MSL1 |  |
|  | PCLO |  |
|  | AIM1 |  |
|  | PLXNA2 |  |
|  | NLRC3 |  |
|  | SESN3 |  |
|  | AMER1 |  |
|  | TRIM10 |  |
|  | PFKFB4 |  |
|  | WDR52 |  |
|  | NRXN1 |  |
|  | HCG27 |  |
|  | SIGLEC1 |  |
|  | PEAK1 |  |
|  | SH3BP4 |  |
|  | ZNF710 |  |
|  | SLC6A5 |  |
|  | WDFY3 |  |
|  | PIK3R5 |  |
|  | TXNRD2 |  |
|  | CXorf36 |  |
|  | KIRREL |  |
|  | VPS37A |  |
|  | IRF1 |  |
|  | MLEC |  |
|  | HDHD1 |  |
|  | C14orf166 |  |
|  | EMP2 |  |
|  | ZC3HAV1 |  |
|  | WDFY1 |  |
|  | MAP3K2 |  |
|  | TYW5 |  |
|  | NDFIP2 |  |
|  | MIER3 |  |
|  | FAM53C |  |
|  | NEDD4 |  |
|  | AQP7 |  |
|  | RXRA |  |
|  | ENDOD1 |  |
|  | STX6 |  |
|  | RFK |  |
|  | SHROOM3 |  |
|  | PTPRB |  |
|  | IFNAR1 |  |
|  | NAP1L1 |  |
|  | TPM3 |  |
|  | FMNL3 |  |
|  | RAP1A |  |
|  | STEAP4 |  |
|  | UBR1 |  |
|  | RASSF9 |  |
|  | LAT |  |
|  | PGAM1 |  |
|  | SH2D4A |  |
|  | RNF169 |  |
|  | PRKAR2B |  |
|  | HNRNPR |  |
|  | CDK9 |  |
|  | IGFBP5 |  |
|  | BCORL1 |  |
|  | TMEM241 |  |
|  | REV1 |  |
|  | RPS6KA5 |  |
|  | TRIM24 |  |
|  | NABP1 |  |
|  | MICA |  |
|  | TULP4 |  |
|  | TERF2 |  |
|  | CHRM3 |  |
|  | FAM120B |  |
|  | FBXL20 |  |
|  | SOS1 |  |
|  | PDE7A |  |
|  | NR3C1 |  |
|  | ADAT1 |  |
|  | CDKN1B |  |
|  | VWDE |  |
|  | ZNF770 |  |
|  | GINS2 |  |
|  | COX10 |  |
|  | CCDC93 |  |
|  | PTBP3 |  |
|  | FBXO32 |  |
|  | MAP3K9 |  |
|  | ZNF519 |  |
|  | GGA2 |  |
|  | PLA2R1 |  |
|  | JAKMIP2 |  |
|  | BMS1 |  |
|  | NR1H4 |  |
|  | LARP4B |  |
|  | GFRA1 |  |
|  | HPRT1 |  |
|  | HOXD12 |  |
|  | KLHL18 |  |
|  | ERCC6 |  |
|  | GSTO1 |  |
|  | PRPSAP1 |  |
|  | RAB10 |  |
|  | RAB3C |  |
|  | FUBP1 |  |
|  | PTCHD4 |  |
|  | HNRNPLL |  |
|  | PHF3 |  |
|  | APOOL |  |
|  | ZMYM2 |  |
|  | LRP8 |  |
|  | POLR1C |  |
|  | DTNA |  |
|  | CENPC |  |
|  | AVL9 |  |
|  | MCM3AP |  |
|  | WBP11 |  |
|  | ACP1 |  |
|  | PDE7B |  |
|  | ZNF695 |  |
|  | MICALCL |  |
|  | SHH |  |
|  | SLC27A4 |  |
|  | SOGA3 |  |
|  | HTATIP2 |  |
|  | EMC2 |  |
|  | PPM1L |  |
|  | CNOT6 |  |
|  | OGT |  |
|  | PTPN9 |  |
|  | RP11-664D7.4 |  |
|  | RNASET2 |  |
|  | DIEXF |  |
|  | ERMP1 |  |
|  | CCDC138 |  |
|  | RALY |  |
|  | NCOR1 |  |
|  | SMG1 |  |
|  | NOL10 |  |
|  | ERCC8 |  |
|  | BUB1 |  |
|  | SKP1 |  |
|  | MLLT3 |  |
|  | RAB13 |  |
|  | PAX6 |  |
|  | TDG |  |
|  | AGPS |  |
|  | ARRB1 |  |
|  | TTF2 |  |
|  | CSF3R |  |
|  | DDHD1 |  |
|  | BMPR2 |  |
|  | CLUAP1 |  |
|  | EDC3 |  |
|  | ZNF566 |  |
|  | KIAA0368 |  |
|  | RAPH1 |  |
|  | PGR |  |
|  | CCDC109B |  |
|  | SH3KBP1 |  |
|  | SART3 |  |
|  | DPY19L1 |  |
|  | SLC24A1 |  |
|  | NEK3 |  |
|  | SEC62 |  |
|  | ATM |  |
|  | IQSEC1 |  |
|  | RNF217 |  |
|  | GSTO2 |  |
|  | GID4 |  |
|  | UBE2B |  |
|  | C15orf38 |  |
|  | PIK3C3 |  |
|  | VPS13D |  |
|  | CENPP |  |
|  | TWISTNB |  |
|  | RWDD1 |  |
|  | RPS29 |  |
|  | GOSR1 |  |
|  | ATRX |  |
|  | STT3A |  |
|  | RGP1 |  |
|  | FAM69B |  |
|  | SLC9A7 |  |
|  | CSRNP2 |  |
|  | TSPAN31 |  |
|  | HNRNPU |  |
|  | WDR12 |  |
|  | TPST2 |  |
|  | THUMPD3 |  |
|  | ARSE |  |
|  | C6 |  |
|  | PCDHB16 |  |
|  | CRNKL1 |  |
|  | UQCRB |  |
|  | SUPV3L1 |  |
|  | NUFIP2 |  |
|  | RSRC2 |  |
|  | C7orf55-LUC7L2 |  |
|  | TTC28 |  |
|  | CENPF |  |
|  | TTLL4 |  |
|  | TRNAU1AP |  |
|  | AJUBA |  |
|  | BPTF |  |
|  | DYRK2 |  |
|  | CNOT2 |  |
|  | KIAA0141 |  |
|  | TUSC3 |  |
|  | ANKRD32 |  |
|  | CCSER2 |  |
|  | RPL24 |  |
|  | TMEM127 |  |
|  | SGOL1 |  |
|  | EIF5 |  |
|  | SRSF7 |  |
|  | SLCO1B3 |  |
|  | SLC22A3 |  |
|  | TSPYL1 |  |
|  | AQR |  |
|  | VAMP4 |  |
|  | WEE1 |  |
|  | PNO1 |  |
|  | LUC7L2 |  |
|  | IRF6 |  |
|  | STARD5 |  |
|  | DMRTA1 |  |
|  | TRMT5 |  |
|  | WDR92 |  |
|  | PCDHB5 |  |
|  | BID |  |
|  | HIF1AN |  |
|  | CDKN2C |  |
|  | FAM114A2 |  |
|  | OPTN |  |
|  | BEND3 |  |
|  | RILPL1 |  |
|  | C14orf37 |  |
|  | PNN |  |
|  | SMAD4 |  |
|  | TBC1D19 |  |
|  | LRP12 |  |
|  | LGALS8 |  |
|  | BDNF |  |
|  | HNRNPA3 |  |
|  | NGFRAP1 |  |
|  | ICMT |  |
|  | RAB3B |  |
|  | ZNF426 |  |
|  | KIAA1524 |  |
|  | RNF19B |  |
|  | TFDP2 |  |
|  | IKZF3 |  |
|  | ANGEL2 |  |
|  | CNNM2 |  |
|  | CCDC50 |  |
|  | ZNF404 |  |
|  | TPD52 |  |
|  | PIGH |  |
|  | KCTD16 |  |
|  | MNAT1 |  |
|  | FAM208A |  |
|  | MRPS14 |  |
|  | MRPL30 |  |
|  | ENPP5 |  |
|  | C2orf15 |  |
|  | ARHGAP31 |  |
|  | FAM229B |  |
|  | EGLN1 |  |
|  | LPHN3 |  |
|  | ATP6V1B2 |  |
|  | MRRF |  |
|  | SLC35C2 |  |
|  | FOXP4 |  |
|  | POU6F1 |  |
|  | CHSY3 |  |
|  | CDCA3 |  |
|  | RPAP2 |  |
|  | CTH |  |
|  | SUSD1 |  |
|  | RNF213 |  |
|  | HMGN3 |  |
|  | ADRBK2 |  |
|  | MXRA7 |  |
|  | GIN1 |  |
|  | HOXB13 |  |
|  | HAT1 |  |
|  | NUP205 |  |
|  | WDR3 |  |
|  | OCIAD2 |  |
|  | FAM46C |  |
|  | CASP9 |  |
|  | RBM18 |  |
|  | SPDL1 |  |
|  | SATB1 |  |
|  | B3GALT1 |  |
|  | CHML |  |
|  | TLN2 |  |
|  | RRAGD |  |
|  | TARDBP |  |
|  | DIMT1 |  |
|  | OIP5 |  |
|  | NEO1 |  |
|  | TOR1A |  |
|  | ZNF250 |  |
|  | TMED10 |  |
|  | ATG14 |  |
|  | POLI |  |
|  | KIN |  |
|  | EIF1AD |  |
|  | HSP90B1 |  |
|  | LSM6 |  |
|  | ZNF207 |  |
|  | ELAVL1 |  |
|  | TNIK |  |
|  | TAX1BP1 |  |
|  | FAM179A |  |
|  | PHF20L1 |  |
|  | PGK1 |  |
|  | SEMA6A |  |
|  | IVNS1ABP |  |
|  | C19orf66 |  |
|  | CCDC115 |  |
|  | TGIF1 |  |
|  | PPIP5K2 |  |
|  | ALG14 |  |
|  | C4orf32 |  |
|  | DNAJC30 |  |
|  | TMEM218 |  |
|  | ETV6 |  |
|  | ZNF280C |  |
|  | ETV1 |  |
|  | ADAT2 |  |
|  | DKK3 |  |
|  | HDLBP |  |
|  | SNX16 |  |
|  | ANXA11 |  |
|  | YBX1 |  |
|  | MRPL45 |  |
|  | PTGS1 |  |
|  | FAM204A |  |
|  | SRSF6 |  |
|  | TUBB |  |
|  | TRPM7 |  |
|  | CDR1as |  |
|  | CDH7 |  |
|  | NSUN4 |  |
|  | TFCP2 |  |
|  | CD84 |  |
|  | SOCS7 |  |
|  | SLC44A1 |  |
|  | SMYD2 |  |
|  | OSER1 |  |
|  | APTX |  |
|  | AGTPBP1 |  |
|  | TPGS2 |  |
|  | CAMLG |  |
|  | KIAA1551 |  |
|  | RNF7 |  |
|  | FAM47E-STBD1 |  |
|  | CSTF2T |  |
|  | TMED4 |  |
|  | CITED2 |  |
|  | CRTAP |  |
|  | MED9 |  |
|  | CDKN3 |  |
|  | SULF2 |  |
|  | GDAP2 |  |
|  | DLD |  |
|  | FGFR1OP |  |
|  | ZBED3 |  |
|  | RNF214 |  |
|  | PLA2G16 |  |
|  | MTL5 |  |
|  | DNTTIP2 |  |
|  | GALK2 |  |
|  | ALG13 |  |
|  | DCPS |  |
|  | UQCR10 |  |
|  | VPS53 |  |
|  | ZFYVE20 |  |
|  | DCAF10 |  |
|  | ZNF223 |  |
|  | CDK13 |  |
|  | ABCF1 |  |
|  | PSD4 |  |
|  | CNKSR3 |  |
|  | MRPL44 |  |
|  | IPO9 |  |
|  | NSL1 |  |
|  | FAM105A |  |
|  | ZNF860 |  |
|  | IWS1 |  |
|  | PTCHD1 |  |
|  | CLN8 |  |
|  | TXNL1 |  |
|  | CHAF1B |  |
|  | IGSF3 |  |
|  | COBLL1 |  |
|  | COL9A2 |  |
|  | YLPM1 |  |
|  | LIG3 |  |
|  | RCAN1 |  |
|  | BTBD1 |  |
|  | YAF2 |  |
|  | NUF2 |  |
|  | ZNF354B |  |
|  | DIP2A |  |
|  | ZNF180 |  |
|  | SLC35D2 |  |
|  | NUDT9 |  |
|  | HLA-A |  |
|  | SMURF2 |  |
|  | RRP8 |  |
|  | C1orf112 |  |
|  | TOMM6 |  |
|  | MRPL19 |  |
|  | HERPUD1 |  |
|  | TRIP4 |  |
|  | DNAJC21 |  |
|  | SPAG16 |  |
|  | SHB |  |
|  | PTCD3 |  |
|  | SOD2 |  |
|  | STX3 |  |
|  | NXPE3 |  |
|  | USO1 |  |
|  | CENPM |  |
|  | CEP128 |  |
|  | FAM135A |  |
|  | GPR37 |  |
|  | PNISR |  |
|  | KPNA3 |  |
|  | KCNK1 |  |
|  | RBP4 |  |
|  | PACS2 |  |
|  | AMHR2 |  |
|  | ZBTB20 |  |
|  | VPS18 |  |
|  | COX15 |  |
|  | MRPL17 |  |
|  | ABCG2 |  |
|  | LYN |  |
|  | MTO1 |  |
|  | ZWINT |  |
|  | DLGAP5 |  |
|  | IMPA1 |  |
|  | ARHGAP29 |  |
|  | ERBB2IP |  |
|  | DYRK3 |  |
|  | MSANTD4 |  |
|  | GMPR |  |
|  | PAPOLA |  |
|  | RAB6B |  |
|  | COG6 |  |
|  | DNAJC18 |  |
|  | RNF115 |  |
|  | GRSF1 |  |
|  | GABRB1 |  |
|  | UBA6 |  |
|  | CHST12 |  |
|  | DDI2 |  |
|  | SIPA1 |  |
|  | RBMS2 |  |
|  | MED16 |  |
|  | NCKAP1 |  |
|  | MAPRE2 |  |
|  | KIAA0020 |  |
|  | SERINC1 |  |
|  | ADAP1 |  |
|  | LPXN |  |
|  | WDR37 |  |
|  | LITAF |  |
|  | PTGDR |  |
|  | MRPL46 |  |
|  | MCTS1 |  |
|  | RP11-122A3.2 |  |
|  | EIF2A |  |
|  | EGF |  |
|  | DLK1 |  |
|  | ADI1 |  |
|  | UBD |  |
|  | ANKHD1 |  |
|  | UROS |  |
|  | CPD |  |
|  | ARAP1 |  |
|  | TSPAN14 |  |
|  | TACO1 |  |
|  | GLUL |  |
|  | ZNF90 |  |
|  | GRK5 |  |
|  | ZNF670 |  |
|  | MSRB2 |  |
|  | HDAC2 |  |
|  | PPP5C |  |
|  | CRISP1 |  |
|  | HSPB3 |  |
|  | YTHDC1 |  |
|  | URGCP |  |
|  | HNRNPA0 |  |
|  | DOCK7 |  |
|  | GSKIP |  |
|  | TIGD2 |  |
|  | PIGP |  |
|  | IBSP |  |
|  | SLC25A4 |  |
|  | MORC1 |  |
|  | TM2D2 |  |
|  | G6PC2 |  |
|  | PHKG2 |  |
|  | DDX53 |  |
|  | RAB18 |  |
|  | EXOC3 |  |
|  | CCNG1 |  |
|  | RAB40B |  |
|  | SPECC1 |  |
|  | ORM1 |  |
|  | USP9X |  |
|  | RWDD2B |  |
|  | RPS23 |  |
|  | YIPF4 |  |
|  | CYB5R4 |  |
|  | LRRC2 |  |
|  | BAG1 |  |
|  | IMP4 |  |
|  | CCR4 |  |
|  | RASA1 |  |
|  | OR7A5 |  |
|  | FBN1 |  |
|  | UPK1A |  |
|  | TMEM86B |  |
|  | EMC1 |  |
|  | MSH2 |  |
|  | CRIPT |  |
|  | RP13-996F3.4 |  |
|  | COMTD1 |  |
|  | PPWD1 |  |
|  | AKR7A2 |  |
|  | UBP1 |  |
|  | CORO1C |  |
|  | C14orf23 |  |
|  | F5 |  |
|  | TMEM179 |  |
|  | WDR7 |  |
|  | TMEM14B |  |
|  | MRPL3 |  |
|  | TRDN |  |
|  | IFT74 |  |
|  | POLR3A |  |
|  | DNLZ |  |
|  | WDR77 |  |
|  | HAS2 |  |
|  | MRPS23 |  |
|  | C1orf50 |  |
|  | LMBR1 |  |
|  | NSUN3 |  |
|  | GOLGA6B |  |
|  | PRH2 |  |
|  | CTSS |  |
|  | AHSA2 |  |
|  | GPR22 |  |
|  | SPATS2L |  |
|  | NKAPL |  |
|  | DYNLL1 |  |
|  | TRIT1 |  |
|  | CADM1 |  |
|  | C6orf25 |  |
|  | DYRK1A |  |
|  | TXK |  |
|  | ZBED4 |  |
|  | RAI14 |  |
|  | EHF |  |
|  | C3 |  |
|  | RNF20 |  |
|  | GRPR |  |
|  | BRIX1 |  |
|  | VNN3 |  |
|  | CLCC1 |  |
|  | S1PR1 |  |
|  | IL17RA |  |
|  | DNASE1L3 |  |
|  | VGLL2 |  |
|  | RABIF |  |
|  | NFAM1 |  |
|  | ZNF283 |  |
|  | FAM162B |  |
|  | MCHR2 |  |
|  | DHX30 |  |
|  | ACP6 |  |
|  | CCDC59 |  |
|  | PCDH11X |  |
|  | WDR33 |  |
|  | ZNF611 |  |
|  | CUL3 |  |
|  | SLC25A12 |  |
|  | DMGDH |  |
|  | KCNH1 |  |
|  | CCNB1 |  |
|  | BST1 |  |
|  | PKHD1L1 |  |
|  | SLC35E2 |  |
|  | GEMIN8 |  |
|  | CYP2A7 |  |
|  | CMC1 |  |
|  | XRCC5 |  |
|  | PEX7 |  |
|  | CAMSAP1 |  |
|  | MGAT4C |  |
|  | UQCR11 |  |
|  | TPCN2 |  |
|  | GRIN2A |  |
|  | SIGLEC6 |  |
|  | FAR2 |  |
|  | CNTNAP5 |  |
|  | PYCARD |  |
|  | SP140L |  |
|  | ITGA1 |  |
|  | UBC |  |
|  | PQLC1 |  |
|  | TPM2 |  |
|  | CLEC17A |  |
|  | ORC6 |  |
|  | GRAP2 |  |
|  | RPL7L1 |  |
|  | HMGCR |  |
|  | MELK |  |
|  | PDHB |  |
|  | CRP |  |
|  | FBXL3 |  |
|  | G6PC |  |
|  | TCF23 |  |
|  | TOR1AIP1 |  |
|  | THBS1 |  |
|  | ACKR2 |  |
|  | MAL |  |
|  | NRXN3 |  |
|  | CSMD1 |  |
|  | CDH19 |  |
|  | KCNC2 |  |
|  | AVPI1 |  |
|  | TCEAL4 |  |
|  | BMP10 |  |
|  | MSC |  |
|  | MOBP |  |
|  | NNT |  |
|  | POPDC2 |  |
|  | IL1RAPL1 |  |
|  | STARD3NL |  |
|  | RBM22 |  |
|  | MAPK10 |  |
|  | TMEM252 |  |
|  | COL23A1 |  |
|  | ZNF763 |  |
|  | TNFSF14 |  |
|  | DCUN1D5 |  |
|  | SIGLEC5 |  |
|  | RCN2 |  |
|  | APOB |  |
|  | SYNRG |  |
|  | KCNK5 |  |
|  | EHD3 |  |
|  | MXRA8 |  |
|  | TLR4 |  |
|  | GATSL2 |  |
|  | SC5D |  |
|  | HMGCS1 |  |
|  | WHSC1 |  |
|  | LRRC40 |  |
|  | GUCY1A3 |  |
|  | NBPF10 |  |
|  | HSD11B1 |  |
|  | MRPS21 |  |
|  | GOLM1 |  |
|  | DENND2A |  |
|  | METTL2A |  |
|  | UCP3 |  |
|  | GPR183 |  |
|  | RPL37A |  |
|  | BLVRA |  |
|  | CELF3 |  |
|  | FZD8 |  |
|  | CALR |  |
|  | DSE |  |
|  | EBP |  |
|  | SSTR2 |  |
|  | PRMT7 |  |
|  | CRHR2 |  |
|  | IFIT1 |  |
|  | ZFAND5 |  |
|  | AKAP6 |  |
|  | MED29 |  |
|  | MCTP2 |  |
|  | FGG |  |
|  | TLR8 |  |
|  | SLFN12L |  |
|  | GGACT |  |
|  | SYT9 |  |
|  | TMEM106C |  |
|  | PSMB9 |  |
|  | UGT2B4 |  |
|  | SLC2A9 |  |
|  | GPR125 |  |
|  | KLHL31 |  |
|  | TIMM50 |  |
|  | UBE2D3 |  |
|  | ERCC1 |  |
|  | HIST1H2BG |  |
|  | GRM4 |  |
|  | AKR1C2 |  |
|  | HSPA14 |  |
|  | C8A |  |
|  | AKR1D1 |  |
|  | ERP44 |  |
|  | CPE |  |
|  | TRAF3IP1 |  |
|  | PCDHB15 |  |
|  | ZNF366 |  |
|  | TTR |  |
|  | HOPX |  |
|  | ZNF436 |  |
|  | HPGD |  |
|  | FOSL2 |  |
|  | NDNF |  |
|  | PDLIM3 |  |
|  | MFSD2A |  |
|  | RAB43 |  |
|  | RASAL2 |  |
|  | METTL24 |  |
|  | MRPL39 |  |
|  | FCRL1 |  |
|  | ZNF8 |  |
|  | SHMT1 |  |
|  | LAMA4 |  |
|  | FAM216B |  |
|  | SH3TC2 |  |
|  | GDE1 |  |
|  | MFSD6 |  |
|  | ITGB1 |  |
|  | ITGB1BP1 |  |
|  | TBC1D9 |  |
|  | C5orf55 |  |
|  | FAM115A |  |
|  | CIRH1A |  |
|  | SHC3 |  |
|  | SOX17 |  |
|  | MYO1F |  |
|  | TG |  |
|  | POU2F2 |  |
|  | COX7B |  |
|  | PSTPIP1 |  |
|  | ANKFY1 |  |
|  | LIPG |  |
|  | MAT1A |  |
|  | CFL2 |  |
|  | IVD |  |
|  | ZBTB14 |  |
|  | KIAA1522 |  |
|  | MXD4 |  |
|  | KAT6A |  |
|  | RALBP1 |  |
|  | VNN1 |  |
|  | SORCS3 |  |
|  | NUDT10 |  |
|  | ZNF132 |  |
|  | TOR2A |  |
|  | NIPSNAP3B |  |
