# Supplementary material for: CircMTO1 suppresses hepatocellular carcinoma progression via the miR-541-5p/ZIC1 axis by regulating Wnt/β-catenin signaling pathway and epithelial-to-mesenchymal transition
Source: Cell Death Dis. 2021 Dec 20;13(1):12. doi: 10.1038/s41419-021-04464-3 (PMC8688446; doi:10.1038/s41419-021-04464-3)
Supplement: Supplementary file 5 — Table S4 [file 41419_2021_4464_MOESM5_ESM.docx]

Table S4 The 239 genes possible targeted by miR-541-5p predicted by all miRDB, TargetScan and ENCORI/starbase databases.

| Target genes | | | | | |
| --- | --- | --- | --- | --- | --- |
| ZNF208 | GPX8 | CLIC4 | PAFAH1B2 | SLC7A6 | GLI3 |
| ZNF676 | TMX4 | FAM172A | DERL1 | HIP1 | C3orf14 |
| RBBP4 | ACER3 | CHD8 | TMOD2 | AFF4 | ATP2B1 |
| ASTE1 | DAB2 | FRMD3 | MRPL42 | CTNND1 | PRKD3 |
| C12orf75 | KLHL12 | VPS13C | SEC24A | CAMKK2 | CHCHD7 |
| PANK2 | PCDHB11 | MBNL3 | TMEM181 | GATAD2A | VAPA |
| NRM | CREG1 | RANBP9 | NARS2 | WWC2 | TENM3 |
| DENND1B | DHX33 | CTDP1 | CRKL | JAZF1 | ERC1 |
| TINF2 | SLC46A3 | MEX3A | MDM2 | FMR1 | WDR41 |
| PPHLN1 | KIAA1191 | ENC1 | SMARCD2 | PITPNB | SESTD1 |
| GABARAPL2 | PCDHB3 | ING3 | HNRNPF | TMEM106B | ATXN1 |
| RMI1 | PI15 | SYNCRIP | FNIP2 | HELZ | RPS6KA3 |
| SYAP1 | DHX40 | PIGM | ZNF619 | SLC1A2 | NFIB |
| RNPEP | MAP4K5 | CHM | VPS4A | EXPH5 | PRSS36 |
| CDC42EP4 | ANKRD46 | MAPK1 | ZNF629 | ANKRD13C | MYSM1 |
| GFPT1 | C18orf32 | DENND3 | MED14 | BMPR1B | ANTXR2 |
| PICALM | CAPRIN2 | FGD4 | SLFN11 | SMC2 | SPEF2 |
| TMED7 | RUFY2 | AMFR | MYO5A | MED13 | GTPBP10 |
| PTGR2 | ULK2 | PDP2 | LPP | RAPGEF2 | SH3BP2 |
| LUM | GNA12 | SLC25A53 | SPTLC2 | ONECUT2 | HNRNPD |
| SH3YL1 | UST | FAM114A1 | ARNTL | PAPPA | CTTNBP2NL |
| RBM17 | CMC2 | NEK7 | KTN1 | QSER1 | HOOK3 |
| ZMYND19 | RHNO1 | BLCAP | SOCS4 | ARID4B | KDM5B |
| COPS2 | PHF6 | ARL4C | ZCCHC14 | PPP2R5E | TBL1XR1 |
| METTL15 | TMEM248 | PRRC2C | MACC1 | CDC42BPA | EPC1 |
| LCLAT1 | PAH | ZIC1 | ADD1 | ANAPC16 | PRDM2 |
| TMEM161B | NRAS | CHD6 | AHCYL1 | ZNF138 | RPL15 |
| EIF5A2 | CTDSPL2 | AP3S2 | PUM2 | SDC2 | VCAN |
| ESPL1 | GRB2 | ZSCAN25 | GSPT1 | ZFP1 | WDR72 |
| FBXO33 | SGTB | FNIP1 | ZNF624 | STAG1 | CPSF6 |
| KCTD12 | CPNE8 | DHX38 | RPP14 | CDK6 | ATP11B |
| PRPF38B | USP15 | ZKSCAN1 | SMAP1 | PDGFRA | NAP1L1 |
| GRPEL2 | RPL17-C18orf32 | BDP1 | ARFIP1 | SOAT1 | PGAM1 |
| ZNF189 | GNPNAT1 | NUMB | GNA13 | ZEB1 | ZMYM2 |
| SPRED1 | ELOVL6 | MEF2A | LPCAT1 | UBE2J1 | RAPH1 |
| UEVLD | SUMO1 | USP38 | ZNF618 | PHIP | NUFIP2 |
| ZNF586 | RNF144A | FRS2 | DZIP1 | CSE1L | PNN |
| PAQR3 | PLCXD3 | HCCS | SLC16A4 | RTKN2 | ZNF426 |
| RSBN1L | CSTB | LIMD1 | SLITRK4 | VPS13B | ELAVL1 |
| ZNF708 | KPNA1 | NCKAP5 | WNK3 | SLC40A1 |  |
