# Supplementary material for: CircMTO1 suppresses hepatocellular carcinoma progression via the miR-541-5p/ZIC1 axis by regulating Wnt/β-catenin signaling pathway and epithelial-to-mesenchymal transition
Source: Cell Death Dis. 2021 Dec 20;13(1):12. doi: 10.1038/s41419-021-04464-3 (PMC8688446; doi:10.1038/s41419-021-04464-3)
Supplement: Supplementary file 6 — Table S5 [file 41419_2021_4464_MOESM6_ESM.docx]

Table S5 Primers sequence

| **Name** | **Primer Sequence (5’-3’)** |
| --- | --- |
| circMTO1-F | GAGCTGTAGAAGATCTTATTC |
| circMTO1-R | CACAGGCCATCCAAGGCATC |
| MTO1-F | TGCATCAGAGGCTTGGAGAA |
| MTO1-R | AAGGAAGGGGTGATCTGACG |
| miR-541-5p-F | CGAAAGGATTCTGCTGTCGGT |
| miR-541-5p-R | AGTGCAGGGTCCGAGGTATT |
| miR-541-5p-P | GTCGTATCCAGTGCAGGGTCCGAGGTATTCGCACTGGATACGACAGTGGG |
| miR-9-5p-F | GCGCGTCTTTGGTTATCTAGCT |
| miR-9-5p-R | AGTGCAGGGTCCGAGGTATT |
| miR-9-5p-P | GAGGTATTCGCACTGGATACGACTCATAC |
| ZIC1-F | GCGTCCTTTTGTGGATCTTTAA |
| ZIC1-R | AGTAATCACATCTGCTTCTGGG |
| c-myc -F | GTCAAGAGGCGAACACACAAC |
| c-myc -R | TTGGACGGACAGGATGTATGC |
| cyclin D1 -F | GCTGCGAAGTGGAAACCATC |
| cyclin D1 -R | CCTCCTTCTGCACACATTTGAA |
| E-cadherin -F | GTCCTGGGCAGACTGAATTT |
| E-cadherin -R | GACCAAGAAATGGATCTGTGG |
| N-cadherin -F | TGGACCATCACTCGGCTTA |
| N-cadherin -R | ACACTGGCAAACCTTCACG |
| Vimentin-F | CGAGGAGAGCAGGATTTCTC |
| Vimentin-R | GGTATCAACCAGAGGGAGTGA |
| β-catenin -F | AAAGCGGCTGTTAGTCACTGG |
| β-catenin -R | CGAGTCATTGCATACTGTCCAT |
| MMP2-F | GATACCCCTTTGACGGTAAGGA |
| MMP2 -R | CCTTCTCCCAAGGTCCATAGC |
| GAPDH-F | GCACCGTCAAGGCTGAGAAC |
| GAPDH-R | GCCTTCTCCATGGTGGTGAA |
| U6-F | CTCGCTTCGGCAGCACATATACTA |
| U6-R | ACGAATTTGCGTGTCATCCTTGCG |
