# Supplementary material for: CircMTO1 suppresses hepatocellular carcinoma progression via the miR-541-5p/ZIC1 axis by regulating Wnt/β-catenin signaling pathway and epithelial-to-mesenchymal transition
Source: Cell Death Dis. 2021 Dec 20;13(1):12. doi: 10.1038/s41419-021-04464-3 (PMC8688446; doi:10.1038/s41419-021-04464-3)
Supplement: Supplementary file 7 — Table S6 [file 41419_2021_4464_MOESM7_ESM.docx]

Table S6 The information of the siRNAs, miRNA mimics, and inhibitor.

| **Name** | **Sequence (5’-3’)** |
| --- | --- |
| circMTO1 siRNA-1 | sense: UCUGACCCAAAACAACCCCTT |
|  | antisense: GGGGUUGUUUUGGGUCAGATT |
| circMTO1 siRNA-2 | sense: UGACCCAAAACAACCCCACTT |
|  | antisense: GUGGGGUUGUUUUGGGUCATT |
| circMTO1 siRNA-3 | sense: AUCUGACCCAAAACAACCCTT |
|  | antisense: GGGUUGUUUUGGGUCAGAUTT |
| ZIC1 siRNA-1 | sense: CGUAUGUACACUUUAGUUUCCAGAA |
|  | antisense: UUCUGGAAACUAAAGUGUACAUACGAG |
| ZIC1 siRNA-2 | sense: GCCUGGGCAUCAACCCGUUTT |
|  | antisense: AACGGGUUGAUGCCCAGGCTT |
| ZIC1 siRNA-3 | sense: GGGACUUUCUGUUCCGCAATT |
|  | antisense: UUGCGGAACAGAAAGUCCCTT |
| negative control | sense: UUCUCCGAACGUGUCACGUTT |
|  | antisense: ACGUGACACGUUCGGAGAATT |
| miR-541-5p mimics | sense: AAAGGAUUCUGCUGUCGGUCCCACU |
|  | antisense: UGGGACCGACAGCAGAAUCCUUUUU |
| miR-541-5p inhibitor | AGUGGGACCGACAGCAGAAUCCUUU |
