# Supplementary material for: CircMTO1 suppresses hepatocellular carcinoma progression via the miR-541-5p/ZIC1 axis by regulating Wnt/β-catenin signaling pathway and epithelial-to-mesenchymal transition
Source: Cell Death Dis. 2021 Dec 20;13(1):12. doi: 10.1038/s41419-021-04464-3 (PMC8688446; doi:10.1038/s41419-021-04464-3)
Supplement: Supplementary file 8 — author-contribution-form [file 41419_2021_4464_MOESM8_ESM.pdf]

DECLARATION OF CONTRIBUTIONS TO ARTICLE

ADMC

Manuscript Number:	Journal Name:	
CDDIS-21-3437R	*Cell Death & Disease*	(the 'Journal')

Proposed Title of the Contribution:

CircMTO1 suppresses hepatocellular carcinoma progression via the miR-541-5p/ZIC1 axis by regulating Wnt/β-catenin signaling pathway and epithelial-to-mesenchymal transition (the 'Contribution)

Author(s):

Dandan Li1,2, Jiawei Zhang1,2, Jing Yang1,2, Jie Wang1,2, Runling Zhang1,2, Jinming Li*1,2,3, Rui Zhang*1,3 (the 'Authors')

For all *CDDis* articles, each person named as an author in the published version must be able to show he or she has contributed substantially to the article.

Authorship credit should be based on 1) substantial contributions to conception and design, acquisition of data, or analysis and interpretation of data; 2) drafting the article or revising it critically for important intellectual content; and 3) final approval of the version to be published. Authors should meet conditions 1, 2 and 3.

Any person who cannot be shown to have made a substantial contribution to the article cannot be listed as an author in the final version. The name of any person who is deemed to have made a minor contribution can, however, appear in the Acknowledgments section of the article.

Please complete the table below to indicate the contributions of all named authors to the manuscript.

Author Full Name:	Specification of Contribution to the Manuscript:
Dandan Li	contributed to conception and design,acquisition of data;contributed to drafting the article;approved the final version to be submitted
Jiawei Zhang	contributed to the analysis and interpretation of data;contributed to drafting the article; approved the final version to be submitted
Jing Yang	contributed to the analysis and interpretation of data;contributed to revising it critically for important intellectual content;approved the final version to be submitted
Jie Wang	contributed to acquisition of data;contributed to revising it critically for important intellectual content;approved the final version to be submitted
Runling Zhang	contributed to acquisition of data;contributed to revising it critically for important intellectual content;approved the final version to be submitted
Jinming Li	contributed to conception and design,acquisition of data;contributed to revising it critically for important intellectual content;approved the final version to be submitted
Rui Zhang	contributed to conception and design,acquisition of data;contributed tor revising it critically for important intellectual content;approved the final version to be submitted

Please complete the table below to indicate the contributions of all named authors to the figures.

Figure 1:

Dandan Li and Jiawei Zhang

Figure 2:

Dandan Li and Jiawei Zhang

Figure 3:

Jing Yang and Jie Wang

Figure 4:

Jie Wang and Runling Zhang

Figure 5:

Jinming Li and Dandan Li

Figure 6:

Jinming Li and Rui Zhang

Signed for and on behalf of the Author(s):	Print Name:	Date:
JinMing Li	Jinming Li	2021.11.11
